# Preparation of Deuterium Labeled Compounds by Pd/C-Al-D_2_O Facilitated Selective H-D Exchange Reactions

**DOI:** 10.3390/molecules27030614

**Published:** 2022-01-18

**Authors:** Anne Kokel, Dora Kadish, Béla Török

**Affiliations:** Department of Chemistry, University of Massachusetts Boston, 100 Morrissey Blvd, Boston, MA 02125, USA; annekokel@gmail.com (A.K.); dkadish@uci.edu (D.K.)

**Keywords:** H/D exchange, heterogeneous catalysis, green chemistry, Pd-Al combination, in situ deuterium gas formation

## Abstract

The chemo/regioselective H-D exchange of amino acids and synthetic building blocks by an environmentally benign Pd/C-Al-D_2_O catalytic system is described. Due to the importance of isotope labeled compounds in medicinal chemistry and structural biology, notably their use as improved drug candidates and biological probes, the efficient and selective deuteration methods are of great interest. The approach is based on selective H-D exchange reactions where the deuterium source is simple D_2_O. D_2_ gas is generated in situ from the reaction of aluminum and D_2_O, while the commercially available palladium catalyst assists the H-D exchange reaction. The high selectivity and efficiency, as well as the simplicity and safe nature of the procedure make this method an environmentally benign alternative to current alternatives.

## 1. Introduction

**Figure 1:**
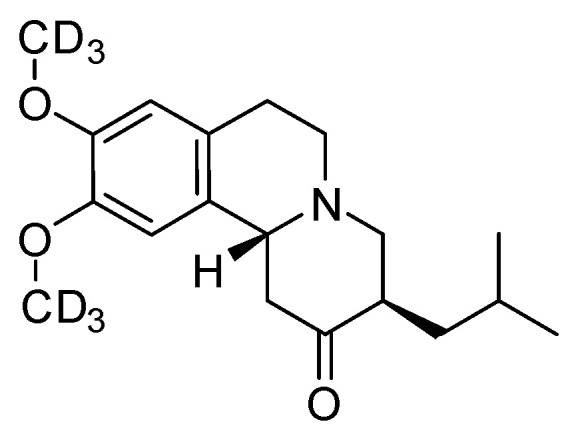
Structure of tetrabenazine-d_6_.

The selective H-D exchange of organic compounds, such as amines, alcohols or amino acids, offers great application opportunities either as drugs or as diagnostic tools. The benefits of deuterium labeling have long been considered to improve the properties of drugs [1]. While metabolic enzymes (e.g., proteases) have the ability to transform drug molecules to inactive metabolites, the introduction of deuterium to drugs appears to strengthen their resistance toward metabolic degradation due to the improved stability of the deuterium-carbon bond that is six to ten times stronger than the carbon-hydrogen counterpart [2]. In addition, the higher stability of the deuterated drug provides longer lasting effects, which allows lower dosage, likely causing fewer side effects [3,4]. As a breakthrough, in 2016, a deuterated drug developed by TEVA Pharmaceuticals, the *d_6_*-tetrabenazine (Figure 1) was approved to treat the symptoms of Huntington’s disease [5]. The deuterated analog demonstrated improved ADME properties compared to the original non-deuterated compound [6]. In fact, the deuterium introduced on the methoxy groups of the tetrahydroisoquinoline ring appears to prevent the cleavage of these substituents that leads to the inactive form of the drug [7].

The H-D exchange is also considered to stabilize chiral centers, slowing down the epimerization, which can be considerably useful when enantiomers have different potency and toxicity [8,9]. Isotope labeled drugs can also have other functions related to drug development. For instance, in imaging, where deuterium and hydrogen are discriminated, isotope labeled molecules can serve as biological tracers [10]. They can provide better understanding of metabolic pathways; they can also help localize the metabolites of drugs and help assess their toxicity. Deuterated amino acids, for example, are valuable candidates to be used as probes in biological organisms [11]. They can also be used for structural analysis of proteins [12].

Due to the significance of deuterated organic compounds, the development of efficient and selective deuteration methods attracted great interests [13,14]. Various combinations of deuterium source and reagents/catalysts are available to carry out deuteration reactions including acid-base catalyzed reactions [15], deuterated reagents-based methods (such as LiAlD_4_ [16]) and transition metal-catalyzed approaches [17,18,19]. Most methods, however, do not conform to the recent expectations and standards of sustainable synthesis. In addition to the metal deuteride reagents, that are used in excess and generate significant amount of toxic waste [10,16], there are three main deuterium sources employed for H-D exchange reactions. These are: D_2_ gas, D_2_O/H_2_ mixture and D_2_O. While versatile and efficient, procedures employing D_2_ gas as a deuterium source suffer from the drawbacks of the production of D_2_ gas that is particularly expensive and laborious. The H_2_/D_2_O system was reported for the H-D exchange of organic compounds using a palladium catalyst supported on carbon [20]. The methods achieved moderate to good yields and provided site-selectivity depending on the molecules. The transfer deuteration employing formic acid and D_2_O for the generation of D_2_ through H^+^/D^+^ exchange aided by an iridium catalyst was also achieved [21]. The reactions of various alcohols and ketones, performed under hydrogen atmosphere in D_2_O also afforded high H-D exchange yields [21]. While those methods are carried out at mild temperatures, protocols that do not require explosive gas handling are preferable due to safety concerns. The combination of a transition metal as catalyst and D_2_O as the deuterium source has also been widely used for H-D exchange reactions. Although some previous works described the use of harsh conditions to achieve high yields [22], the selection of appropriate catalysts under specific reaction conditions is a potentially sustainable and powerful method for H-D exchange. The selective H-D exchange of amines and amino acids in D_2_O was performed using a ruthenium catalyst [23]. High selectivity and high incorporation of deuterium were observed at 135 °C. Palladium, known for its high efficiency as a hydrogenation catalyst [24], was found to be a suitable catalyst for H-D exchange reactions as well, providing interesting reactivity. A regioselective palladium-catalyzed H-D exchange reaction was also reported [25]. The system provided excellent deuterium incorporation at the benzylic site under mild conditions. However, the use of a small amount of hydrogen gas was still necessary to reach higher H-D exchange yields. Tashiro et al. were among the first to develop a deuteration method based on Ni/Al alloy in D_2_O. The alloy exhibited high activity in an alkaline medium. For instance, various benzaldehydes, anilines and benzylamines were successfully deuterated with the Ni/Al alloy-Na_2_CO_3_-D_2_O system [26,27]. The amino acids tyrosine and phenylalanine were also subjected to this highly active catalytic system [28]. For both amino-acids, high overall H-D exchange but rather low selectivities were observed. In a recent study, photoredox catalysts have been applied for the selective H-D and H-T exchange of several compounds of pharmaceutical interest [29], indicating the continuously growing importance of isotope-labeled compounds. In a recent work, the selective halogen-deuterium replacement was applied for the incorporation of deuterium into important building blocks [30]. Due to the high interest in deuterated compounds, their preparation has been periodically reviewed; several recent accounts summarize the most recent developments in this field [31]. Building upon our recent efforts on the development of green synthetic methods [32,33], herein, we describe a simple and yet effective H-D exchange protocol for the selective labeling of a variety of compounds by using an environmentally benign Pd/C-Al-D_2_O system.

## 2. Results and Discussion

While the application of the Ni/Al alloy in reductive transformations has a considerable history, practical applications were hindered by the lack of selectivity [34]. By executing modifications to this system, we have been able to demonstrate its versatility in several transformations [35]. Recently, we demonstrated the success of the better tunable Pd/C-Al-water system for the chemoselective hydrogenation of a variety of organic compounds [36]. The simplicity and efficacy of the new protocol is based on the in situ hydrogen formation by the reaction of aluminum with water where the presence of palladium ensures the reduction in the substrates. In the present study, we extend the applicability of this system to H-D exchange reactions by replacing H_2_O by D_2_O. The continuous well-controlled in situ generation of D_2_ gas from D_2_O makes the method safe as the use of pressurized gas is not necessary, and it could be practical for both laboratory and industrial scale. The only waste generated is the stable and non-toxic aluminum oxide.

**Figure 2:**
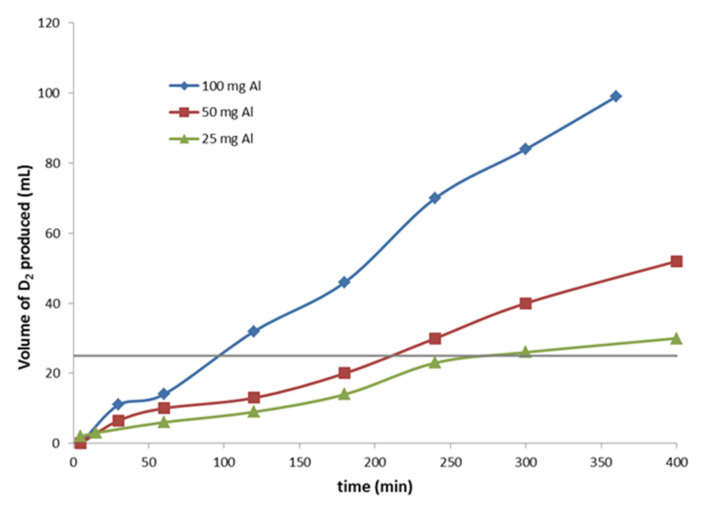
Volume of D_2_ produced as a function of time using various amount of Al at 80 °C (oil bath). The aluminum powder was mixed with 1.5 mL of D_2_O and was presonicated for 1 h before the measurement. The grey horizontal line indicates the formation of 1 mmol D_2_ for easier comparison.

As a preliminary experiment, the production of D_2_ gas with different amounts of aluminum was investigated in order to optimize the amount of aluminum employed. To do so, the volume of D_2_ generated was measured using a simple gas burette (see Supplementary Materials, Figure S1). The plotted results are depicted in Figure 2.

Figure 2 shows that increasing the amount of aluminum in parallel increases the amount of deuterium gas formed. In addition to the greater amount of D_2_, adding more aluminum will reduce the time needed to obtain a desired volume of D_2_ gas approximately by a factor of two under the applied conditions. After 120 min, 100 mg of Al initiated the formation of 30 mL of D_2_, while about 240 min were necessary to obtain the same amount of gas using 50 mg of Al. Nevertheless, independently of the time, even the lowest amount of Al produced more than 1 mmol of D_2_ at 80 °C which is a sufficient supply for the H-D exchange reactions given the scale at which they were performed (0.3 mmol). Following this experiment, the activity and selectivity of our Pd/C-Al-D_2_O system was investigated using a model substrate starting with 100 mg of Al to maximize the production of D_2_ in relatively short times. The major goal was to exchange C-H to C-D. The significantly more labile protons bound to N or O-atoms are also replaced during the procedure; however, after placing the compounds into water (or simply by moisture), they would exchange back to H rapidly. The amino acid L-phenylalanine was selected as a model substrate; it is an important amino acid, readily available, non-toxic and it possesses a variety of C-H bonds. The data are summarized in Table 1.

Microwave activation at 80 °C provided highly selective deuteration of the benzylic hydrogens, none of the other hydrogen atoms were exchanged, albeit the conversion was low (Table 1, entry 1). The increase in the reaction temperature enhanced the H-D exchange yield; the optimal temperature of 120 °C afforded complete H-D exchange of the benzylic hydrogens (Table 1, entry 5). Lower temperatures were not sufficient to reach quantitative yields (Table 1, entry 4). Further optimization of the reaction time revealed that 30 min at 120 °C provided the same results as the one obtained with 1 h of reaction time (Table 1, entry 6). As expected, based on Figure 2, reducing the amount of aluminum to 25 mg afforded similar results over a longer time (Table 1, entry 7). Therefore, the higher temperature combined with the microwaves appear to provide sufficient energy to ensure optimum formation of D_2_ gas with minimum amount of Al in a reasonable time. A decrease in the amount of the catalyst resulted in a significant drop in conversion; only 35% of the benzylic hydrogens was exchanged (Table 1, entry 8).

**Figure 3:**
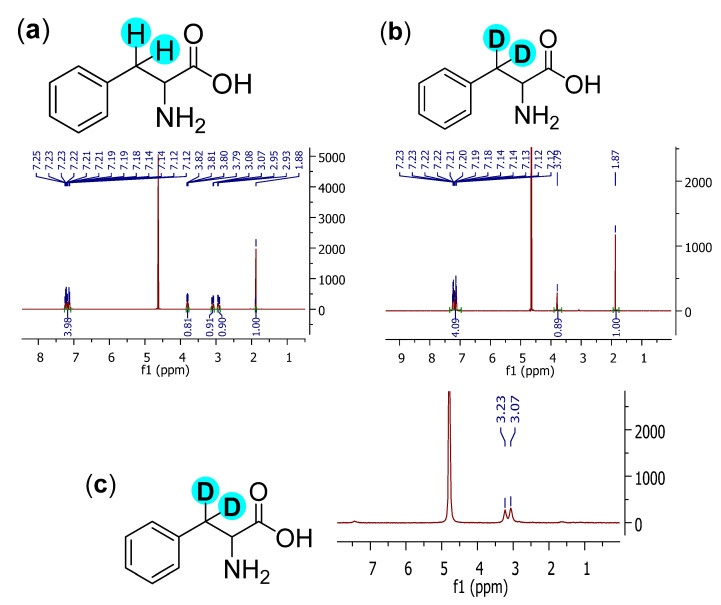
(**a**) ^1^H NMR spectrum of phenylalanine; (**b**) ^1^H NMR spectrum of d_2_-phenylalanine; (**c**) ^2^H NMR spectrum of d_2_-phenylalanine (the signal in (**a**,**b**) at 1.8 ppm belongs to the standard.).

It is important to note that various analytical methods were used to confirm the identity of the obtained deuterated products, such as the ^1^H NMR as well as ^2^H NMR spectroscopy and mass spectrometry. All methods were in agreement in the structural identification of the products. The ^1^H NMR spectra of L-phenylalanine before and after the H-D exchange exhibit unambiguous evidence for the disappearance of the benzylic protons. In addition, the ^2^H NMR confirms the presence of deuterium atoms in the corresponding position (Figure 3). The presence of the expected molecular-ion in the mass spectrum also confirmed the exchange of the two benzylic hydrogens to deuterium.

Encouraged by the high efficiency and selectivity of the H-D exchange of phenylalanine (Table 2, entry 1), the optimum reaction conditions were applied to other compounds in order to test the scope of the method. The tested compounds included amino acids and other aliphatic and aromatic building blocks frequently used in multistep synthesis. As a general observation, all substrates underwent high deuterium enrichment with high selectivity. The optimum temperature for phenylalanine had to be adjusted for each substrate. Glycine was deuterated in 90% exchange yield at 170 °C (Table 2, entry 2). While phenylalanine was deuterated selectively at the benzylic position, surprisingly, histidine was almost quantitatively and exclusively deuterated on its aromatic ring (Table 2, entry 3). Alanine required the highest temperature to provide a moderate deuterium exchange (60% exchange yield) of the hydrogen adjacent to the amino group (Table 2, entry 4). More than 90% of the hydrogen content remained on the methyl group. The deuteration of other compounds such as anilines, esters or alcohols is of great interests as well. Notably, they can serve as building blocks for the synthesis of compounds with medicinal relevance [36,37,38]. A few aniline derivatives were tested, and the conditions were optimized for each one of those substrates as well. Excellent deuterium enrichment was observed at the benzylic-CH_2_ position of 4-ethylaniline at mild temperature in 20 min (Table 2, entry 5). The other hydrogens underwent deuteration with about 25% yield only. A sample of 4,4′-(Ethane-1,2-diyl)dianiline was perdeuterated at 120 °C in 1 h (Table 2, entry 6). An aliphatic amine was also tested; phenylethylamine exhibited good H-D exchange on the CH_2_ position and no more than 15% for the other hydrogens (Table 2, entry 7). Next, the deuteration of the following two esters was investigated: diethyl malonate and diethyl methyl-malonate. The H/D exchange occurred in excellent yield at the most labile C-H positions of those molecules, providing selective deuteration. Deuteration yields of 99% and 80% were obtained, respectively, for the hydrogens in the position adjacent to both carbonyl groups (Table 2, entries 8 and 9). Less than 10% deuteration was observed for the other hydrogens of the carbon chain. It is worth noting that although the H-D exchange of malonic acid esters could be easily performed in alkaline medium (e.g., NaOD/D_2_O), the inevitable hydrolysis of the ester function and partial decarboxylation of the product malonic acid could occur, which can be avoided by using the present H-D exchange method. 3,5-dihydroxybenzyl alcohol also readily underwent deuteration on the aromatic ring with excellent selectivity and only a negligible amount of exchange occurred at the benzylic position (Table 2, entry 10).

In order to further characterize the products, particularly the chiral substrates, and gain more insight to the mechanism of the reaction, the potential effect of the method on chiral centers have been investigated. The chirality of the products phenylalanine-d_2_ (2-amino-3-phenylbutanoic-3,3-d_2_ acid) and alanine-d_1_ were determined. First, the amino acids were esterified with isopropyl alcohol, and the obtained esters were derivatized with (*S*)-Mosher’s chloride [39]. Then, the GC-MS profiles of the pre- and post-reaction amino acids were recorded (Figures S11 and S15). It was observed that although phenylalanine underwent the H-D exchange with retention of configuration, in the case of alanine nearly complete racemization occurred. These observations allow two conclusions regarding the participation of chiral compounds in these H-D exchange reactions. (i) First of all, it appears that when the exchange does not directly occur on the chiral C-H bond, the accidental exchange of the chiral C-H and racemization of the compound do not proceed. Thus, any such H-D exchange that can be carried out on benzylic, aromatic, etc. hydrogens will safely undergo with the retention of the original configuration. (ii) The only notable exemption to this rule is when the actual H-D exchange occurs on a chiral C-H bond; in these cases, racemization would follow. It is worth noting, however, that the second reaction takes place at very high temperatures, nearly 200 °C, indicating a high energy barrier for the racemization. All other H-D exchanges undergo at much lower temperatures.

**Figure 4:**
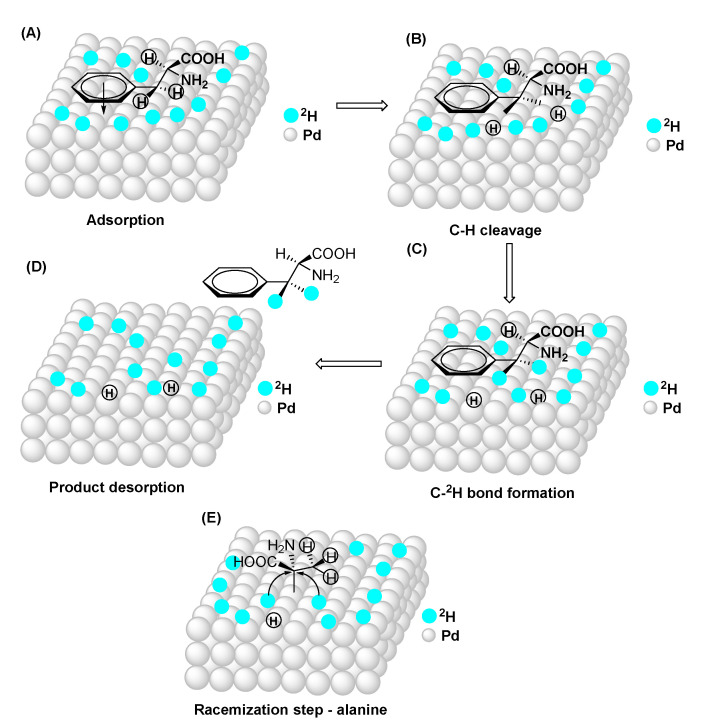
The proposed mechanism of the H-D exchange (**A**–**E**) (the atomic orbitals for Pd and ^2^H are for illustration only and not to scale).

The above experiments not only clarified the retention vs. racemization issue, but they also helped us to propose a likely mechanism for the reaction. As in most catalytic hydrogenolysis processes (particularly those of heterogeneous nature) the undesirable racemization of chiral substrates may occur. It appears that, based on the data, the key factor in whether the racemization occurs or not is the strength of the actual C-H bonds and the reaction temperature. Using phenylalanine, the exchange takes place at a relatively moderate temperature, where the energy requirement for the cleavage of the chiral aliphatic C-H bond was not met, hence there is no trace of exchange in that position and thus retention was observed. The racemization of alanine-d_1_ took place at a significantly higher temperature, which was able to provide the necessary activation energy for the deuterolysis of the chiral C-H bond. In agreement with the literature [40] and our own data, a multistep mechanism has been constructed that is depicted in Figure 4.

In short, in the first step, the deuterium gas and the target compound adsorb on the Pd surface (Figure 4A). For phenylalanine, the initial adsorption likely occurs via the delocalized aromatic electrons. Since the ring is planar, it keeps the neighboring benzylic CH_2_ close to the Pd surface, while the rest of the compound points away from the surface, hindering the breaking of those C-H bonds. Depending on the reaction temperature, the more or less sensitive C-H bonds will undergo a cleavage anchoring the actual C-atom to the Pd-surface (Figure 4B). This is followed by the deuterium insertion into the metal-C bond (Figure 4C) and the eventual desorption of the deuterated product (Figure 4D). In the case of multiple exchanges in a compound, the incorporation of the second deuterium atom may occur in a subsequent cleavage/exchange without actual desorption (adjacent hydrogens) or with desorption and re-adsorption (sterically distant C-H bonds). In the case of alanine, the surface binding occurs via the chiral carbon and during the C-^2^H bond formation the deuterium atoms can be inserted from both left and right side to yield racemic product (Figure 4E).

**Figure 5:**
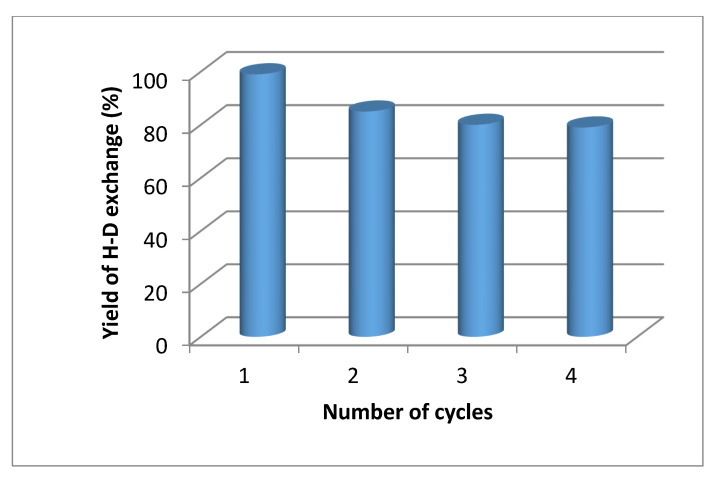
Yield (conversion × selectivity) of H-D exchange of phenylalanine to phenylalanine-d_2_ after reusing the same catalytic mixture (partially oxidized Al powder and Pd/C) replenished with fresh Al (25 mg) before each cycle for four consecutive cycles.

Last, with the aim of further reducing the environmental impact of our method, we investigated the recyclability of the catalytic system, as summarized in Figure 5. As the data show, the system exhibits good stability and recyclability. Although the reaction mixture had to be replenished with 25 mg of fresh aluminum before each cycle in order to ensure sufficient production of D_2_ gas, the transition metal demonstrated good recyclability, even though the catalyst loading is relatively low. As low as 3% mol catalyst successfully performed the H-D exchange reaction of phenylalanine to d_2_-phenylalanine without significant loss in activity: after the fourth cycle, above 80% of the catalyst activity was retained (see also Supplementary Materials, Figures S22 and S23). It is also remarkable that the selectivity appeared to remain steady throughout the four cycles.

## 3. Materials and Methods

### 3.1. General Information

All substrates, solvents and the Pd/C catalyst were purchased from Sigma-Aldrich and used without further purification. All H/D exchange reactions were carried out using a CEM Discover microwave reactor using closed-vessel setting at the temperatures noted. The ^1^H NMR spectra were recorded on a 400 MHz Agilent MR400DD2 spectrometer at 399.96 MHz, using acetic acid (or *tert*-butanol) as the internal standard to calculate the yield of H-D exchange and confirm that no other structural changes occurred in the compounds. The ^2^H NMR spectra were recorded on the same instrument at 61.4 MHz frequency. The products were also characterized by gas chromatography—mass spectrometry (GC-MS) with an Agilent 6850 gas chromatograph-5973 mass spectrometer system (70 eV electron impact ionization) using a 30 m long DB-5 type column (J&W Scientific, Folsom, CA, USA). The high resolution mass spectrometry analysis (HR-MS) was performed using an AB SCIEX Qtrap 5500 instrument in negative ion mode. 

### 3.2. Procedure

A microwave reaction vessel was charged with aluminum powder (100 mg) and 5% Pd/C catalyst (20 mg) and suspended in 1.5 mL of D_2_O. The vessel was placed in an ultrasonic bath for 1 h. The substrate (0.200 mmol) was added to the reaction vessel before irradiating the reaction mixture in a CEM Discover microwave reactor. After completion of the reaction, 0.5 mL of the reaction mixture was mixed and stirred thoroughly with 0.5 mL of a stock solution containing the NMR internal standard (40 μL of acetic acid or *tert*-butanol in 10 mL of D_2_O). The NMR samples were prepared from the combined solutions. The yields of H-D exchange were calculated using the ratio of the integration of a given signal to the integration of the internal standard’s signal. The ratios obtained for the deuterated molecule were then compared to the ratios obtained with the hydrogenated-counterpart in order to determine the H-D exchange yield. Calibration curves were drawn from an experiment with various amount of phenylalanine to assess the accuracy of the internal standard method. A fairly linear function was obtained demonstrating the validity of the method to calculate H-D exchange yields (see Supplementary Materials, Figures S2 and S3).

### 3.3. Catalyst Recycling

The reaction mixture was centrifuged after each cycle. The solution containing the substrate was removed and analyzed. To the remaining solid mixture (Al powder and Pd/C) 1.5 mL of fresh D_2_O was added and the mixture was centrifuged again. After this step of rinsing, the liquid layer was discarded. A clean microwave reaction vessel was charged with the remaining solid mixture containing the catalyst and was supplemented with 25 mg of fresh aluminum powder before adding a new portion of (1.5 mL) D_2_O. The reaction mixture was then sonicated for 1 h before adding the substrate and carrying out the microwave-driven reaction. The conditions were the same as the optimum conditions found for the model substrate, phenylalanine.

**2,2-d_2_-phenylalanine** (Table 2, entry 1): ^1^H NMR (D_2_O, 400 MHz): δ (ppm) = 3.79 (s, 1H), 7.12–7.23 (m, 5H); ^2^H NMR (D_2_O, 61.4 MHz): δ (ppm) = 3.23 (s, 1D), 3.07 (s, 1D); MS (EI): C_8_H_9_D_2_N *m*/*z* = 168 (M^+^H).

**d_2_-glycine** (Table 2, entry 2): ^1^H NMR (D_2_O, 400 MHz): δ (ppm) = 3.39 (s, 2H). ^2^H NMR (D_2_O, 61.4 MHz): δ (ppm) = 3.72 (s, 2D); MS (EI): C_2_H_3_D_2_NO_2_ *m*/*z* = 78 (M^+^H).

**d_2_-histidine** (Table 2, entry 3): ^1^H NMR (D_2_O, 400 MHz): δ (ppm) = 2.96–3.10 (m, 2H), 3.78–3.82 (t, 1H). ^2^H NMR (D_2_O, 61.4 MHz): δ (ppm) = 6.96 (s, 1D), 7.63 (s, 1D); MS (EI): C_6_H_7_D_2_N_3_O_2_ *m*/*z* = 158 (M^+^H).

**d_1_-alanine** (Table 2, entry 4):^1^H NMR (D_2_O, 400 MHz): δ (ppm) = 1.31 (s, 3H), 3.62–3.64 (m, 1H). ^2^H NMR (D_2_O, 61.4 MHz): δ (ppm) = 1.37 (s, 1D), 3.70 (s, 1D); HR-MS (ESI-TOF) (M^-^): C_3_H_6_DO_2_N value found = 89.9924, calculated value = 90.0540.

**d_2_-ethylaniline** (Table 2, entry 5):^1^H NMR (D2O, 400 MHz): δ (ppm) = 1.02 (s, 3H), 6.98–7.17 (m, 1H). ^2^H NMR (D_2_O, 61.4 MHz): δ (ppm) = 2.34 (s, 2D); MS (EI): C_8_H_9_D_2_N *m*/*z* = 108 (100%), 123 (M^+^, 34%).

**d_12_-4,4’-(ethane-1,2-diyl)dianiline** (Table 2, entry 6):^1^H NMR (D_2_O, 400 MHz): δ (ppm) = 1.02 (s, 3H), 6.98–7.17 (m, 1H). ^2^H NMR (D_2_O, 61.4 MHz): δ (ppm) = 2.34 (s, 2D); MS (EI): C_14_H_4_D_12_N_2_ *m*/*z* = 109 (100%), 220 (13%), 224 (M^+^, 1%).

**d_2_-phenylethylamine** (Table 2, entry 7): ^1^H NMR (D_2_O, 400 MHz): δ (ppm) = 2.72 (s, 2H), 7.11–7.24 (m, 5H). ^2^H NMR (D_2_O, 61.4 MHz): δ (ppm) = 2.89 (s, 4D), 6.87–7.25 (m, 8D); MS (EI): C_8_H_8_D_2_N *m*/*z* = 93 (100%), 123 (M^+^, 24%).

**d_2_-diethyl malonate** (Table 2, entry 8): ^1^H NMR (D_2_O, 400 MHz): δ (ppm) = 1.10–1.14 (t, 6H), 1.52 (q, 4H). ^2^H NMR (D_2_O, 61.4 MHz): δ (ppm) = 3.46 (s, 2D); MS (EI): C_8_H_10_D_2_O_4_ *m*/*z* = 117 (100%), 162 (M^+^, 2%).

**d_1_-diethyl methylmalonate** (Table 2, entry 9): ^1^H NMR (D_2_O, 400 MHz): δ (ppm) = 1.07–1.10 (t, 6H), 1.20 (s, 3H), 4.02–4.08 (q, 4H). ^2^H NMR (D_2_O, 61.4 MHz): δ (ppm) = 2.91 (s, 2D); MS (EI): C_8_H_13_DO_4_ *m*/*z* = 130 (100%), 175 (M^+^, 15%).

**d_1_-3,5-dihydroxybenzyl alcohol** (Table 2, entry 10): ^1^H NMR (D_2_O, 400 MHz): δ (ppm) = 4.33 (s, 2H), 6.15 (s, 1H), 6.27 (s, 2H). ^2^H NMR (D_2_O, 61.4 MHz): δ (ppm) = 6.17–6.28 (m, 3D); MS (EI): C_7_H_7_DO_3_ *m*/*z* = 141 (100%), 143 (M^+^, 40%).

## 4. Conclusions

In conclusion, a selective, practical and environmentally friendly H-D exchange method was developed. A range of substrates from amino acids and amines to esters and alcohols were successfully deuterated under microwave-assisted conditions. The major advantages of this method are as follows: (i) the combination Pd/C-Al-D_2_O that provides a safe way to generate D_2_ in situ, (ii) the only waste generated in the reaction is the stable and non-toxic aluminum oxide, (iii) the availability of the catalyst that is ligand-free and nevertheless, provides excellent selectivity, and (iv) a rapid synthesis ensured by the microwave irradiation. It is reasonable to conceive that the procedure described herein may have the potential to contribute to improvements in the drug design process in terms of cost, time and environmental impact.

## Figures and Tables

**Table 1:** Optimization of the reaction conditions for the H-D exchange using phenylalanine as a model substrate ^a^.

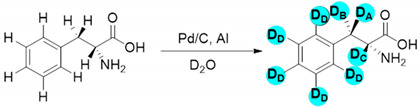								
**Entry**	**T (°C)**	**Time (h)**	**∆**	**Conversion (%)**	**H-D Exchange (%) ^e^**			
					**D_A_**	**D_B_**	**D_C_**	**D_D_**
1	80	1	MW	55	55	20	0	0
2	80	1	MW ^b^	0	0	0	0	0
3	80	24	CH	100	100	75	0	15
4	100	1	MW	75	75	30	0	0
5	120	1	MW	100	100	100	0	0
6	120	0.5	MW	100	100	100	0	0
7	120	1	MW ^c^	100	100	100	0	0
8	120	0.5	MW ^d^	35	35	0	0	0

^a^ Reaction conditions: 0.3mmol of substrate, 20 mg of 5% Pd/C, 100 mg of Al powder in 1.5 mL of D_2_O. 1 h of presonication of the catalytic mixture (Pd/C-Al in D_2_O) before adding the substrate and irradiating the mixture under microwaves. Note: Three control reactions (1) without Pd and Al, (2) without Pd, and (3) without Al were carried out, and none of them yielded any H-D exchange; ^b^ reaction without presonication of the catalyst mixture; ^c^ reaction with 25 mg Al ^d^ reaction with 10 mg of Pd/C; ^e^ determined by ^1^H NMR (see Supplementary Materials, Figures S4–S9).

**Table 2:** Synthesis of deuterium labeled compounds via H-D exchange reactions by Pd/C-Al-D_2_O system ^a^.

Entry	Product	Time (min)	T (°C)	Conversion ^b,c^ (%)	ED ^d^ (%)
1	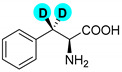	30	120	100	>99
2	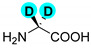	60	170	90	90
3	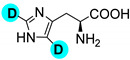	20	120	95	95
4	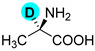	60	190	60	60
5	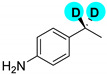	20	60	100	>99
6	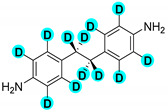	60	120	100	>99
7	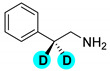	60	80	80	80
8	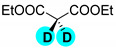	60	100	100	>99
9	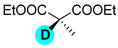	60	80	80	80
10	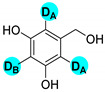	60	120	100	D_A_: 76 D_B_: 99

^a^ Reaction conditions: 0.3 mmol of substrate, 3% of Pd/C, 100 mg of Al powder in 1.5 mL of D_2_O (25 mg of aluminum afforded similar results with a few percent drop of yield for some substrates—see Supplementary Materials, Figures S10–S21. Reaction was carried out under microwave after 1 h of presonication of the catalytic mixture; ^b^ determined by NMR (see Supplementary Materials, Figures S12–S14); ^c^ The isolated yields in each case were >99%, essentially recovering the starting material in a D-enriched form; ^d^ Deuterium enrichment.

## Data Availability

Data is contained within the article or supplementary material.

## Supplementary Materials

The Supplementary Material can be downloaded online. The following supporting information includes additional data, including the compound spectra, determination of chirality etc. as reference to in the article.

## Sample Availability

Samples of the compounds are not available from authors. Synthetic details are in the experimental section.
