# Supplementary material for: Preparation of Deuterium Labeled Compounds by Pd/C-Al-D2O Facilitated Selective H-D Exchange Reactions
Source: Molecules. 2022 Jan 18;27(3):614. doi: 10.3390/molecules27030614 (PMC8840159; doi:10.3390/molecules27030614)
Supplement: Supplementary file 1 [file molecules-27-00614-s001.zip › molecules-1551869-supplementary.pdf]

Supporting Information

Preparation of deuterium labeled compounds by Pd/C-Al-D_2O facilitated selective H-D exchange reactions

Table of content

D_2 gas production experiment (Fig. S1).……………………………………………………………………………p.1,2

Calibration of the quantitative determination of hydrogen content method in ^{1}H NMR (Fig. S2 and 3)………p.3,4

Optimization of the reaction conditions

H-D exchange of phenylalanine for 24h at 80°C under conventional heating (Fig. S4)………p.5
H-D exchange of phenylalanine for 1h under MW at 80°C (Fig. S5)…………………………………..p.6
H-D exchange of phenylalanine for 1h under MW at 100°C (Fig. S6) ……..…………………………p.7
H-D exchange of phenylalanine for 1h under MW at 120°C (Fig. S7)……………………………….…p.8
H-D exchange reaction of phenylalanine for 20 min under MW at 120°C (Fig. S8)……………..p.9
H-D exchange of phenylalanine with 10mg of catalyst and 50mg of aluminum (Fig. S9)…..p.10

H-D exchange of amino acids (^{1}HNMR, ^{2}HNMR and MS)

Phenylalanine (Fig. S10 and 11)……………………………………………………………………………………..p.11-16
Glycine (Fig. S12.)………..p.17-19
Histidine (Fig. S13)………..p.20-22
Alanine (Fig. S14 and 15)……………………………………………………………………………………………………p.23-28

H-D exchange of amines, esters and alcohols (^{1}HNMR, ^{2}HNMR and MS)

Ethylaniline (Fig. S16)……p.29-32
Ethylenedianiline (Fig. S17)………………………………………………………………………………………………..p.33-36
Phenylethylamine (Fig. S18)………………………………………………………………………………………………..p.37-40
Diethyl malonate (Fig. S19) …………………………………………………………………………………………………..p.41-43
Diethylmethyl malonate (Fig. S20)…………………………………………………………………………………..…..p.44-47
3,5dihydroxybenzyl alcohol (Fig. S21)……………………………………………………………………..…………..p.48-51

Recycling of catalytic system (Fig. S22 and 23).……………………………………………………………………p.52-54

D_2 gas production experiment

$$2Al + 3D_2O \rightarrow Al_2O_3 + 3D_2$$

Amount of Al (mmol)

- 100mg: $n(Al) = \frac{m(Al)}{M(Al)} = \frac{100mg}{\frac{27g}{mol}} = 3.7mmol$
- 50mg: $n(Al) = \frac{m(Al)}{M(Al)} = \frac{50mg}{\frac{27g}{mol}} = 1.9mmol$
- 25mg: $\mathrm{n}(Al) = \frac{m(Al)}{M(Al)} = \frac{25mg}{\frac{27g}{mol}} = 0.92mmol$

According to the above equation, the maximum amount of gas that can be produced is:

- 100mg: $n(D_2) = \frac{3}{2} \times 3.7mmol = 5.6mmol$
- 50mg: $n(D_2) = \frac{3}{2} \times 1.9mmol = 2.9mmol$
- 25mg: $n(D_2) = \frac{3}{2} \times 0.92mmol = 5.55mmol$

➢ **Measurements of volume of gas produced under conventional heating**

The volume of gas produced was measured over 6h at 80°C using various amount of Al in 1.5mL of D_2O:

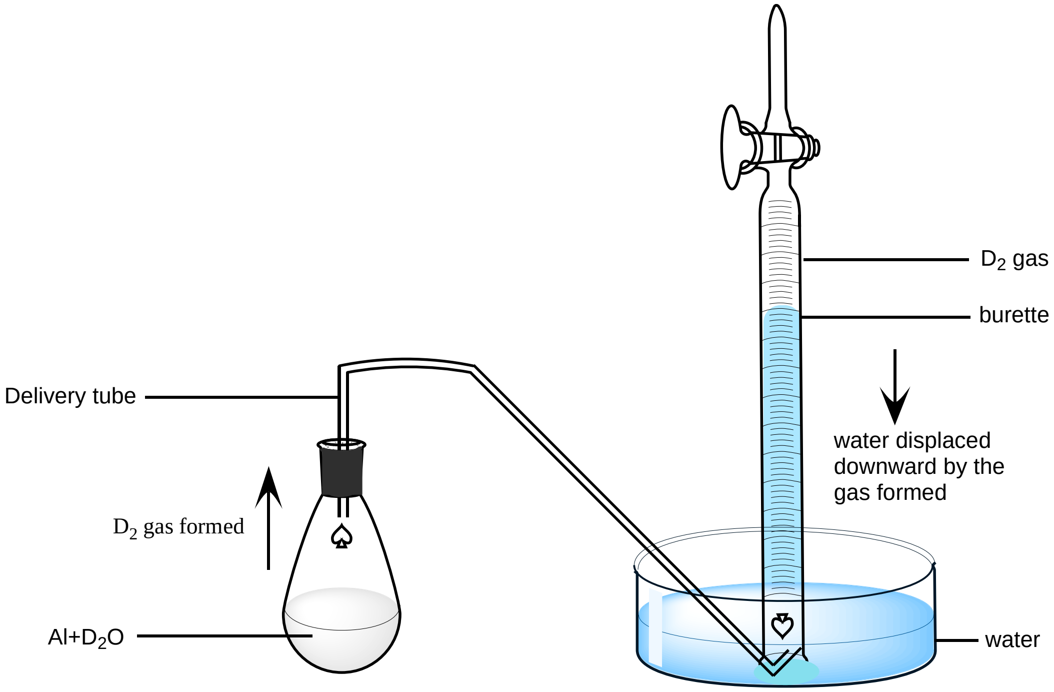

Figure S1. Measurement of the volume of D_2 gas formed at 80°C

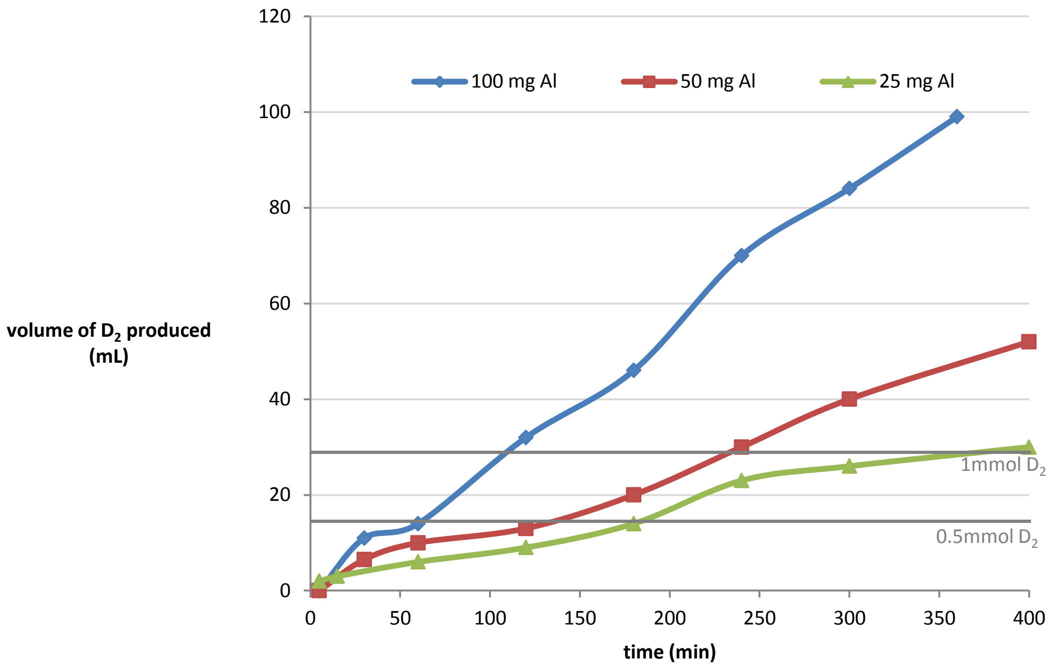

Figure 1 (from article): Volume of D_2 produced over time using various amount of Al at 80°C (oil bath).

Assuming that D_2 gas follows the ideal gas Law

After 6h using 25mg of Al, about 30mL of gas was produced.

$$PV = nRT$$

$$n(D_2) = \frac{P \times V}{R \times T} \; with \; R = 0.08206 \; L \; atm \; mol^{-1} K^{-1}$$

$$n(D_2) = \frac{1 \; atm \times 0.030 \; L}{0.08206 \; L \; atm \; mol^{-1} K^{-1} \times 353K} = 1.0 \; mmol$$

Figure S2. Calibration of the quantitative determination of hydrogen content method in ^{1}H NMR

40µL of acetic acid (the external standard) was added to 10mL of D_2O. 1mL of this stock solution was added to 1mL of phenylalanine solution in a vial and stirred up for 2min. 0.7mL of this mixture was taken for NMR analysis. For each substrate, a reference spectrum was generated and used as a comparison to any spectrum obtained after H-D exchange reaction. The deuterium content was calculated based on the ratio of the integration of hydrogen before H-D exchange to the integration of hydrogen after H-D exchange, both standardized with the acetic acid signal. Note that a new reference spectrum had to be taken when using a different standard stock solution to reduce additional errors.

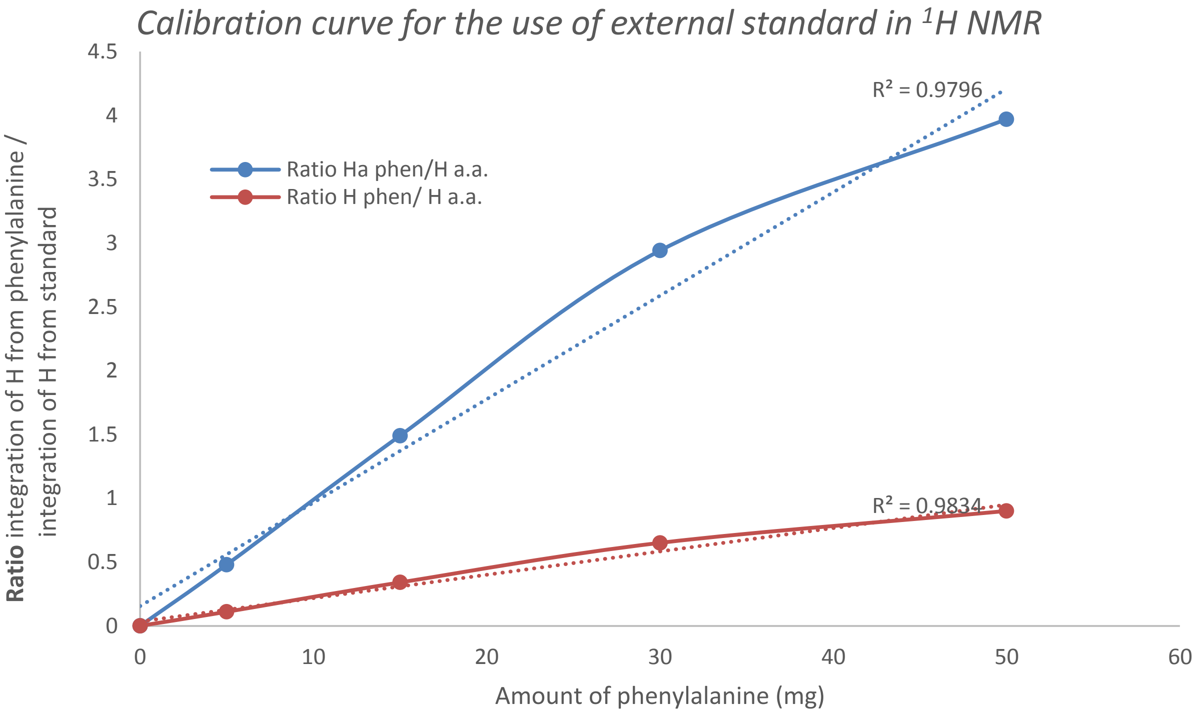

Amount of phenylalanine (mg)	Ratio H_A/H a.a.	Ratio H/H a.a.
0	0	0
5	0.48	0.11
15	1.49	0.34
30	2.94	0.65
50	3.97	0.9

H=H_B, H_C, H_D; a.a.=acetic acid (standard)

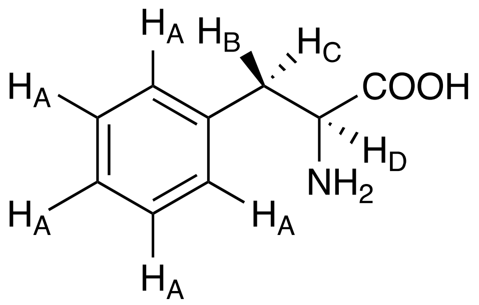

Figure S3. ^{1}**H NMR spectra for the calibration of the quantitative determination of hydrogen content method using acetic acid as the external standard and phenylalanine**

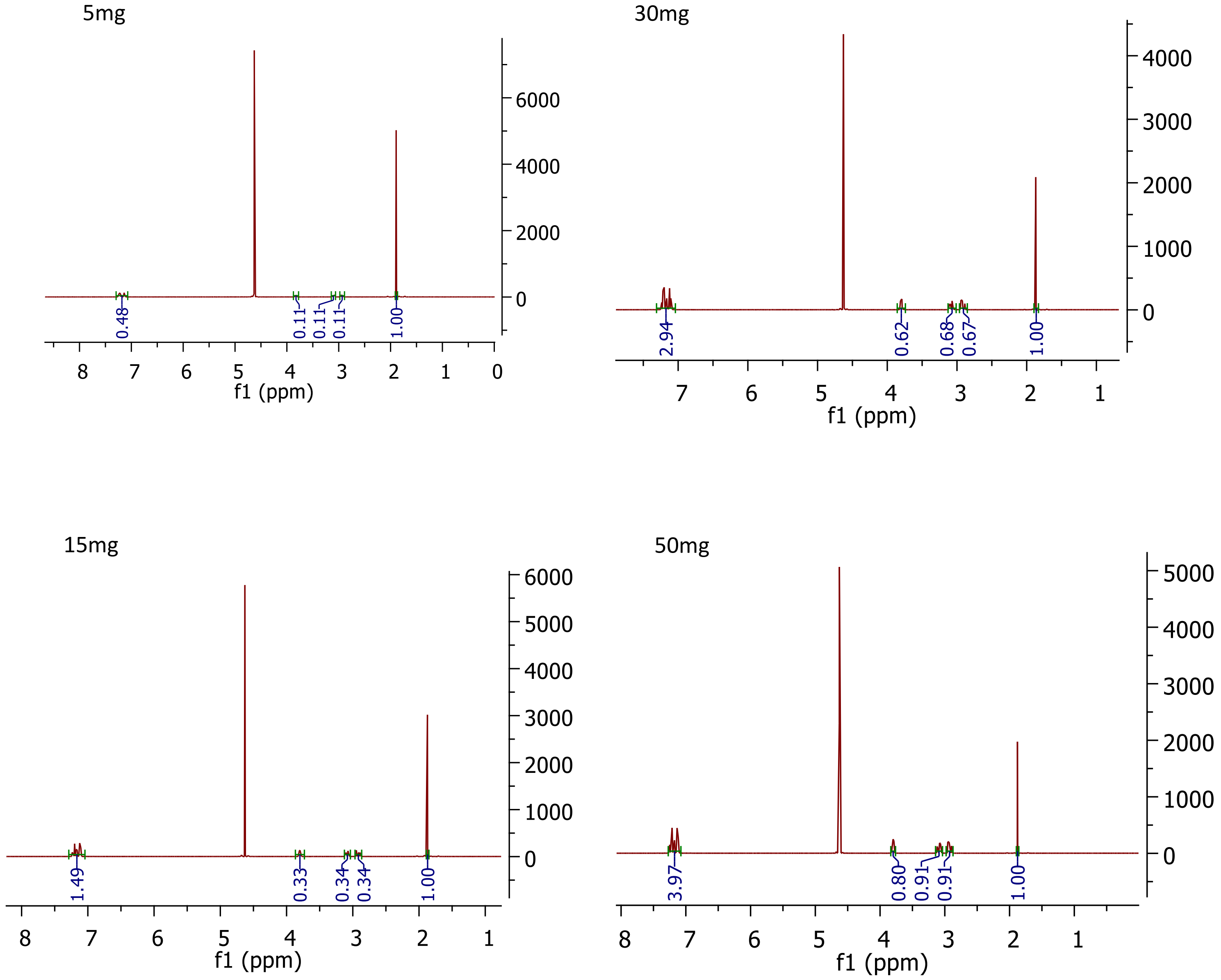

Figure S4. H-D exchange reaction of phenylalanine for 24h at 80°C under conventional heating

(20mg of Pd/C, 100mg of Al)

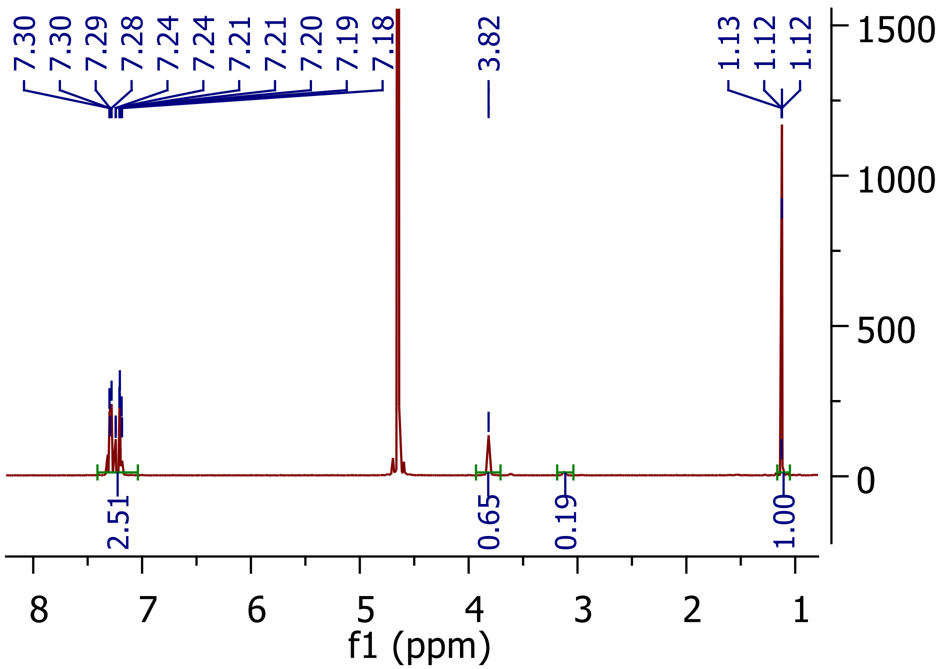

reference

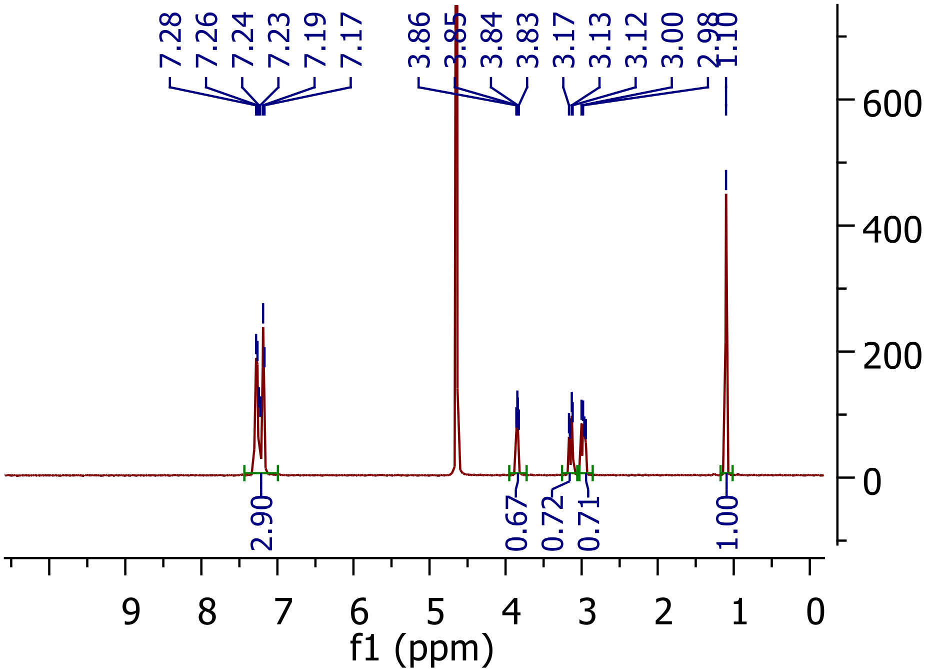

Figure S5. H-D exchange reaction of phenylalanine for 1h under MW at 80°C

(20mg of Pd/C, 100mg of Al)

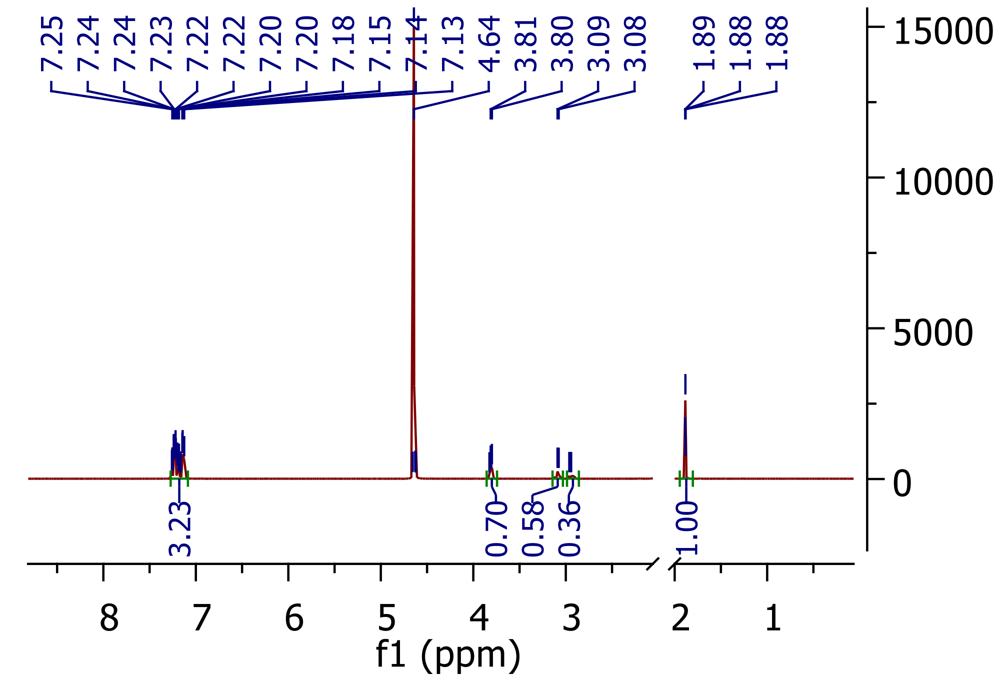

reference

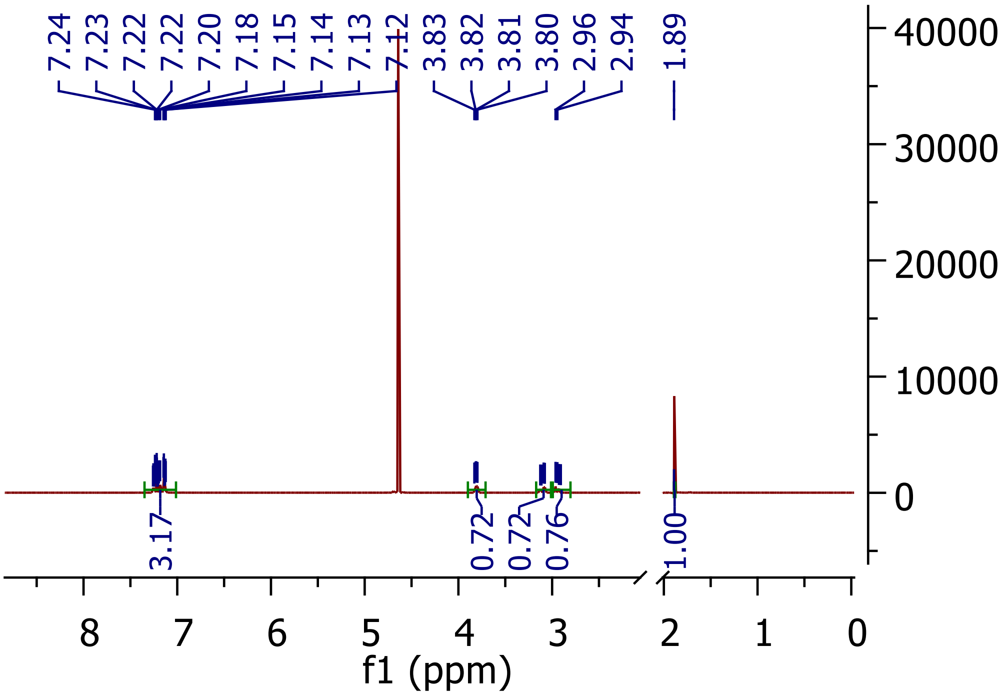

Figure S6. H-D exchange reaction of phenylalanine for 1h under MW at 100°C

(20mg of Pd/C, 100mg of Al)

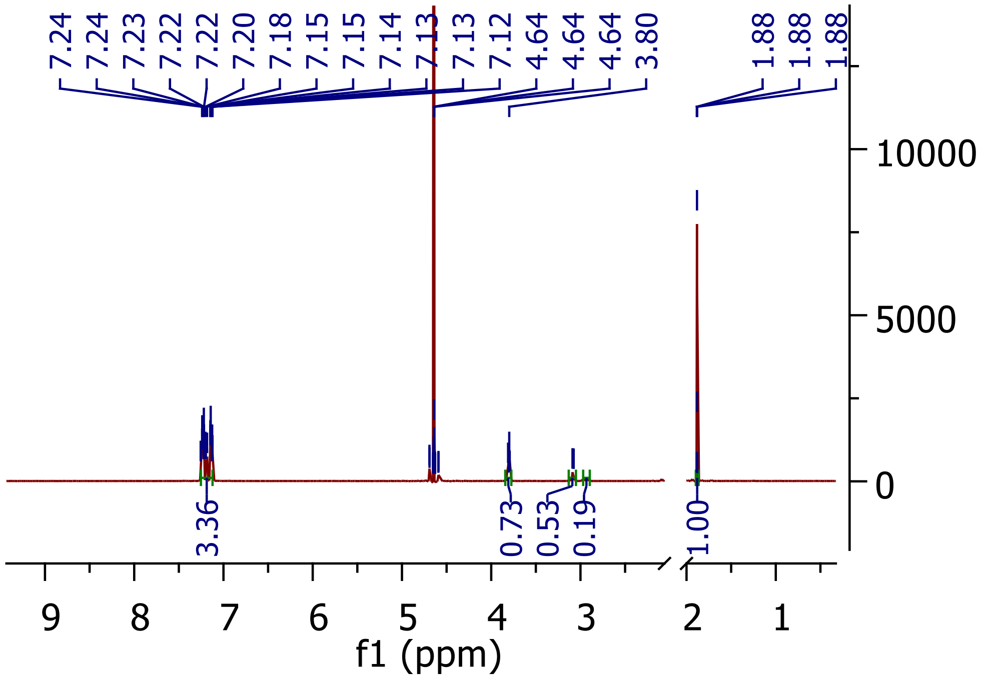

reference

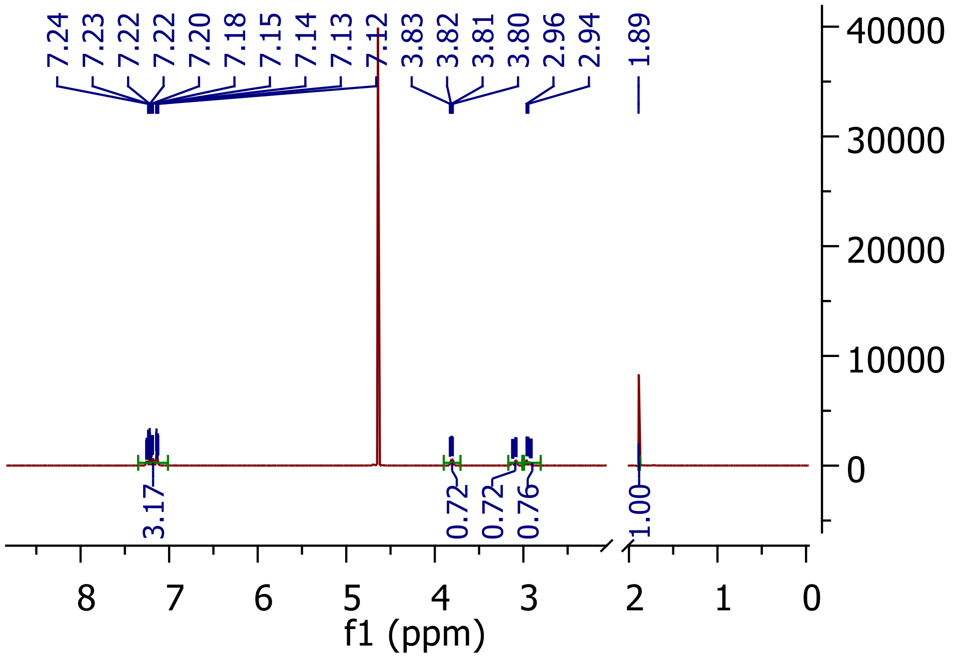

Figure S7. H-D exchange reaction of phenylalanine for 1h under MW at 120°C

(20mg of Pd/C, 100mg of Al)

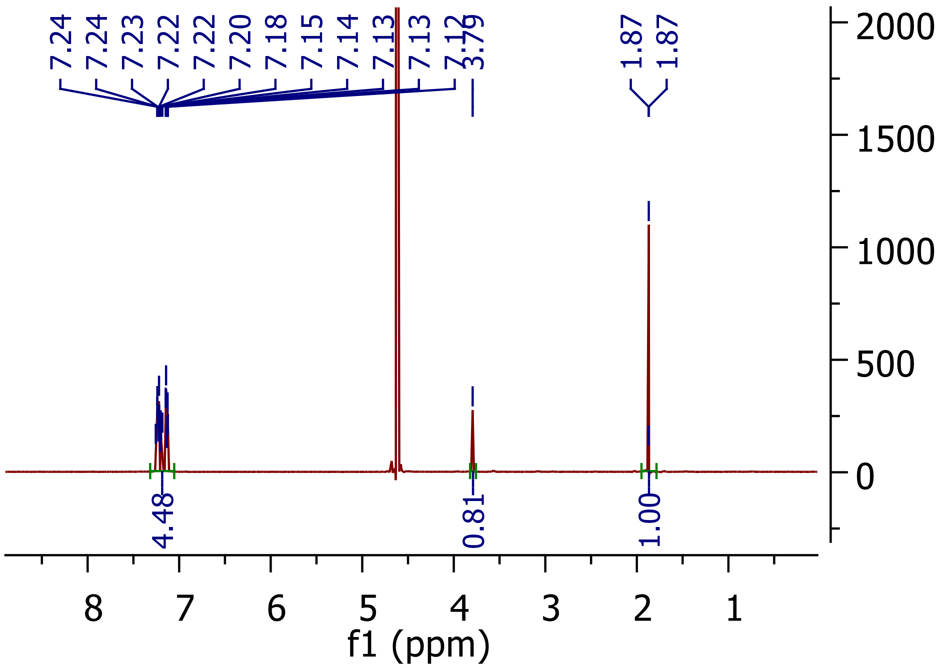

reference

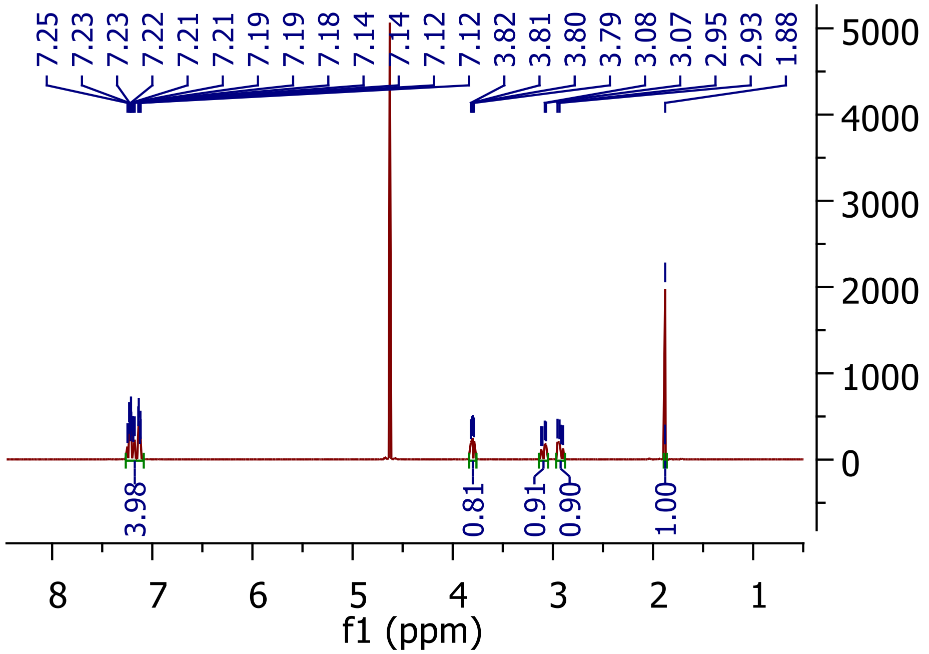

Figure S8. H-D exchange reaction of phenylalanine for 20 min under MW at 120°C

(20mg of Pd/C, 100mg of Al)

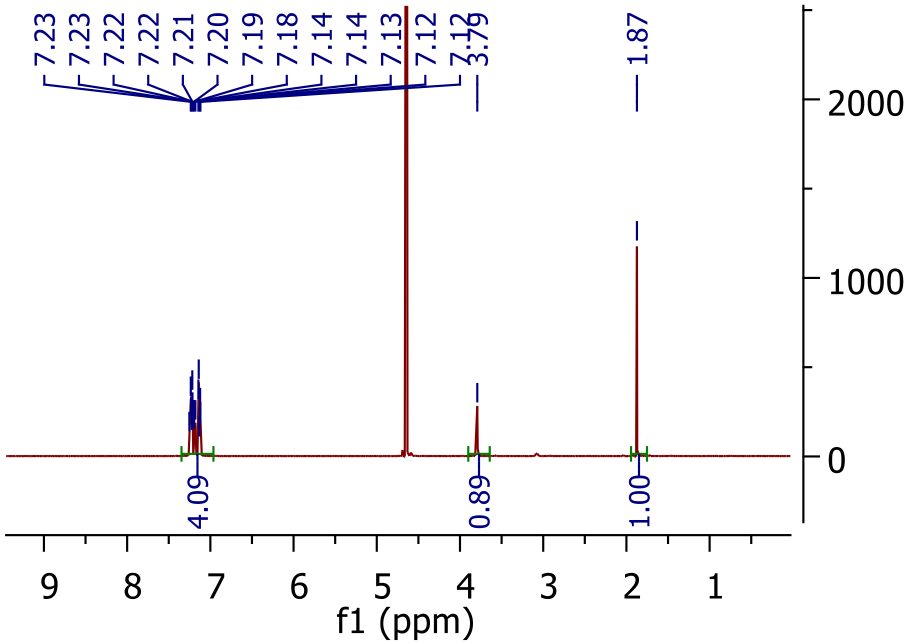

reference

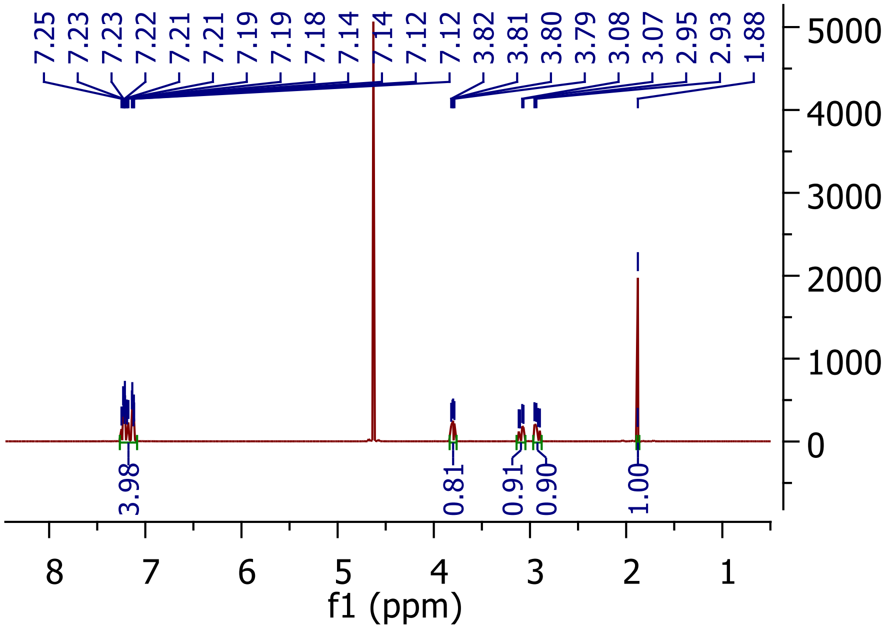

Figure S9. H-D exchange reaction of phenylalanine with 10mg of catalyst and 100mg of Al

(1h, MW, 120℃)

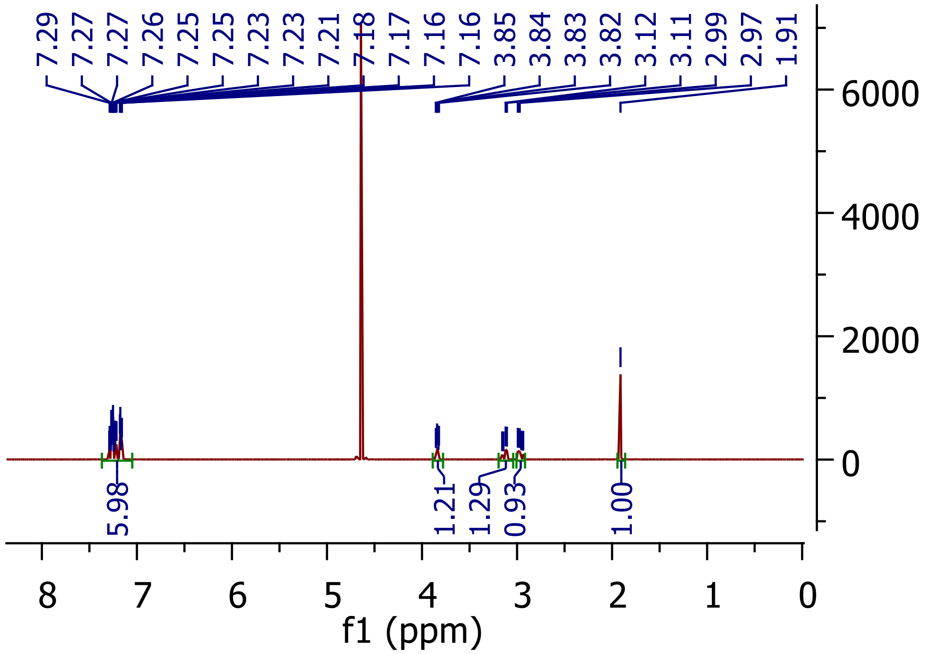

reference

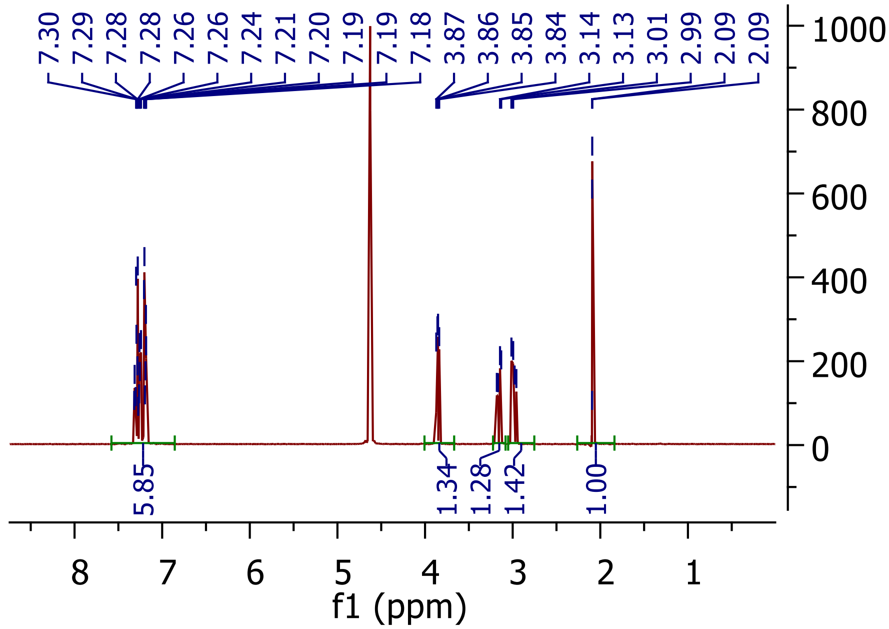

Figure S10. H-D exchange reaction of phenylalanine

(20mg of Pd/C, MW, 120℃)

a. ^{1}H NMR

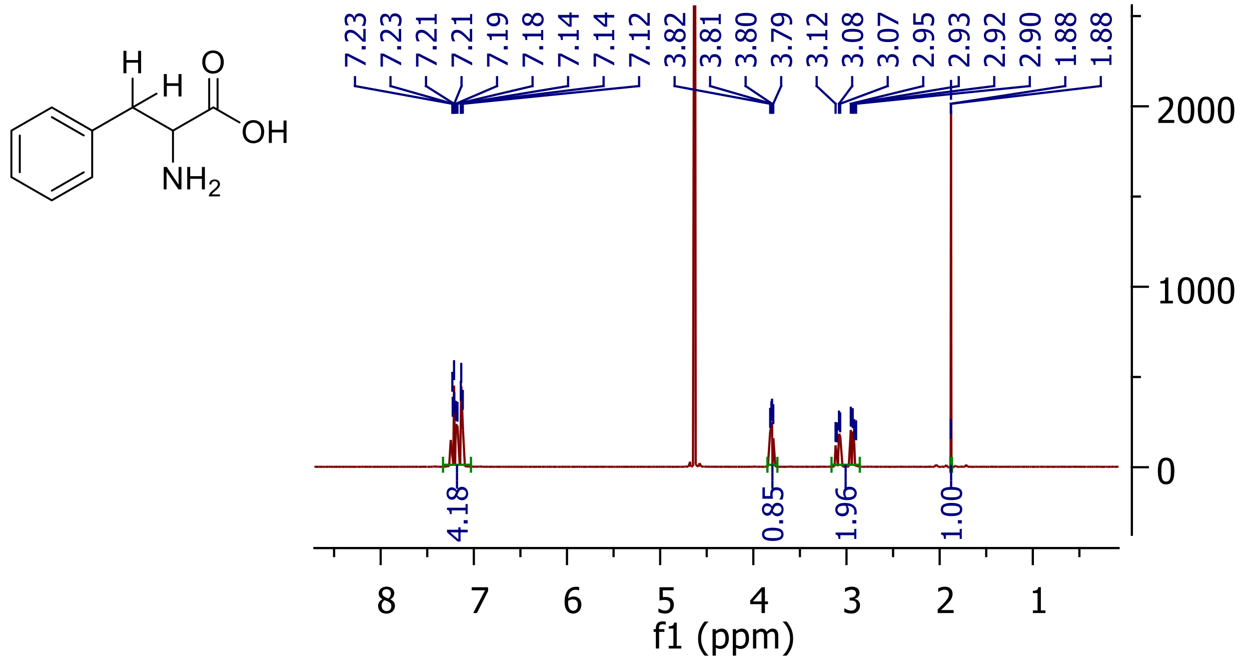

100mg of Al, 20min reaction

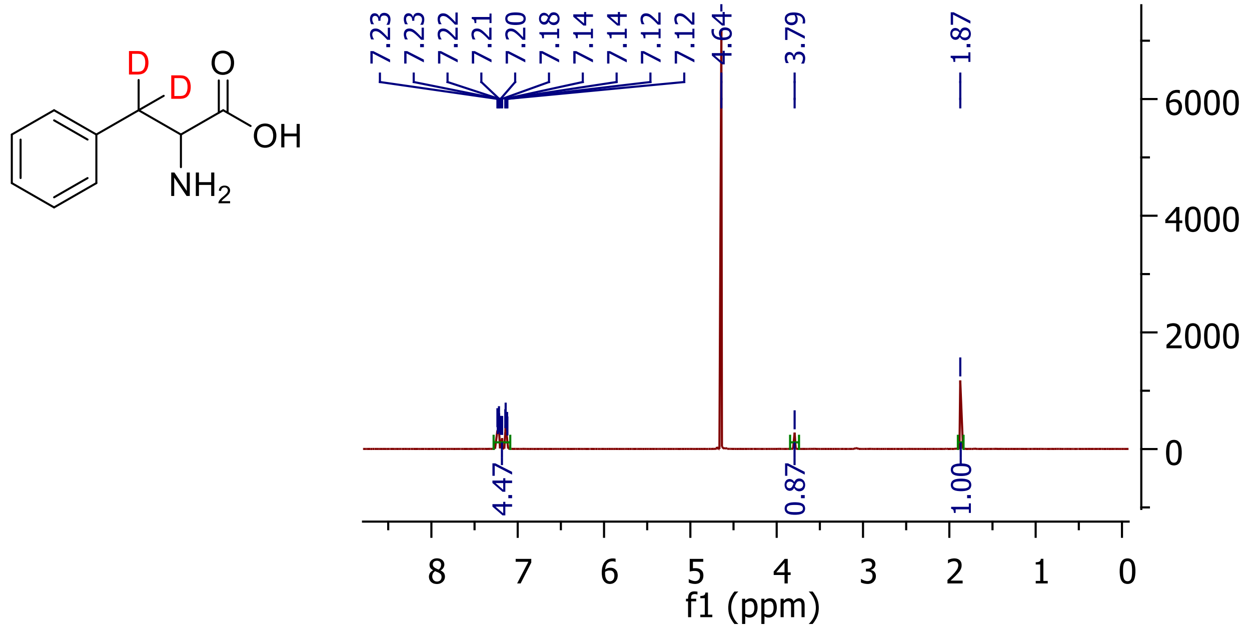

25mg Al, 60min reaction

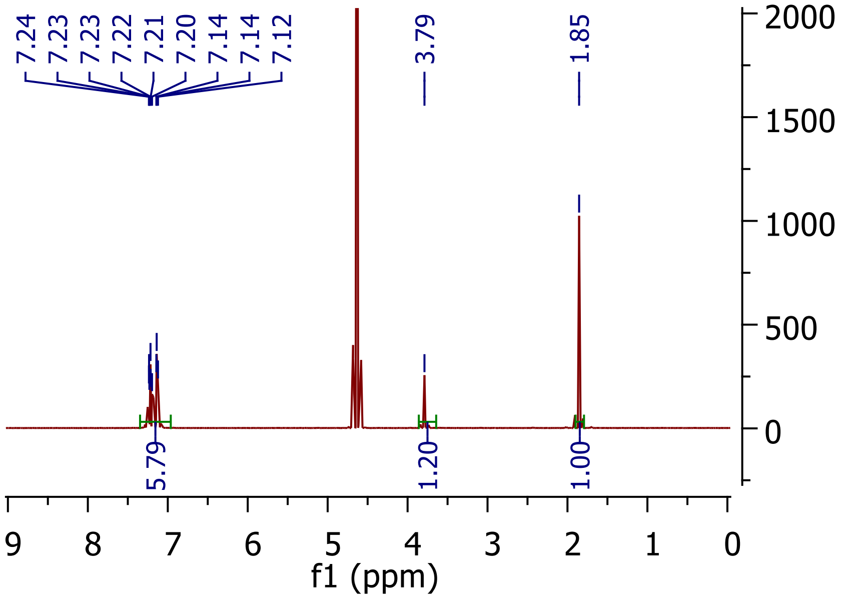

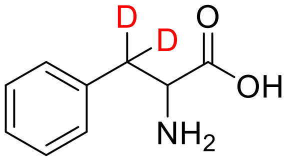

b. ^{2}H NMR

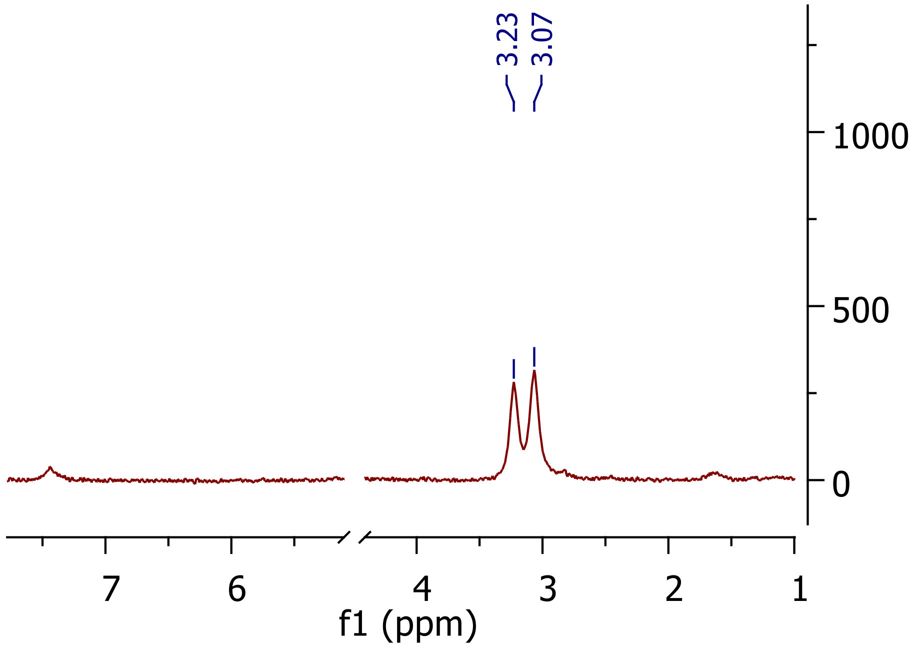

c. **Mass Spectrum**

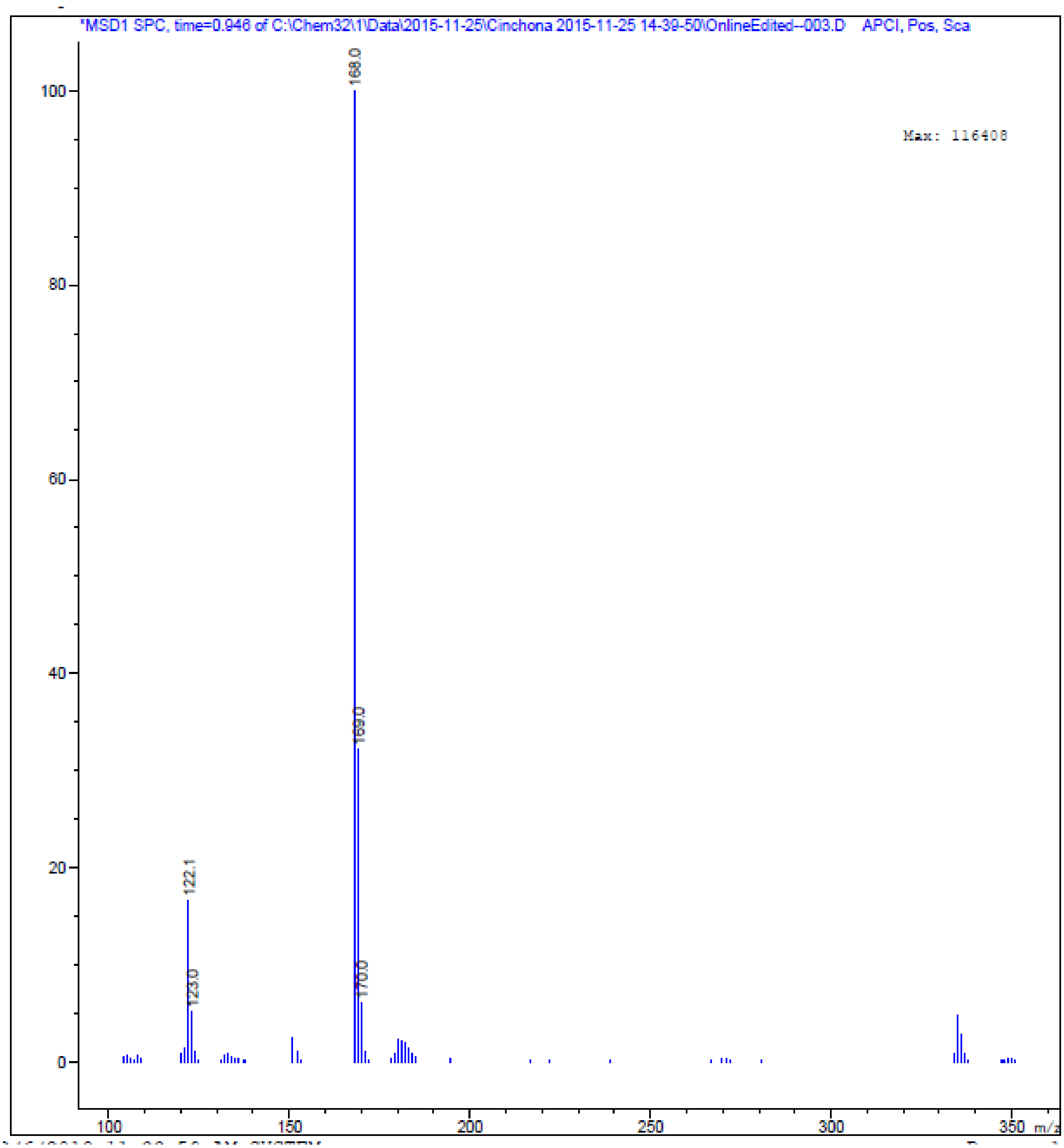

Figure S11. Determination of the chirality of deuterated phenylalanine

Phenylalanine was derivatized using isopropyl alcohol to form an amino acid ester first and then it was reacted with Mosher's chloride to functionalize the amino group; before it was injected in the GC-MS. The first chromatogram shows the separation of a pseudo racemic mixture of phenylalanine. The mass spectrum of both products was identical. The second chromatogram corresponds to the derivatized d_2-phenylalanine.

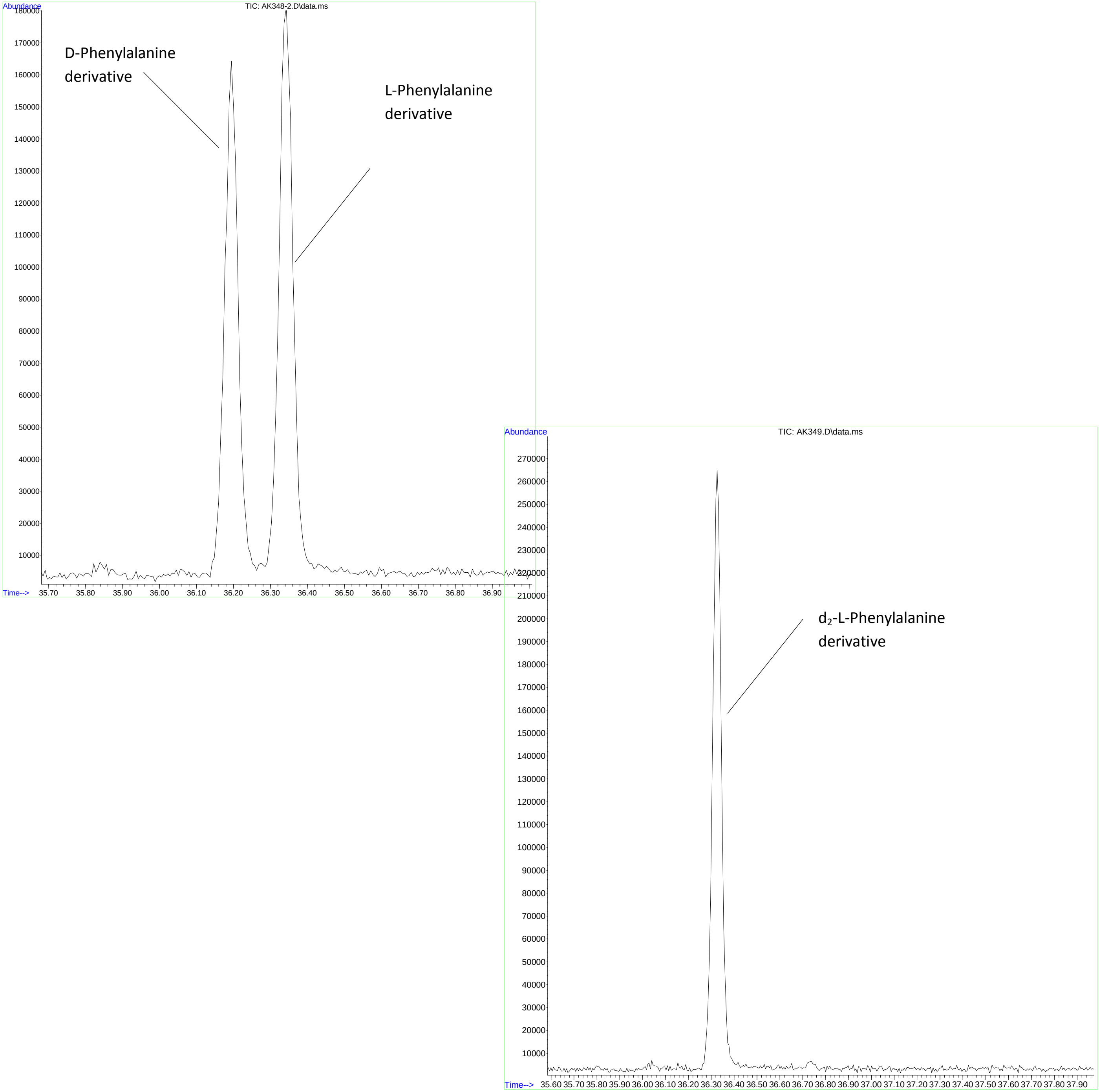

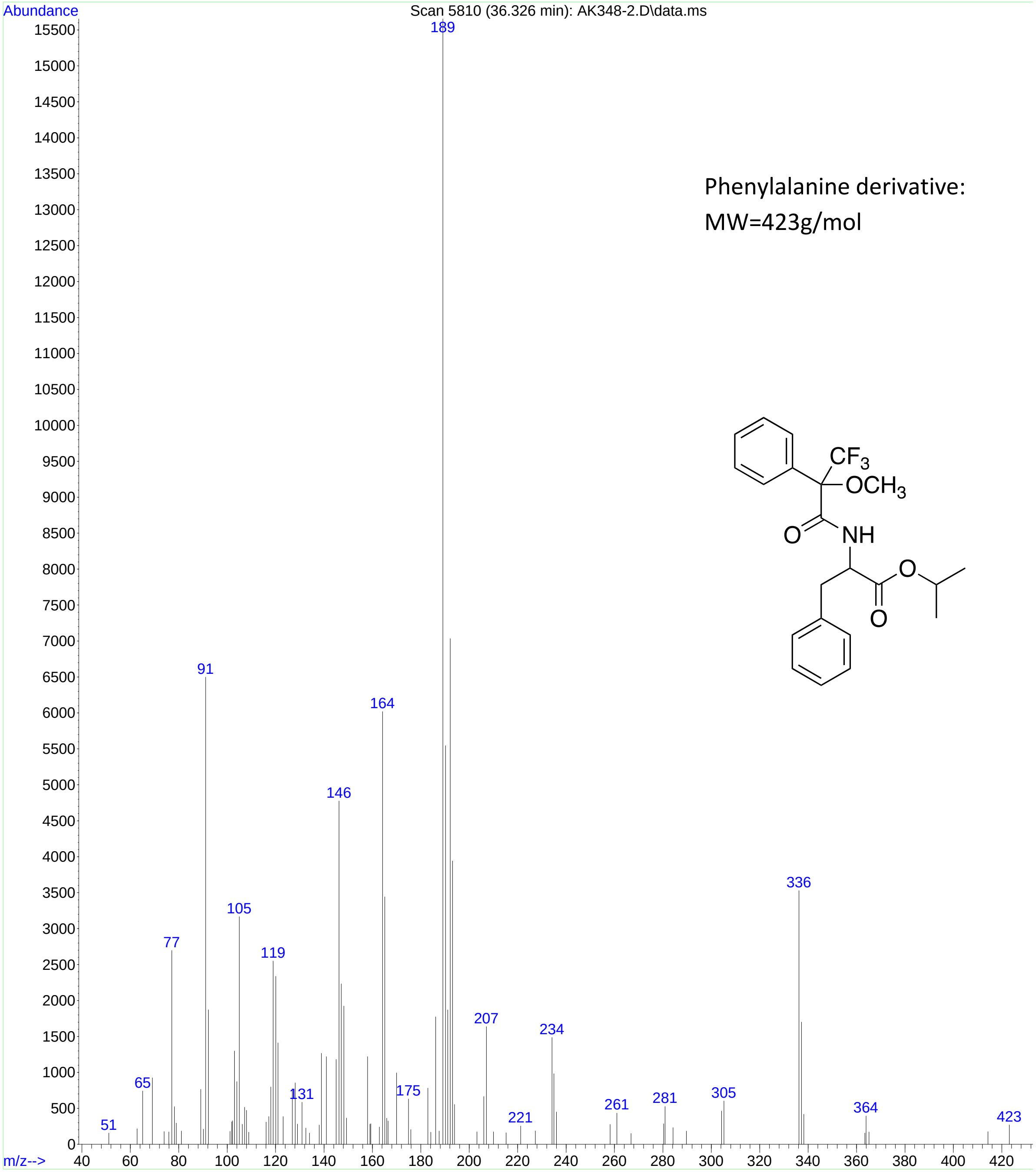
Abundance
Scan 5810 (36.326 min): AK348-2.D\data.ms
Phenylalanine derivative:
MW=423g/mol
CF3
OCH3
O
NH
O
O
m/z-->
189
91
164
146
105
119
77
336
207
234
65
131
175
51
221
261
281
305
364
423

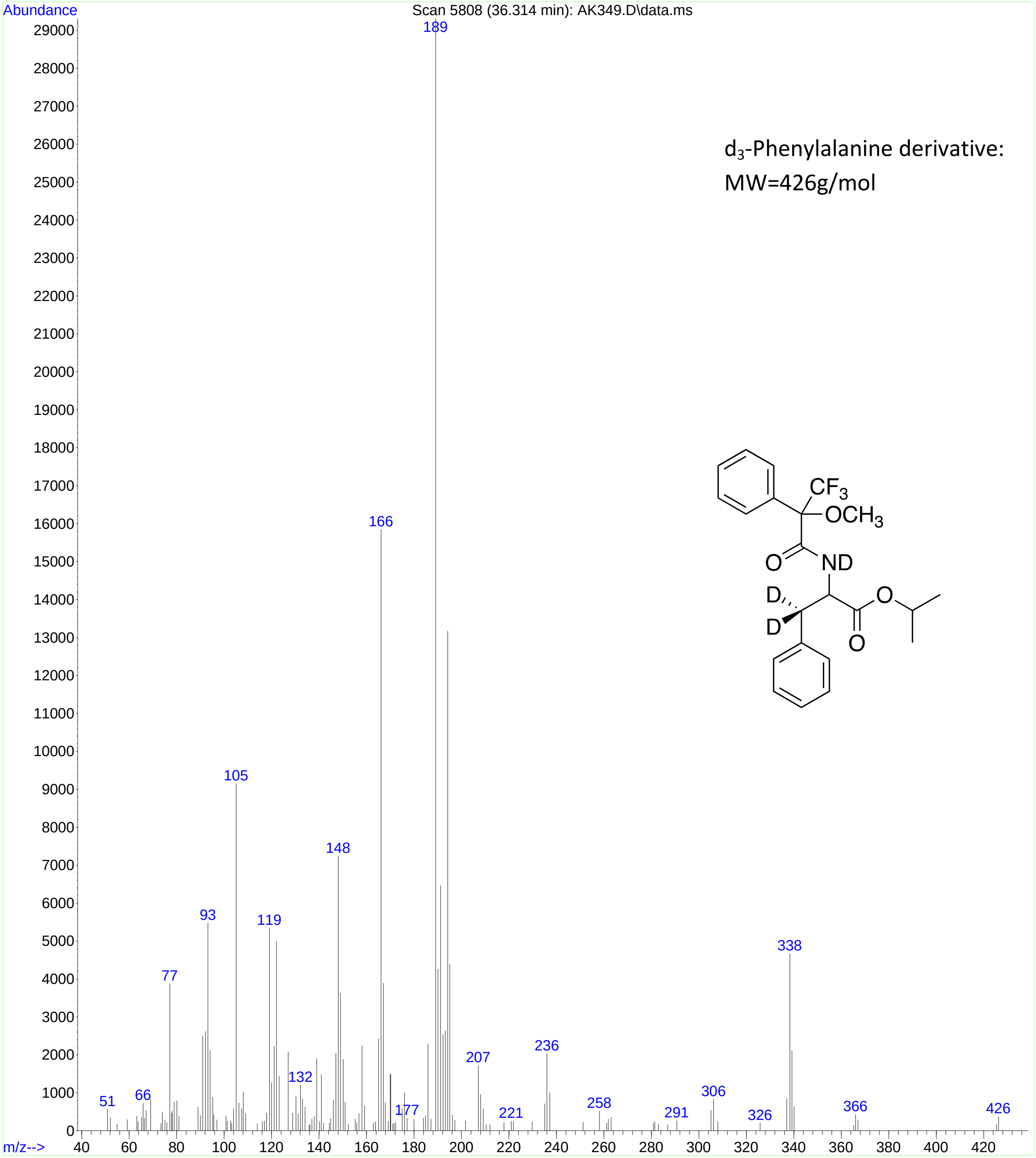
Abundance
Scan 5808 (36.314 min): AK349.D\data.ms
m/z-->
d$_3$-Phenylalanine derivative:
MW=426g/mol
CF3
OCH3
O
ND
D
D
O
O
189
166
105
148
93
119
77
338
236
207
132
306
51
66
258
177
221
291
326
366
426

Figure S12. H-D exchange reaction of glycine

(20mg of Pd/C, MW, 170℃, 60min))

a. ^{1}H NMR

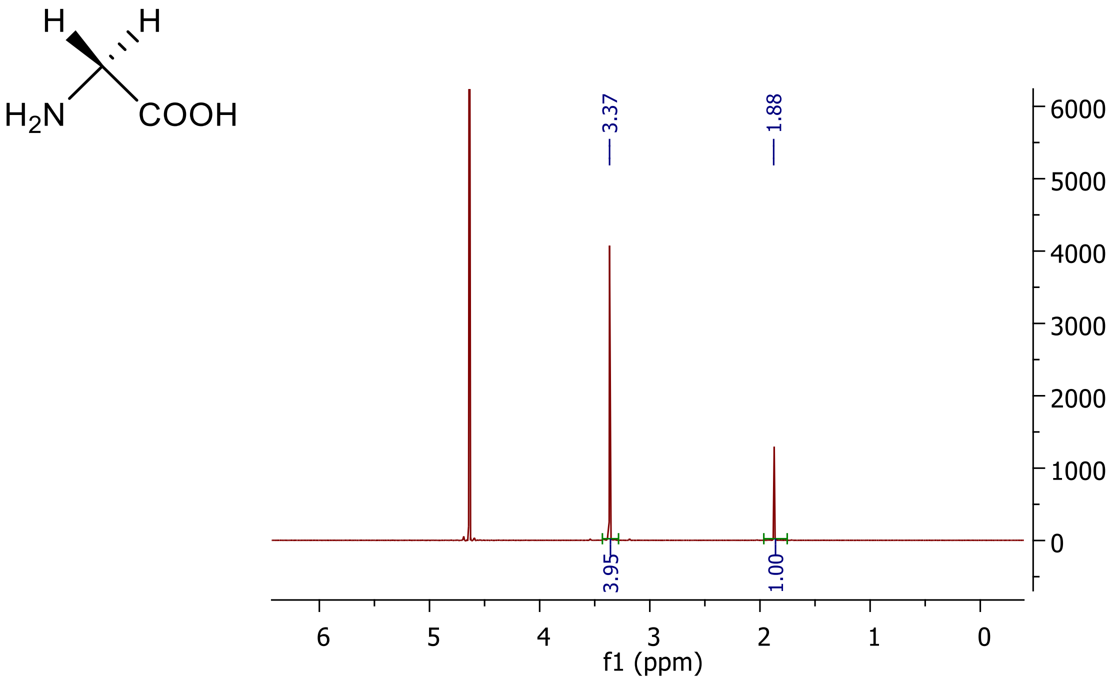

100mg Al

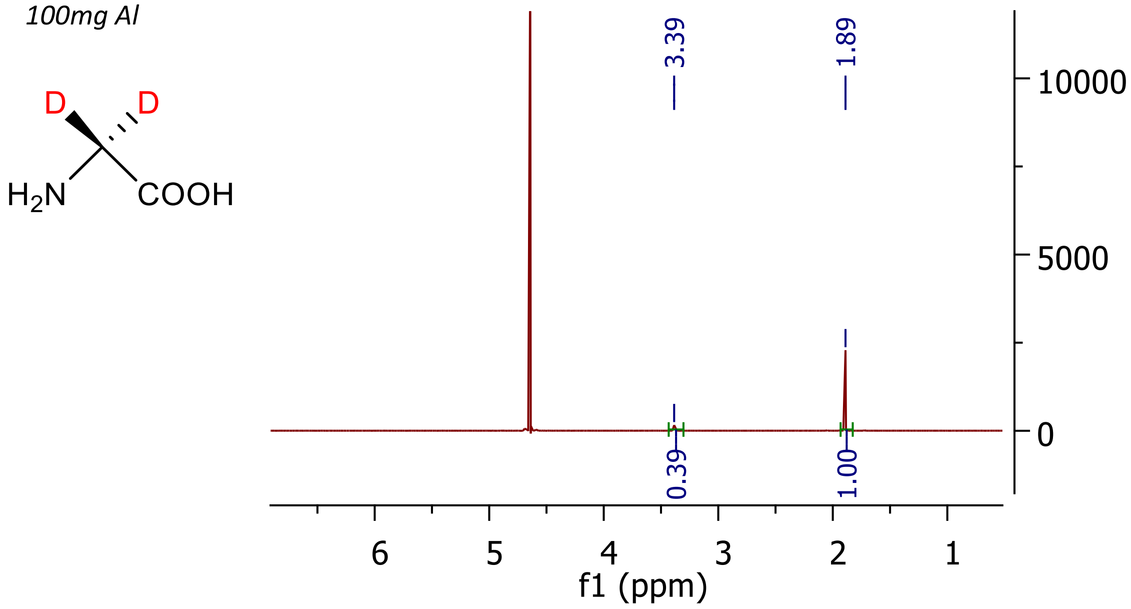

25mg Al

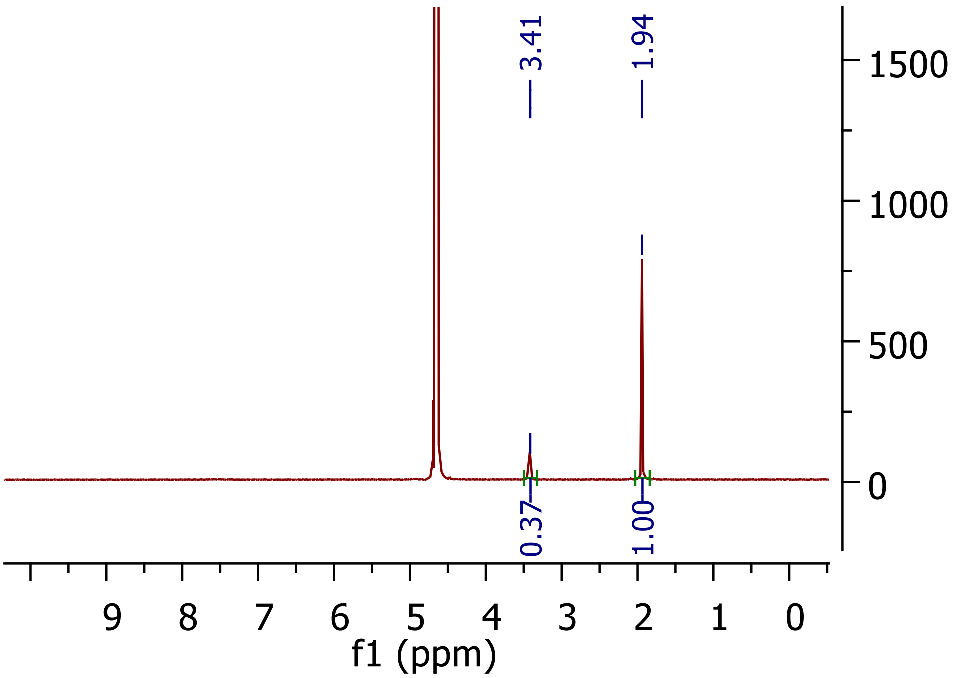

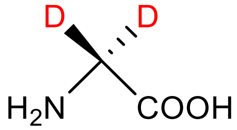

b. ^{2}H NMR

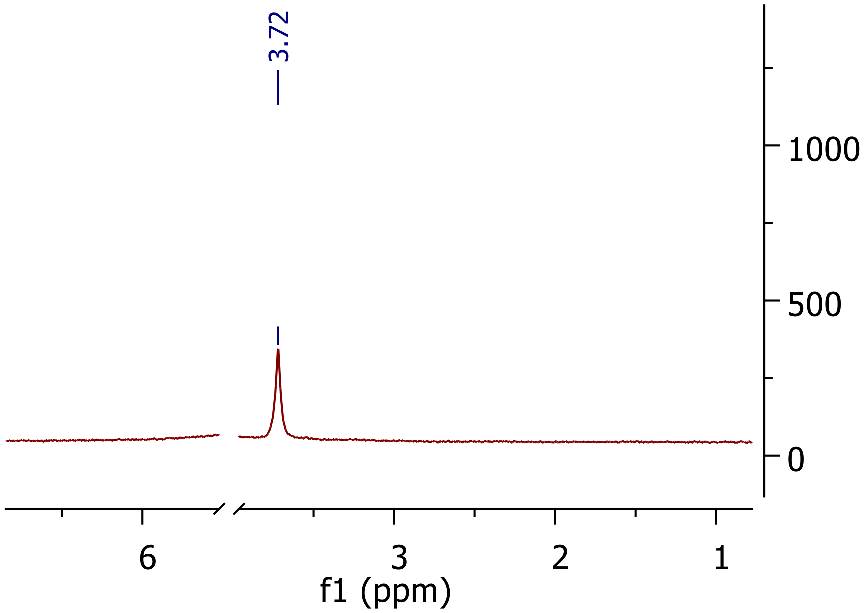

c. **Mass Spectrum**

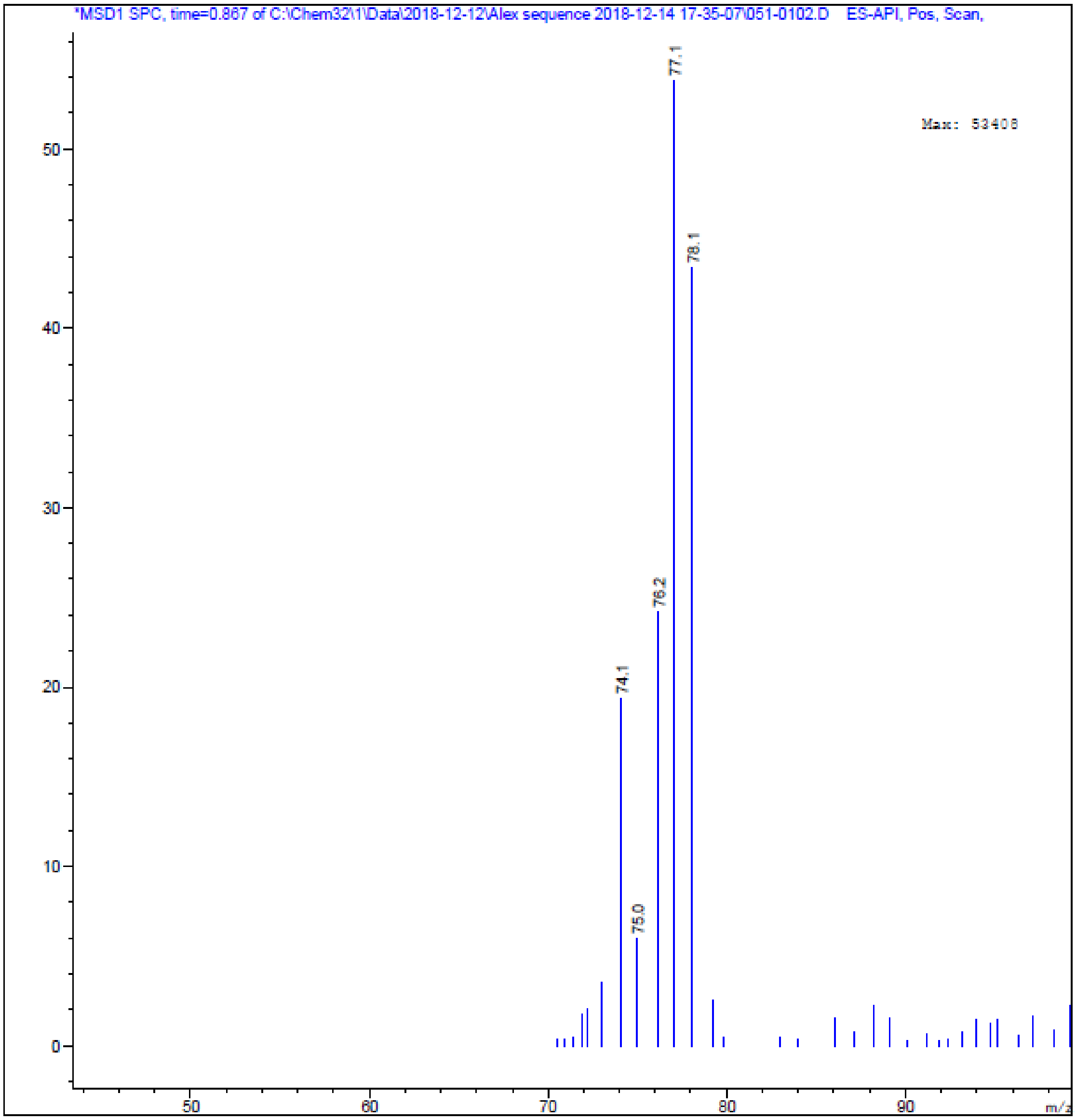

/CMS 12/14/2018 6:52:25 PM SYSTEM Page 1 of 1

Figure S13. H-D exchange reaction of histidine

(20mg of Pd/C, MW, 120℃, 60min)

a. ^{1}H NMR

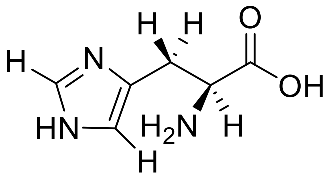

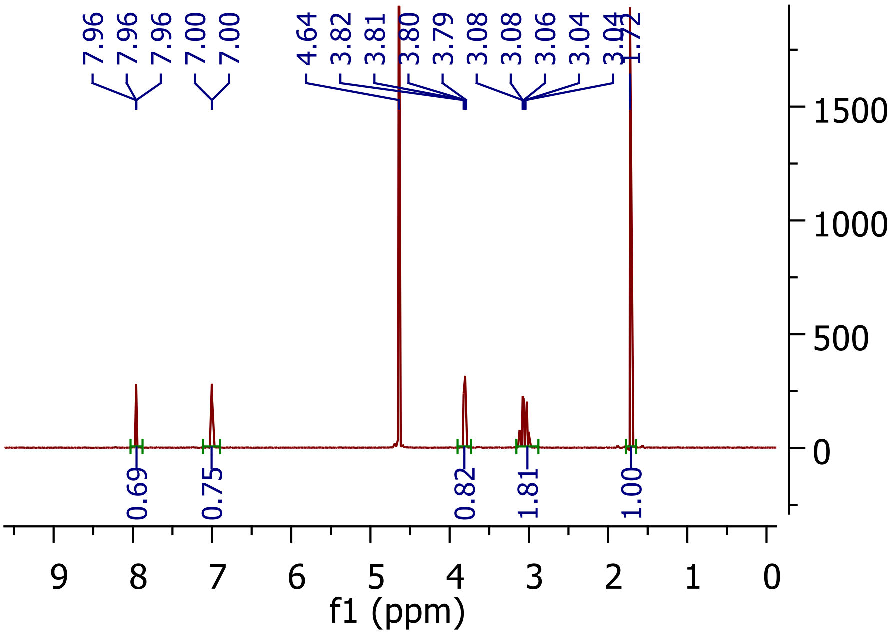

100mg Al

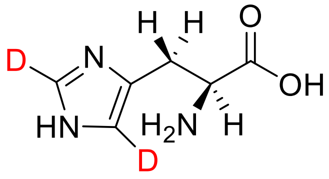

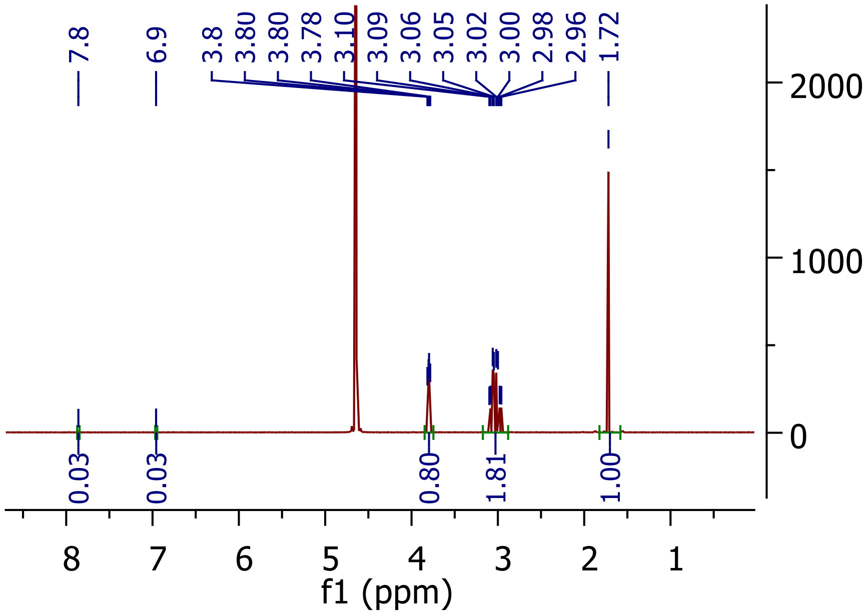

25mg Al

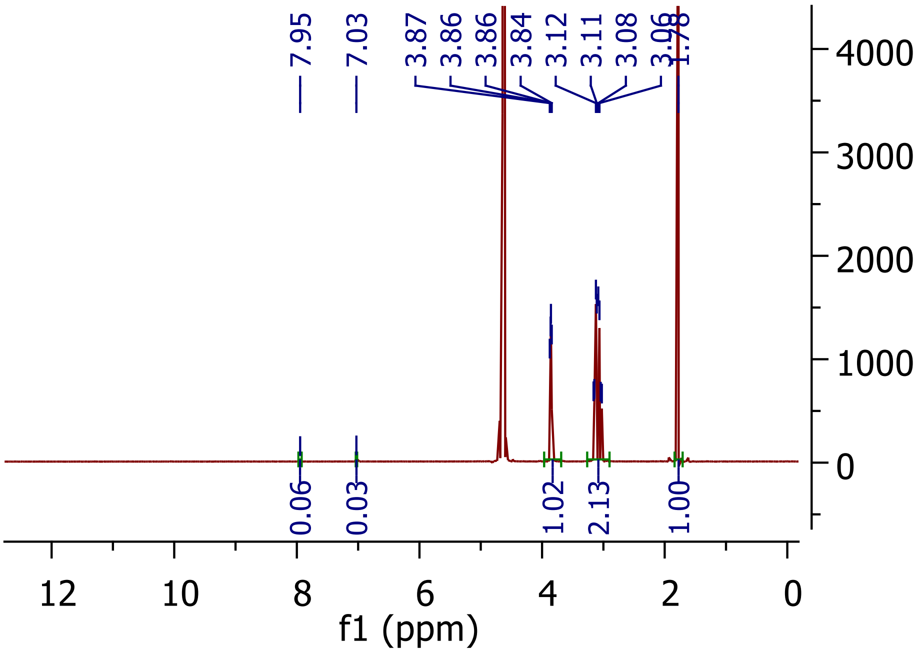

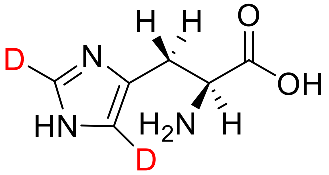

b. **^{2}H NMR**

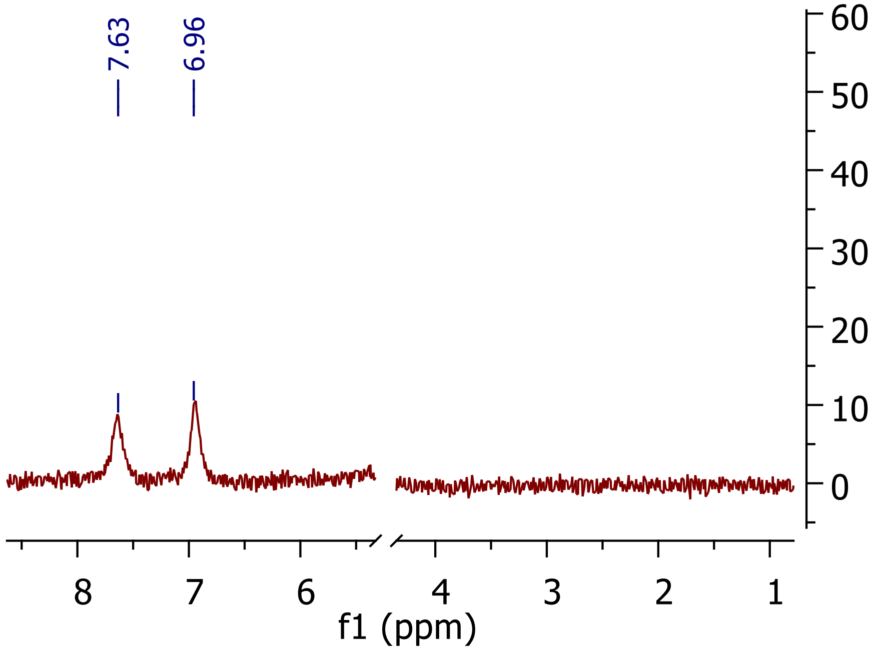

c. Mass spectrum

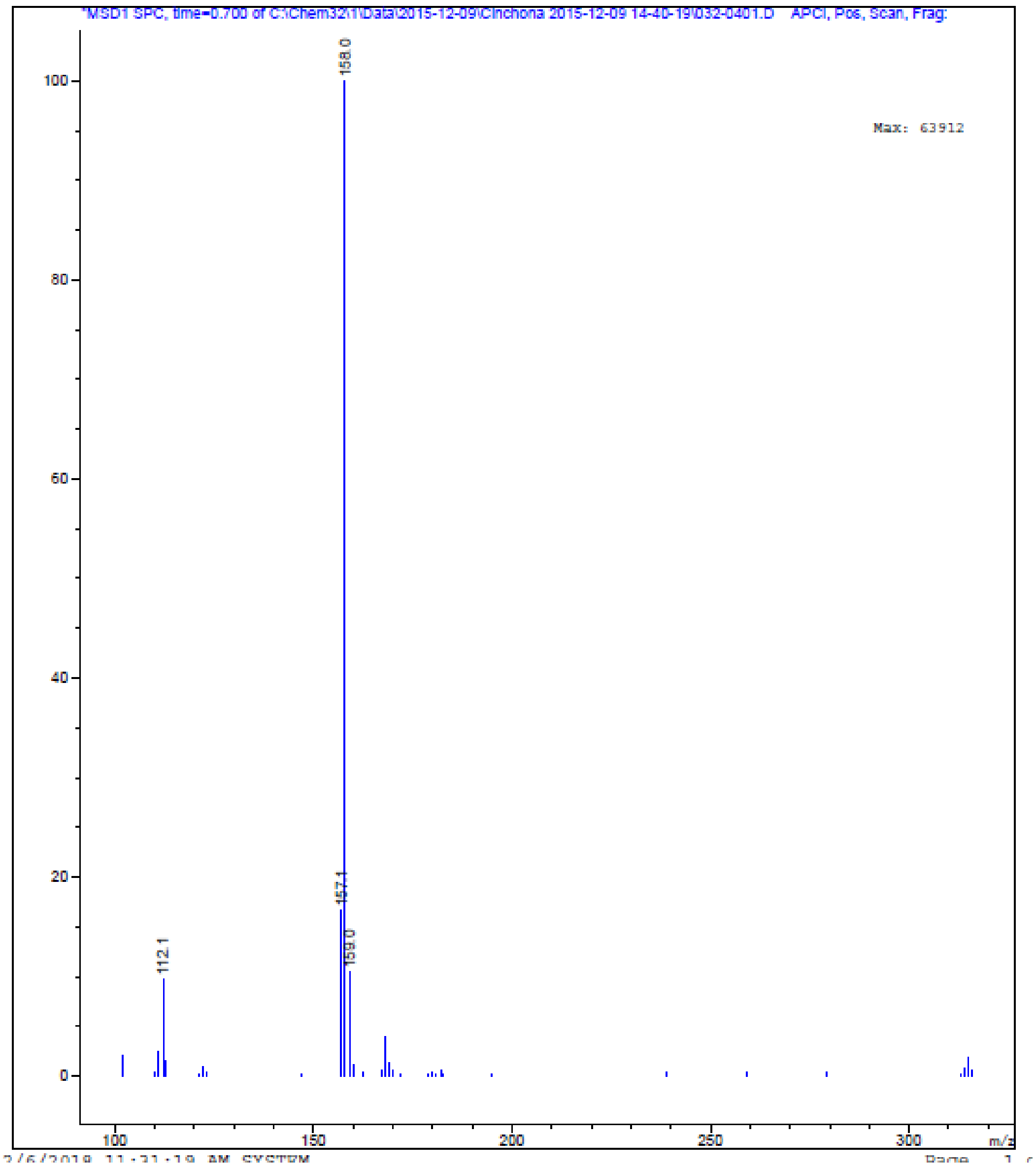

Figure S14. H-D exchange reaction of alanine

(20mg of Pd/C, MW, 120℃, 60min)

a. ^{1}H NMR

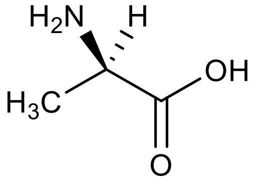

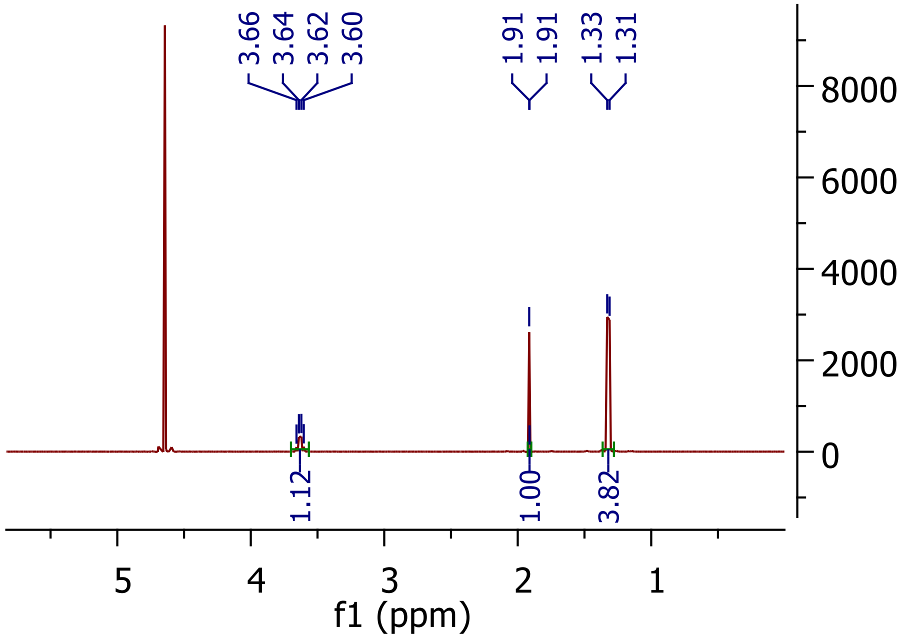

100mg Al

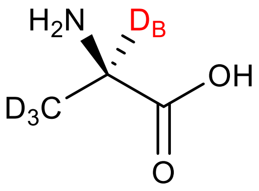

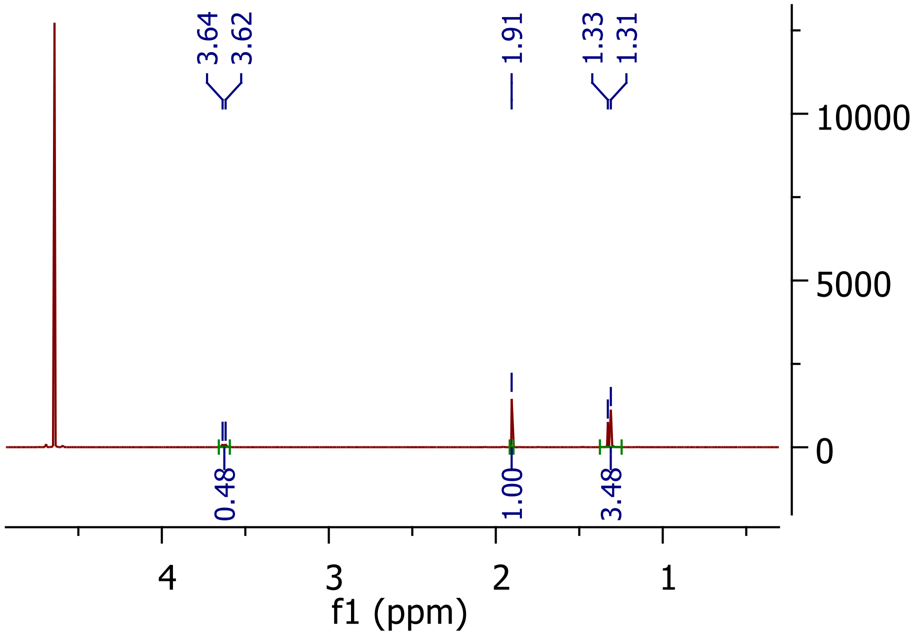

25mg Al (40% yield)

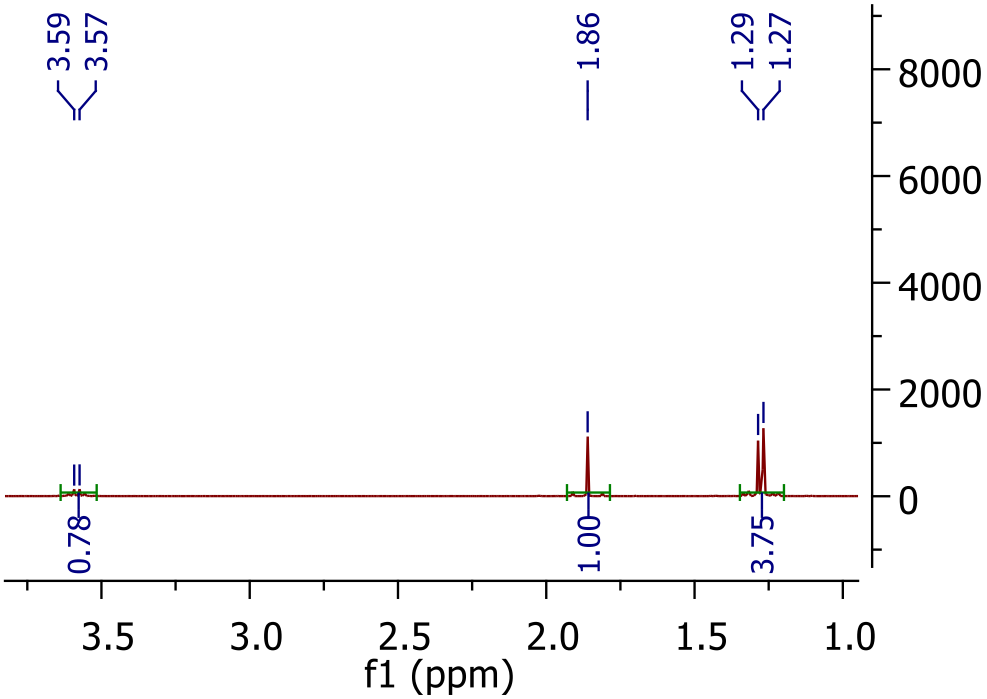

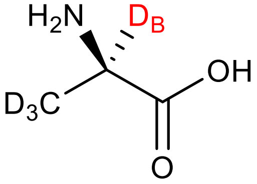

b. ^{2}H NMR

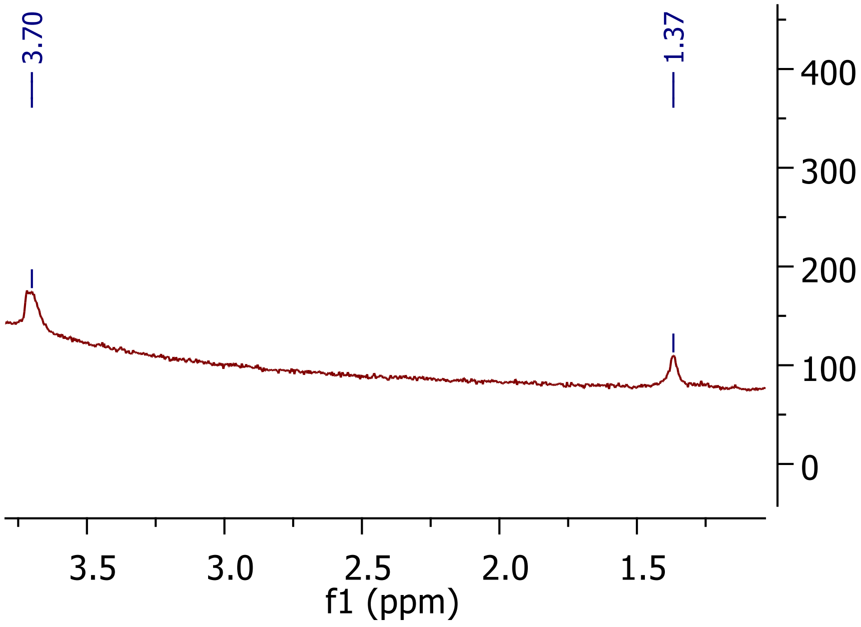

c. Mass spectrum

d_1-L-alanine/d_4-L-alanine

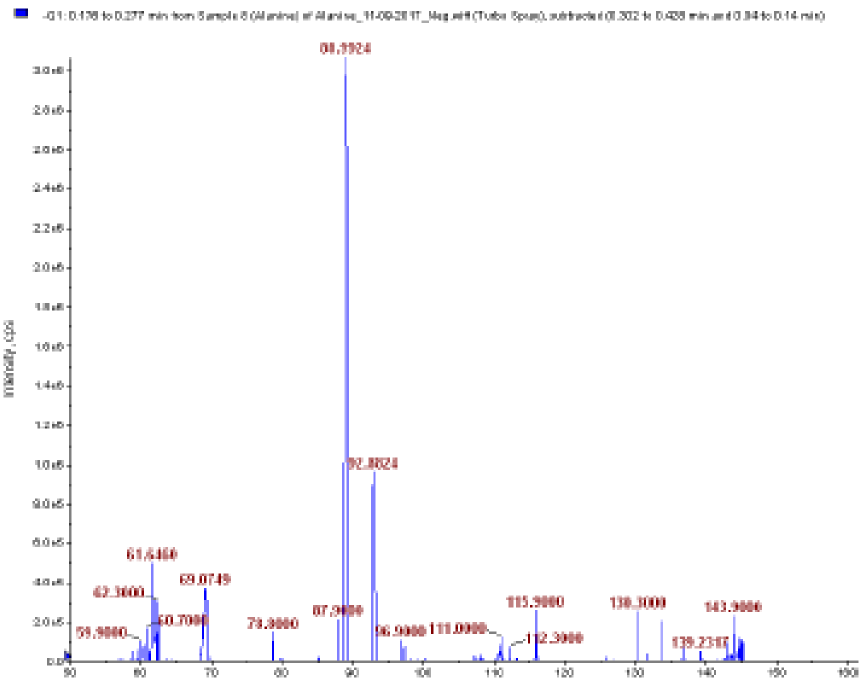

Non-deuterated L-alanine

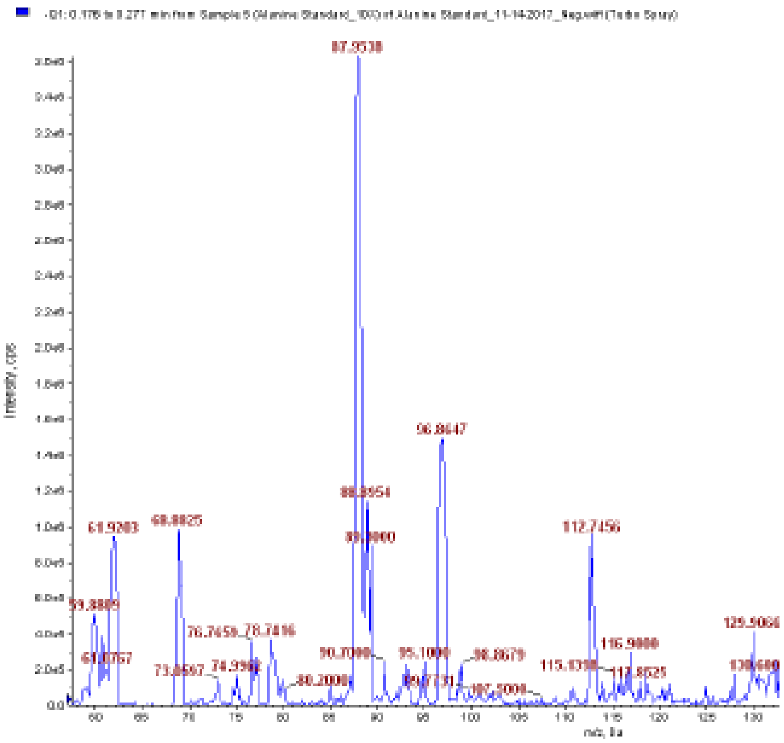

Figure S15. Determination of the chirality of deuterated alanine

Alanine was derivatized using isopropyl alcohol to form an amino acid ester first and then it was reacted with Mosher's chloride to functionalize the amino group; before it was injected in the GC-MS. The first chromatogram shows the separation of a pseudo racemic mixture of alanine. The mass spectrum of both products was identical. The second chromatogram corresponds to the derivatized d_2-alanine.

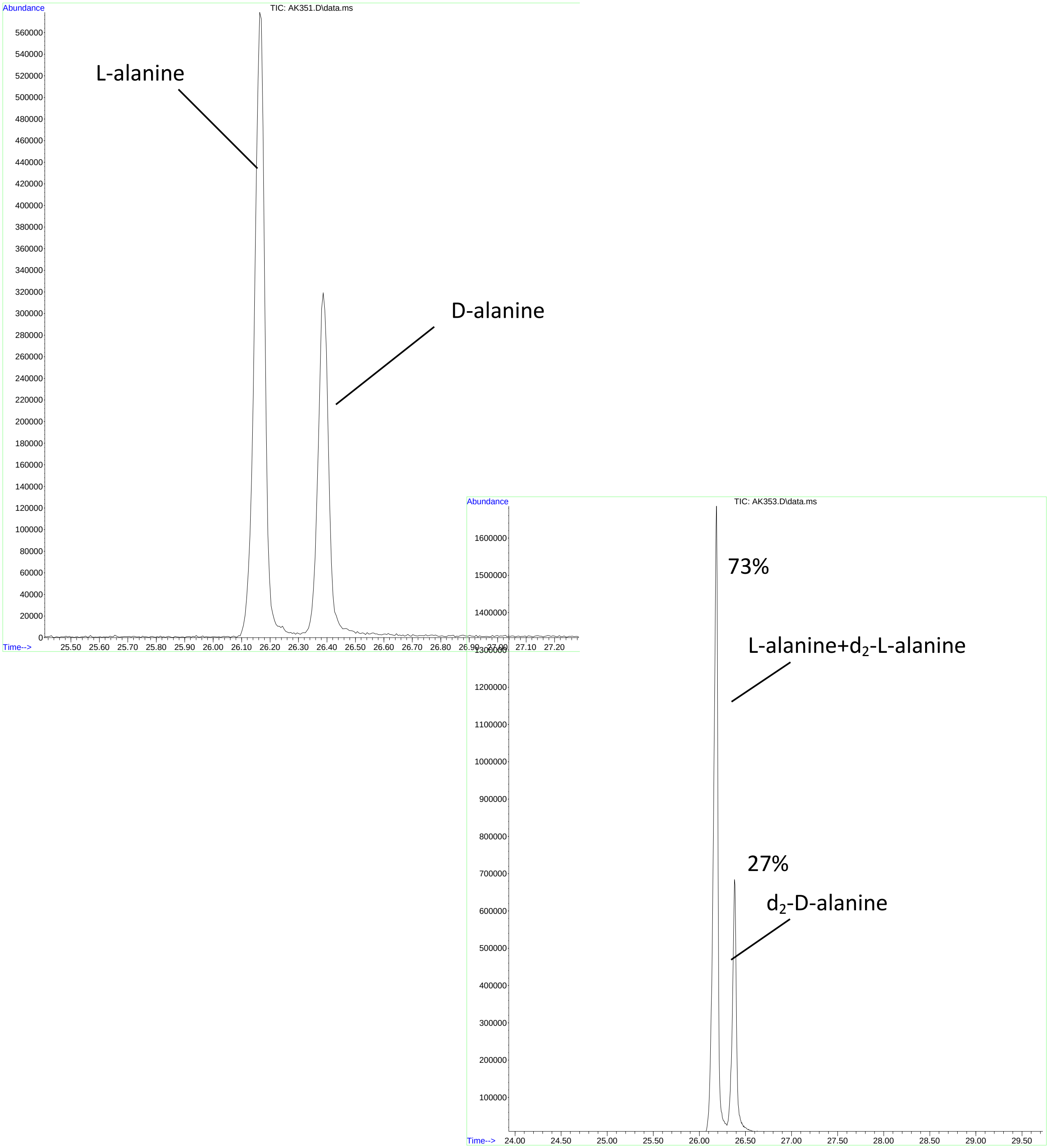

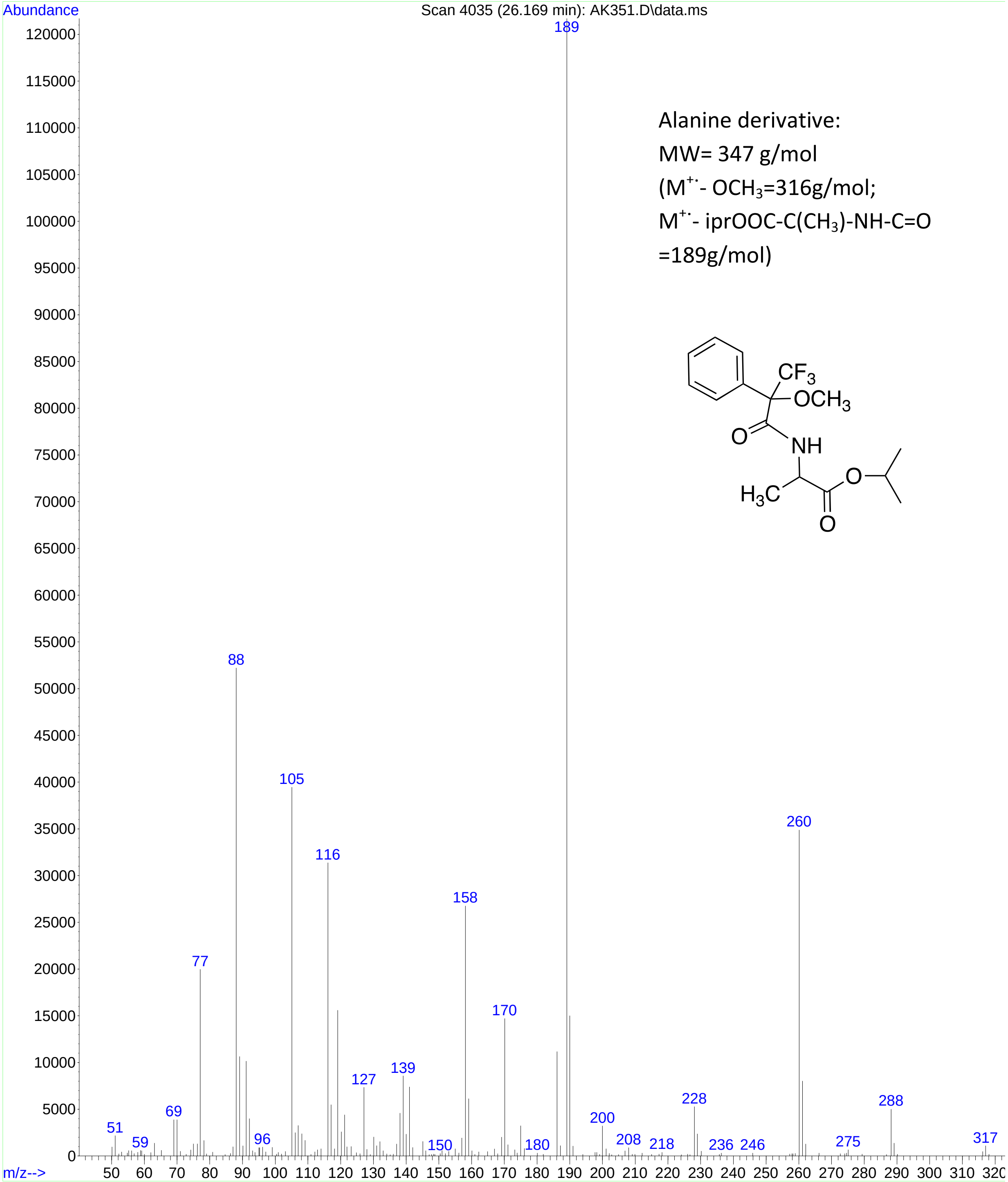
Abundance
Scan 4035 (26.169 min): AK351.D\data.ms
m/z-->
189
88
105
116
158
260
77
170
139
127
228
288
69
200
51
59
96
150
180
208
218
236
246
275
317
Alanine derivative:
MW= 347 g/mol
(M+·- OCH3=316g/mol;
M+·- iprOOC-C(CH3)-NH-C=O
=189g/mol)
CF3
OCH3
O
NH
H3C
O
O

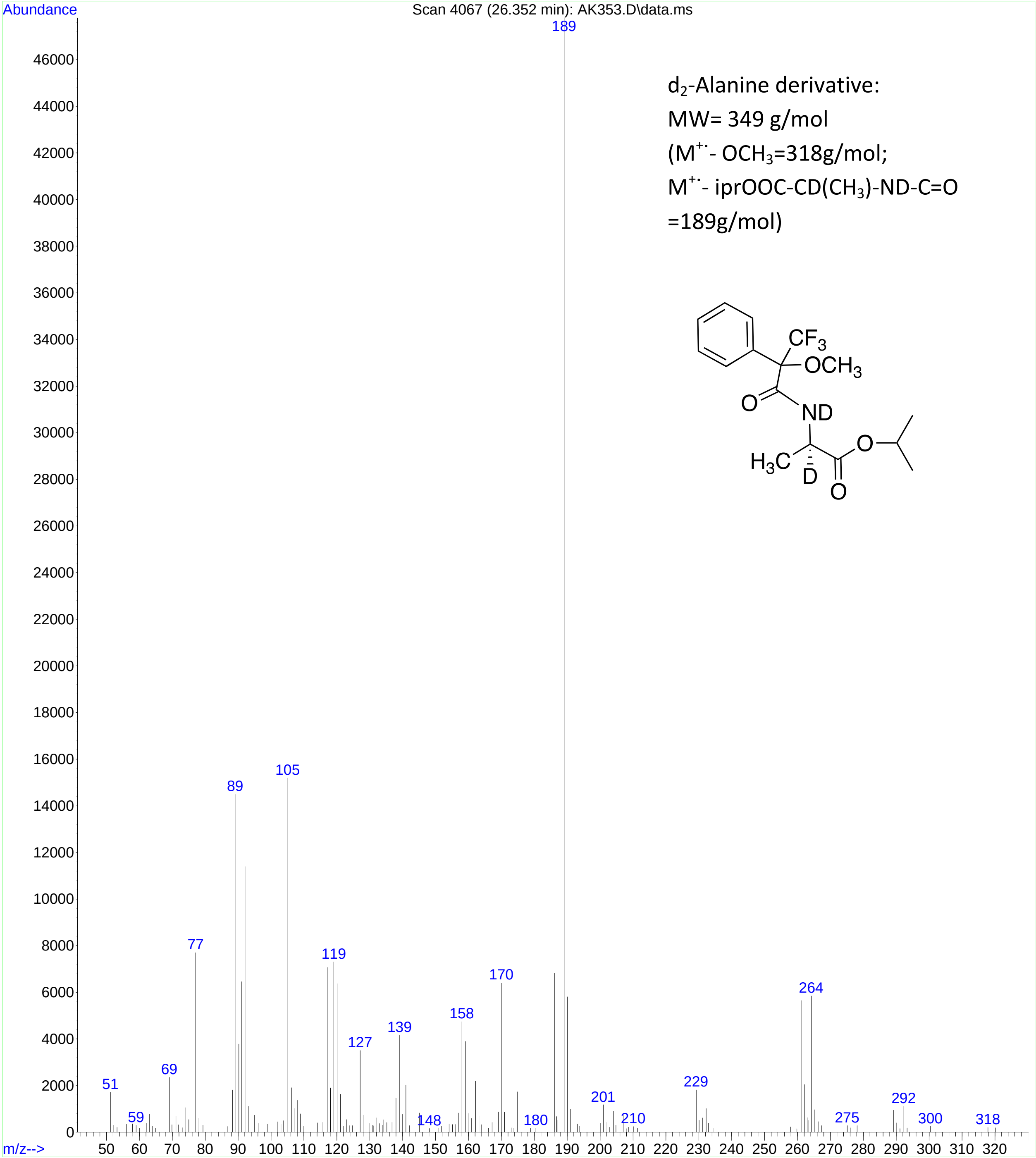
Abundance
Scan 4067 (26.352 min): AK353.D\data.ms
m/z-->
189
105
89
77
119
170
264
127
139
158
51
59
69
148
180
201
210
229
275
292
300
318
d2-Alanine derivative:
MW= 349 g/mol
(M+·- OCH3=318g/mol;
M+·- iprOOC-CD(CH3)-ND-C=O
=189g/mol)
CF3
OCH3
O
ND
H3C
D
O
O

Figure S16. H-D exchange reaction of ethylaniline

(20mg of Pd/C, MW, 60℃)

a. ^{1}H NMR

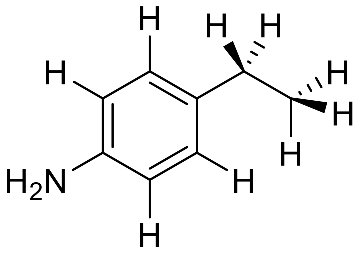

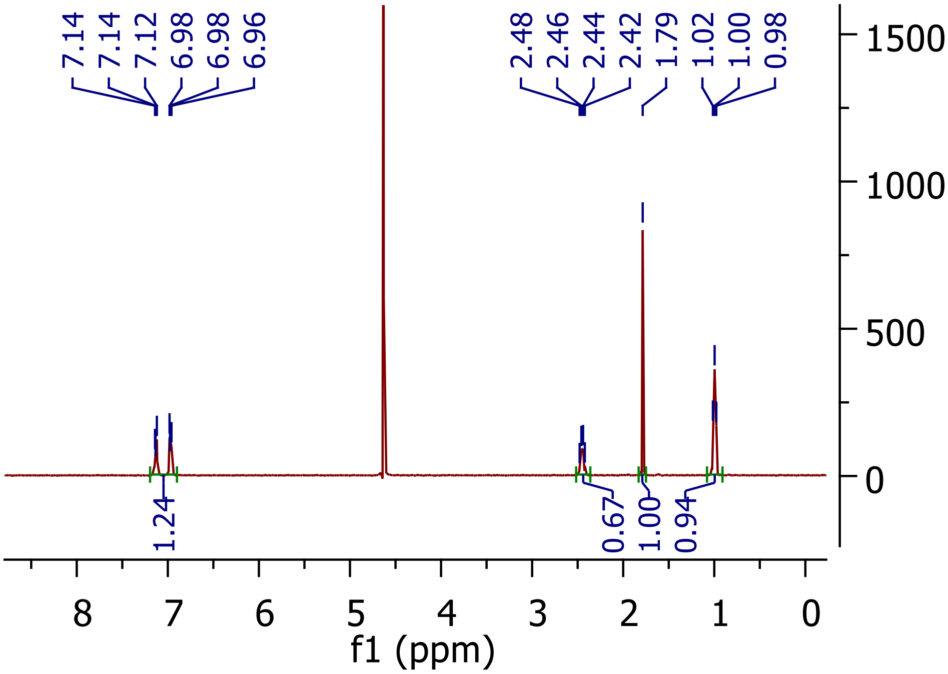

100mg Al, 60min

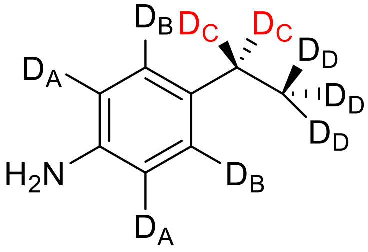

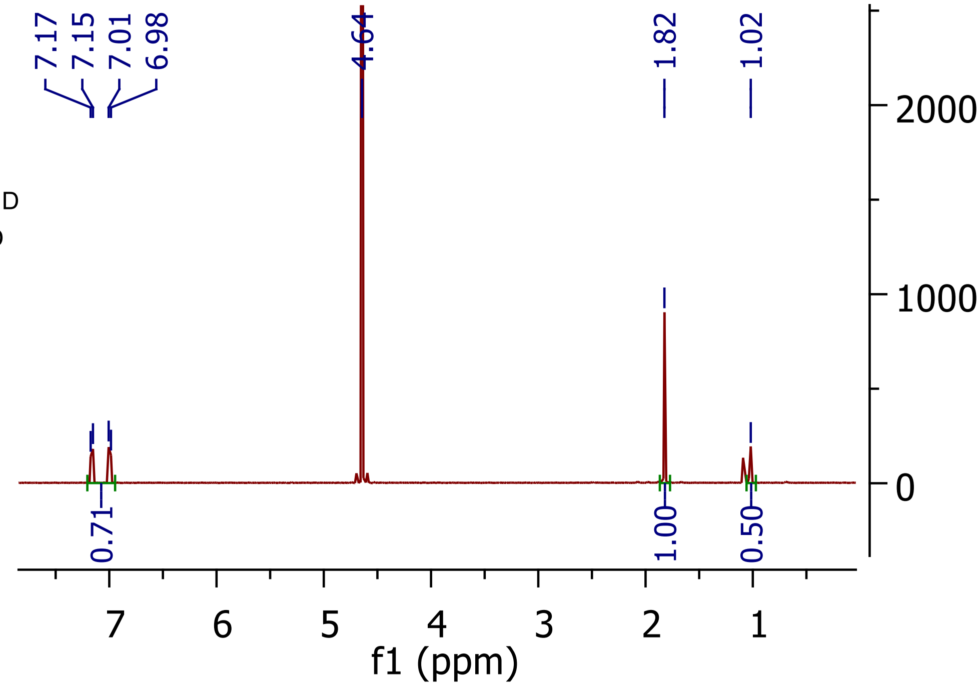

100mg Al, 20min

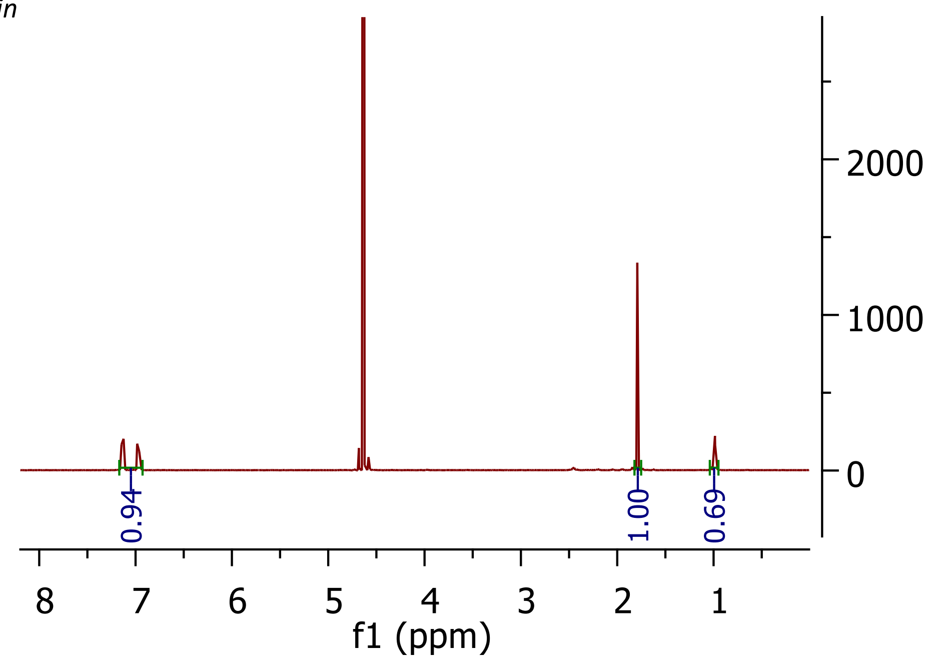

25mg Al, 20min

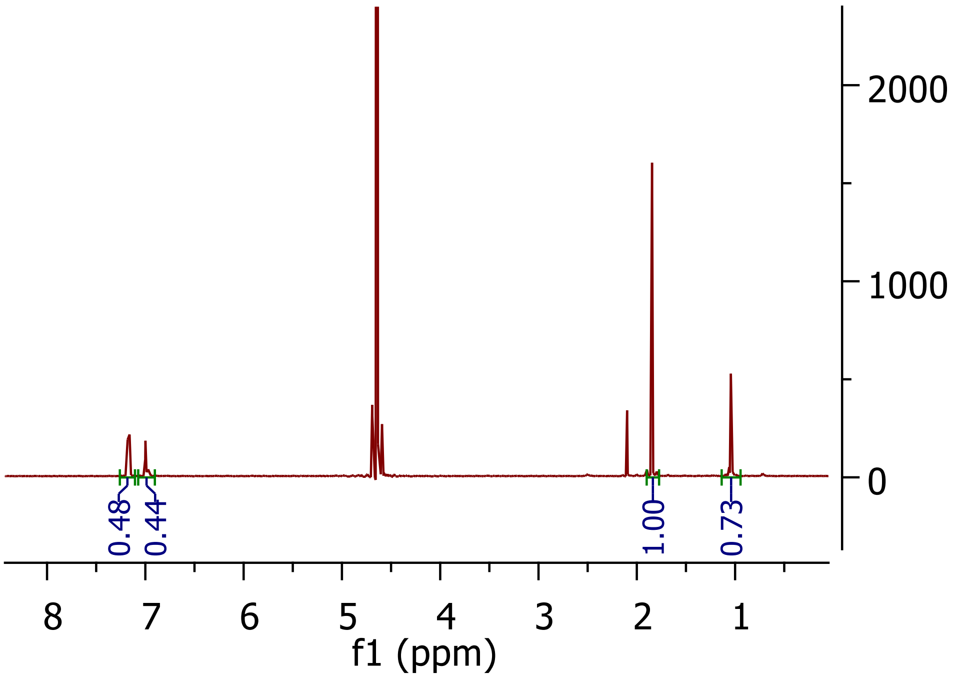

b. ***^{2}H NMR***

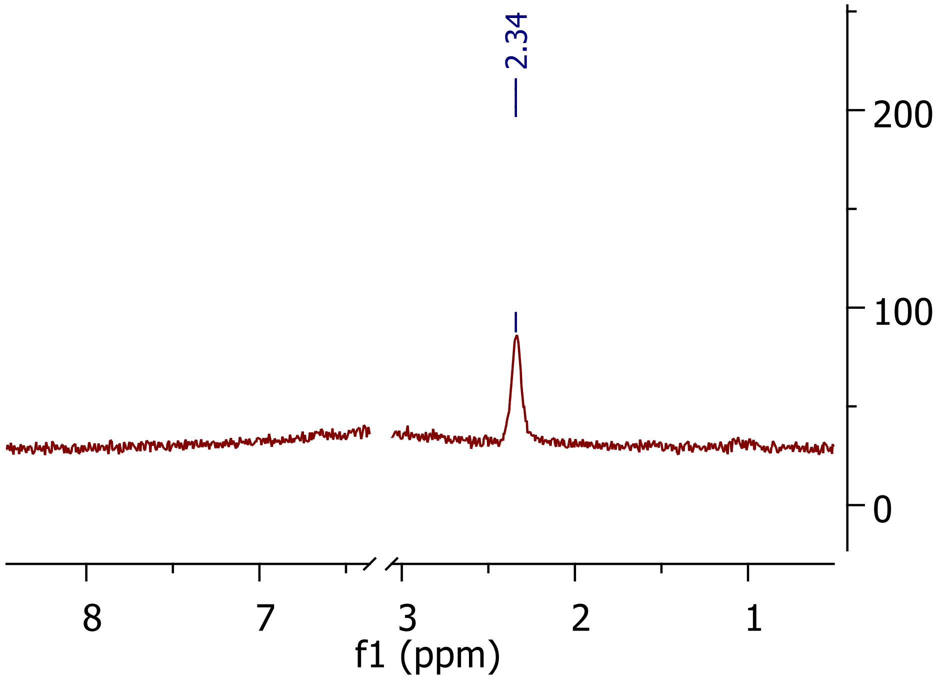

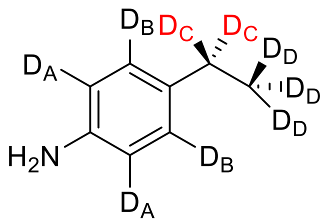

c. Mass spectrum

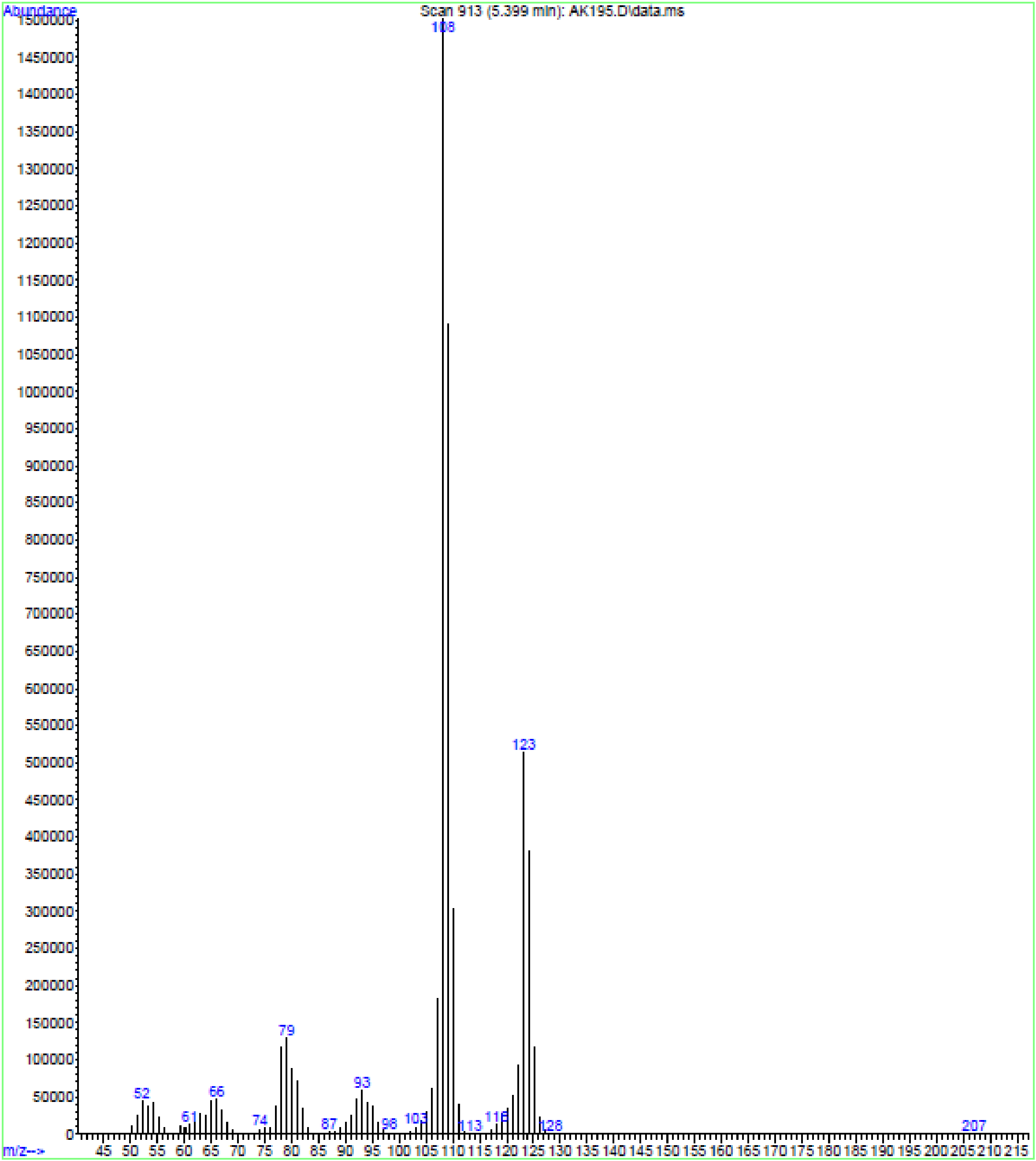

Figure S17. H-D exchange reaction of ethylenedianiline

(20mg of Pd/C, MW, 60min)

a. ^{1}H NMR

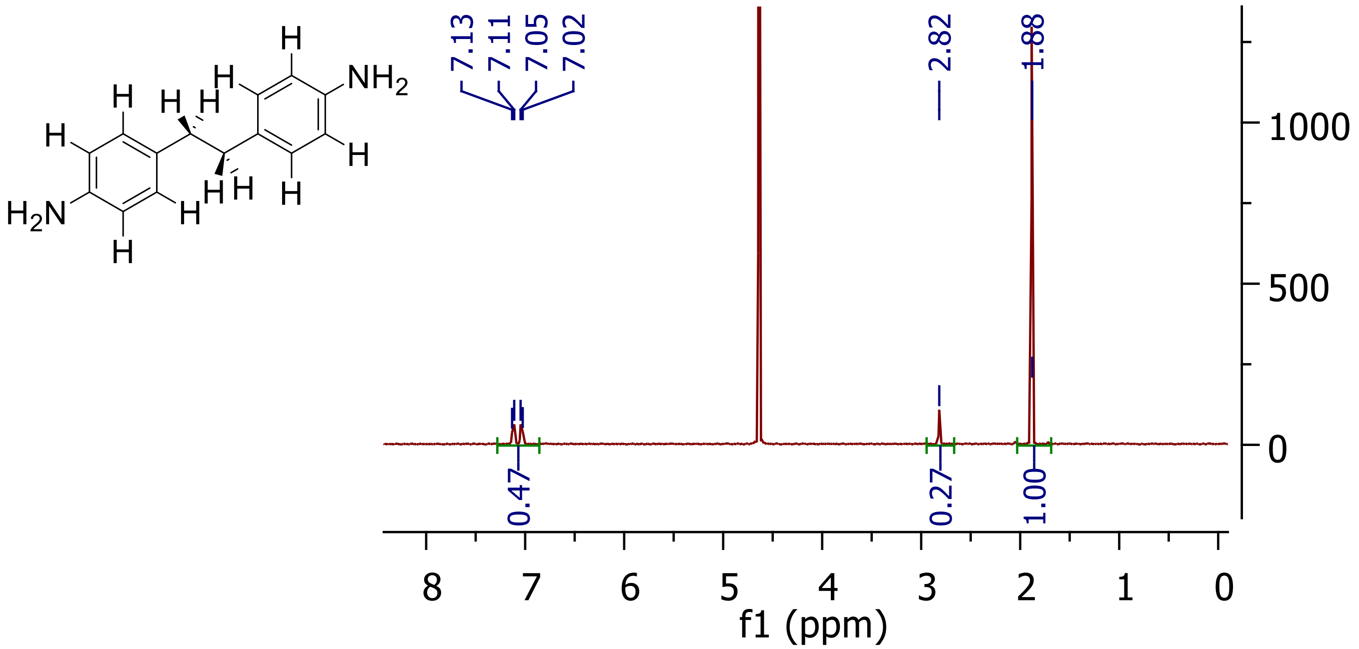

100mg Al, 80°C

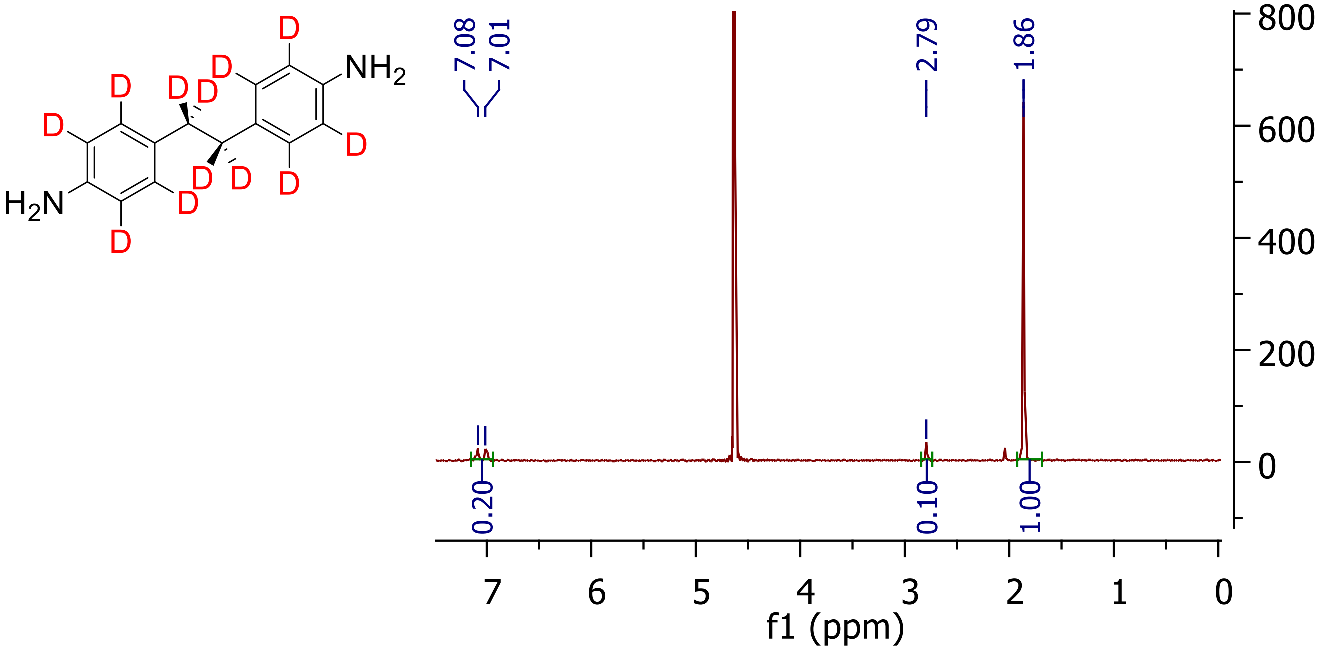

100mg of Al, 120 °C

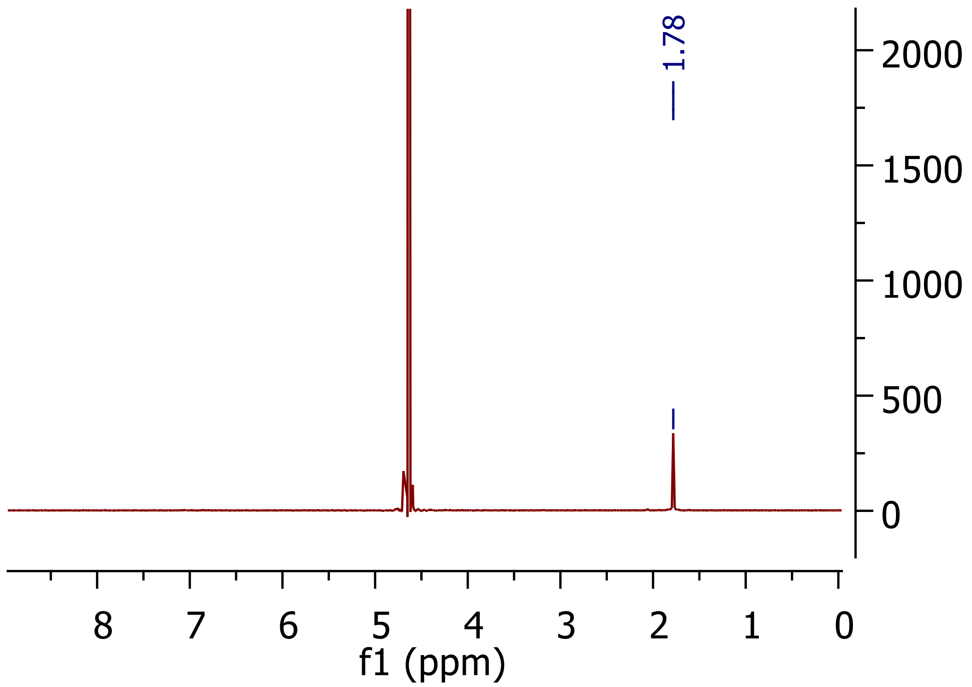

25mg Al, 120℃

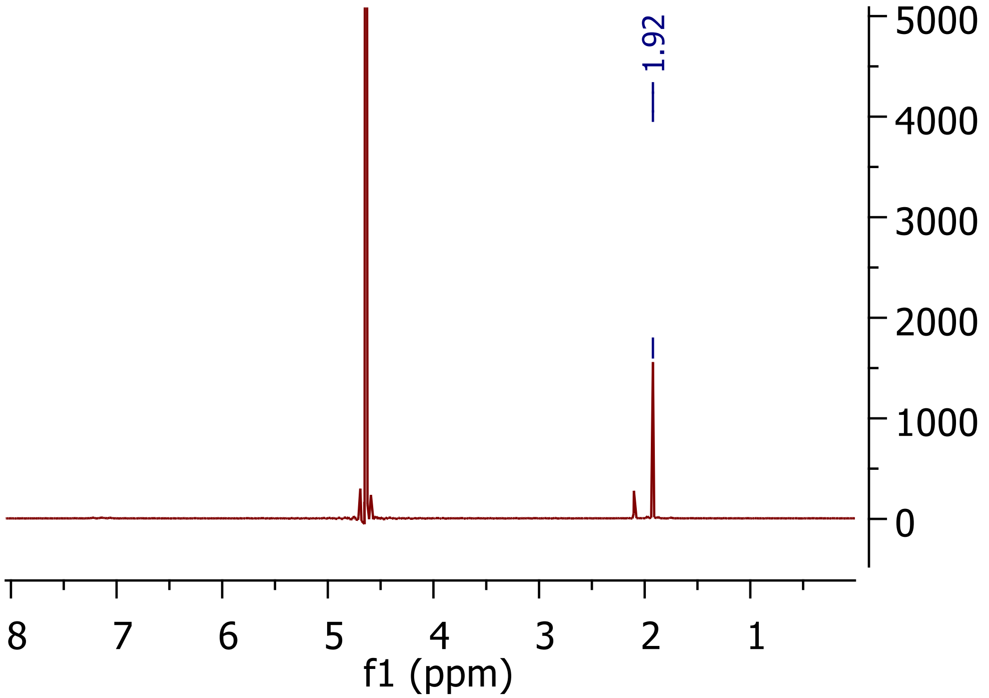

b. ^{2}H NMR

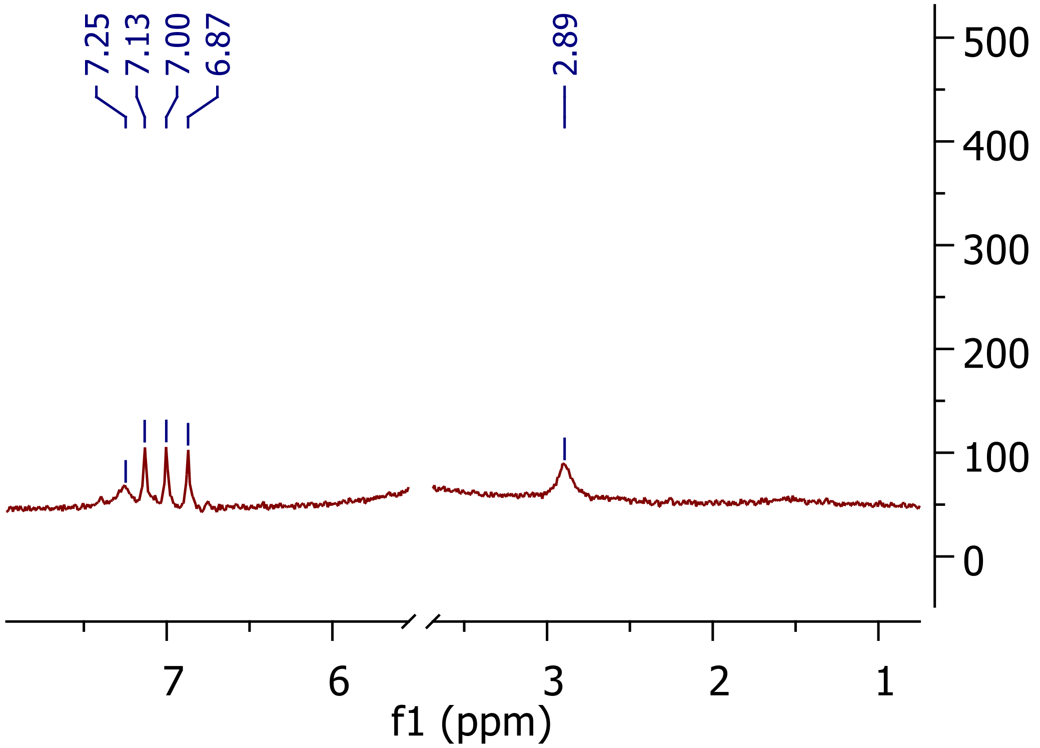

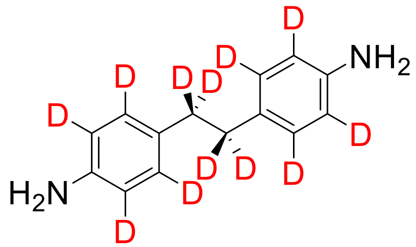

c. **Mass spectrum**

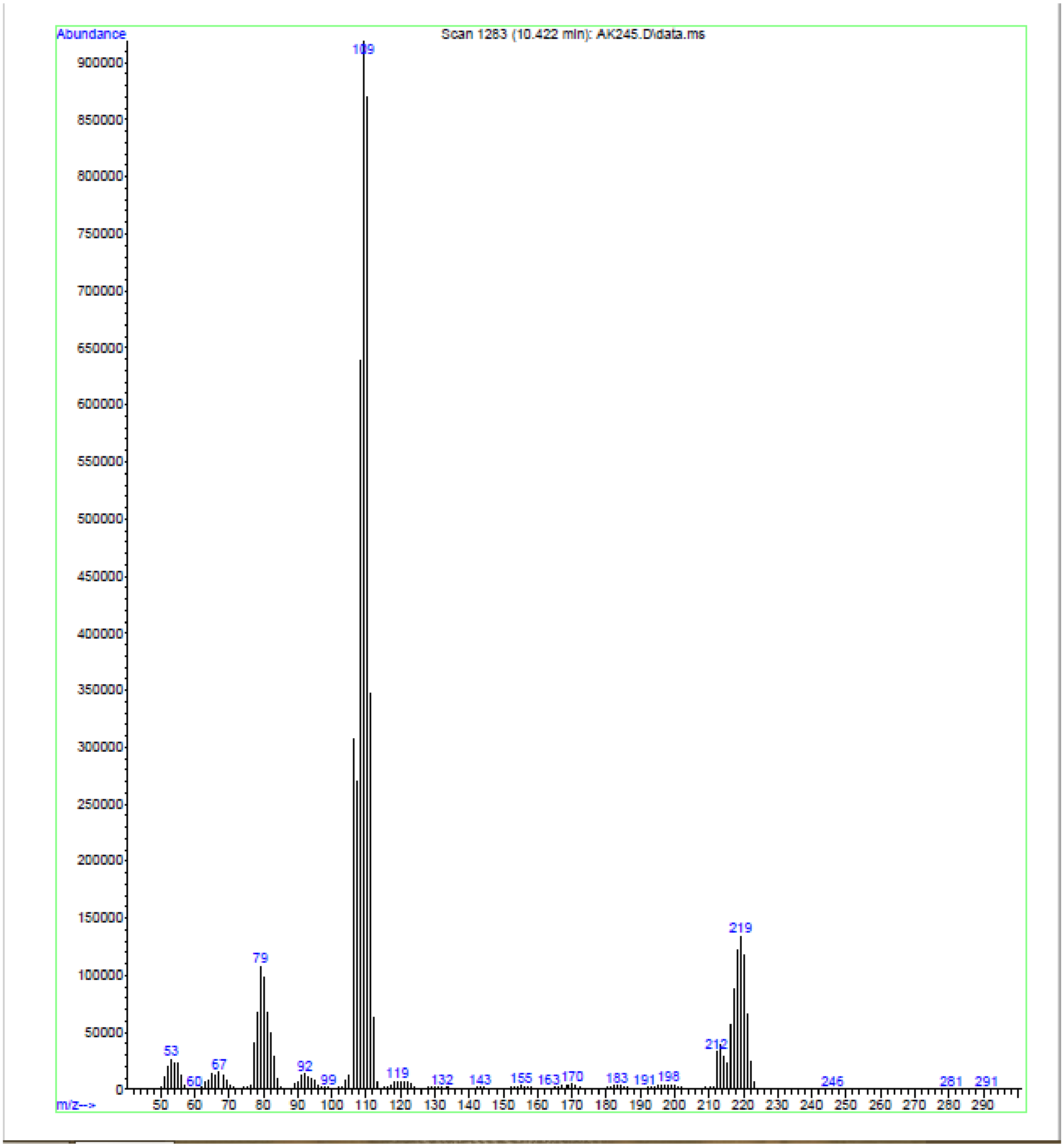

Figure S18. H-D exchange reaction of 2-phenylethylamine

(20mg of Pd/C, MW, 60min, 80°C*)*

a. ^{1}H NMR

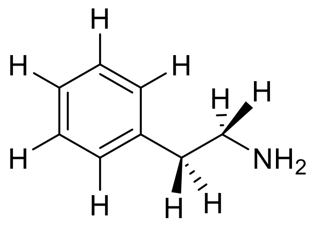

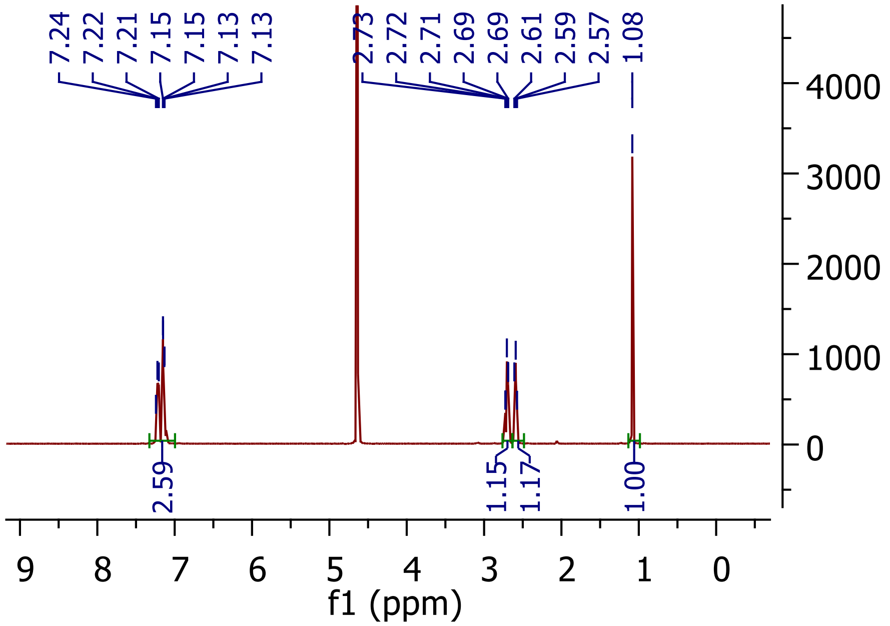

100mg Al

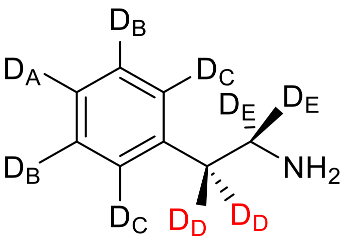

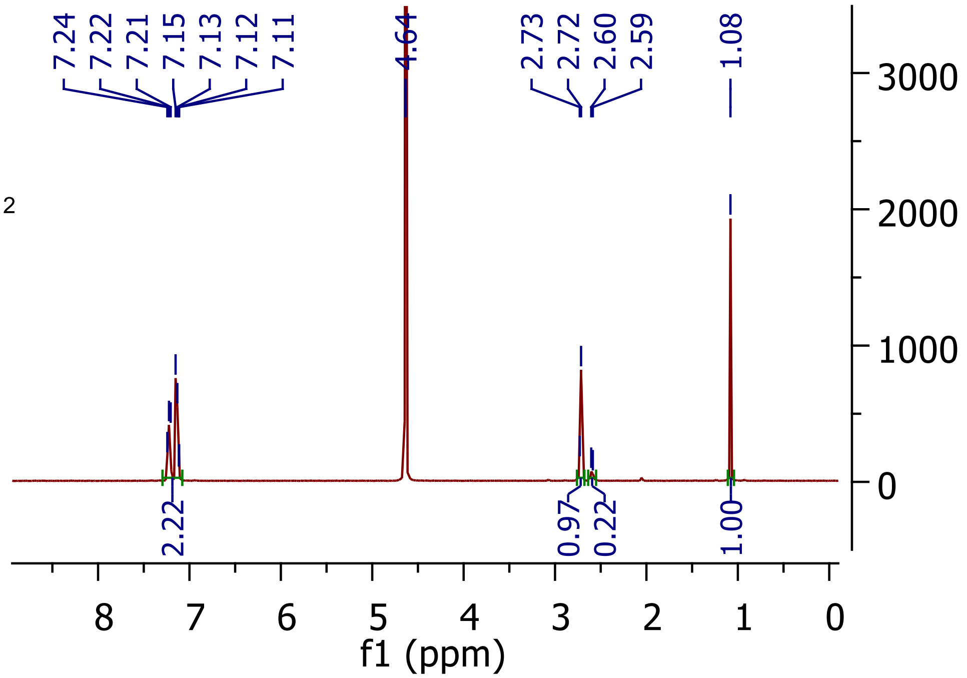

25mg Al

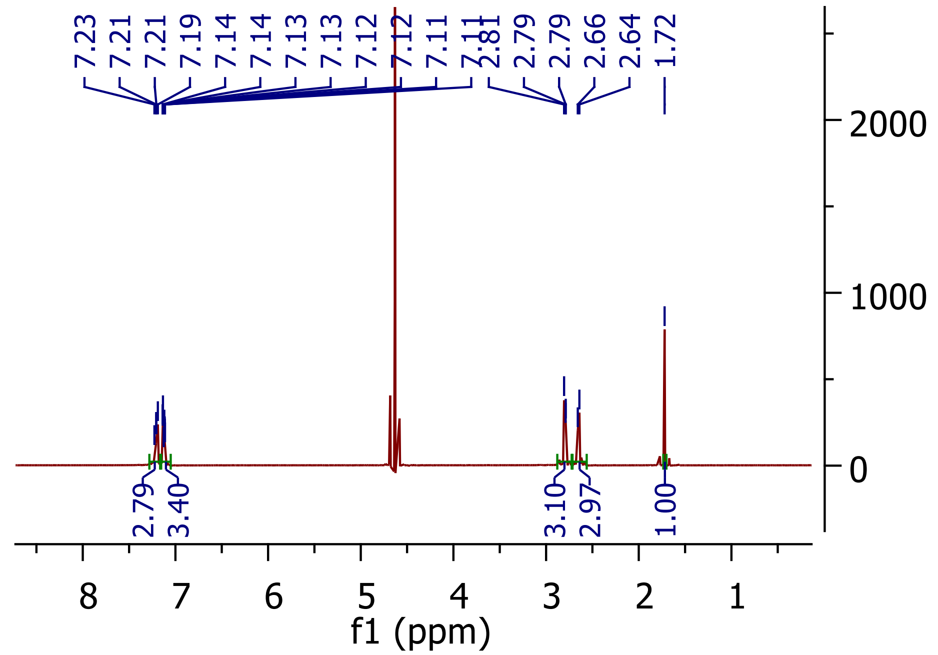

Reference

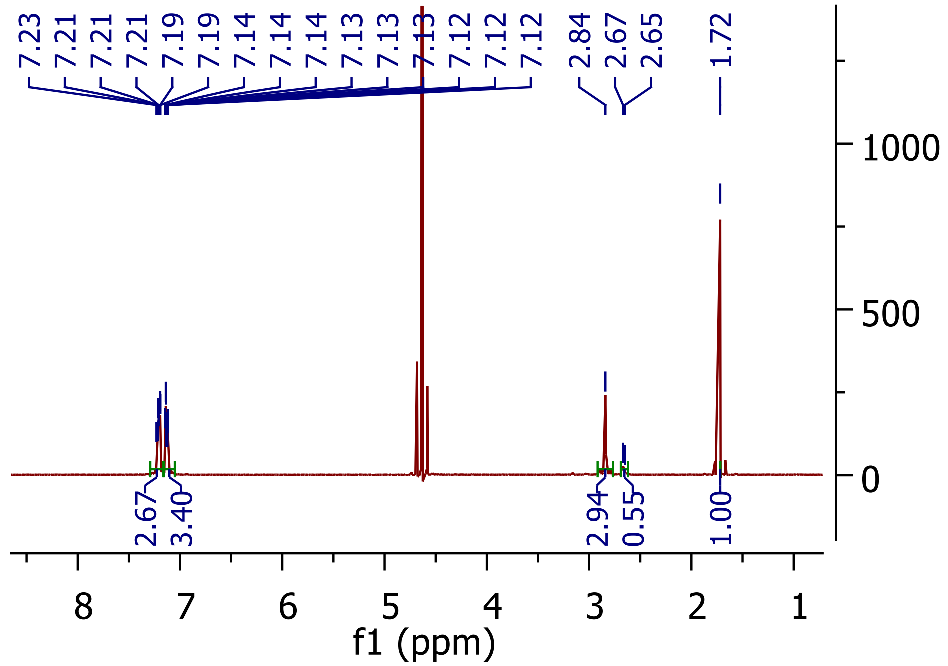

b. ^{2}H NMR

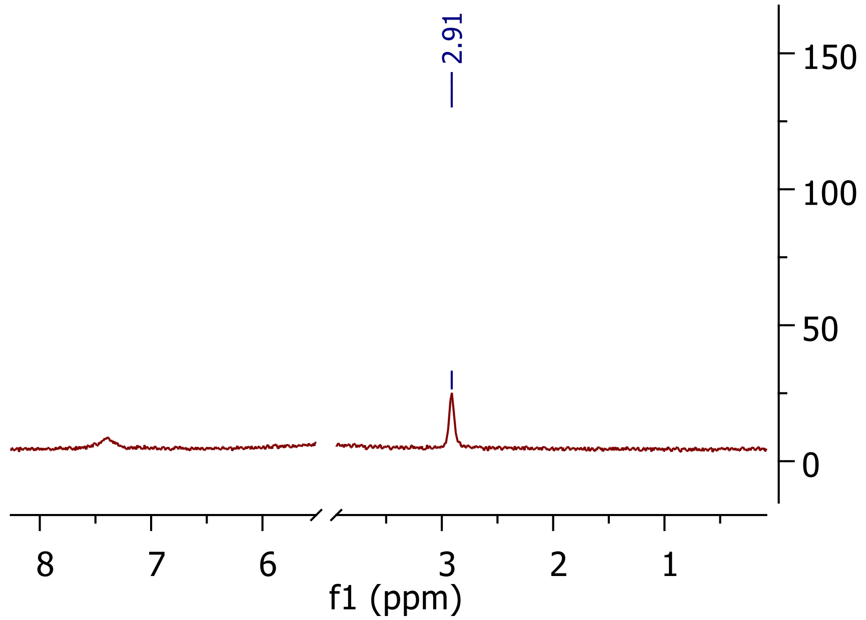

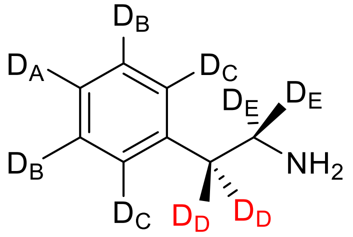

c. Mass spectrum

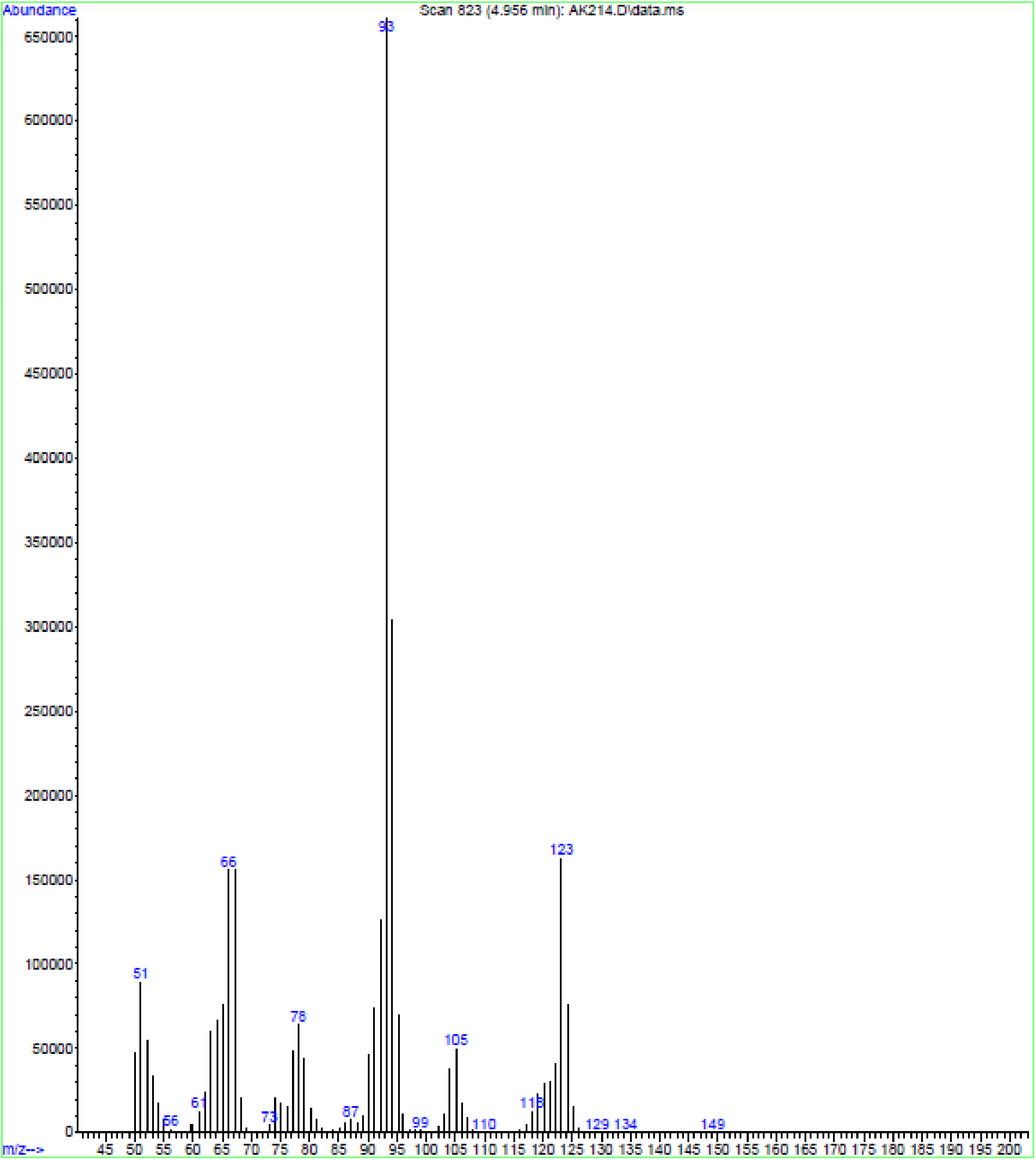

Figure S19. H-D exchange reaction of diethyl malonate

(20mg of Pd/C, MW, 60min, 100°C*)*

a. ^{1}H NMR

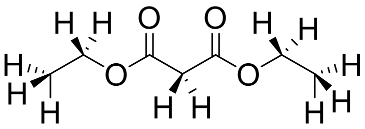

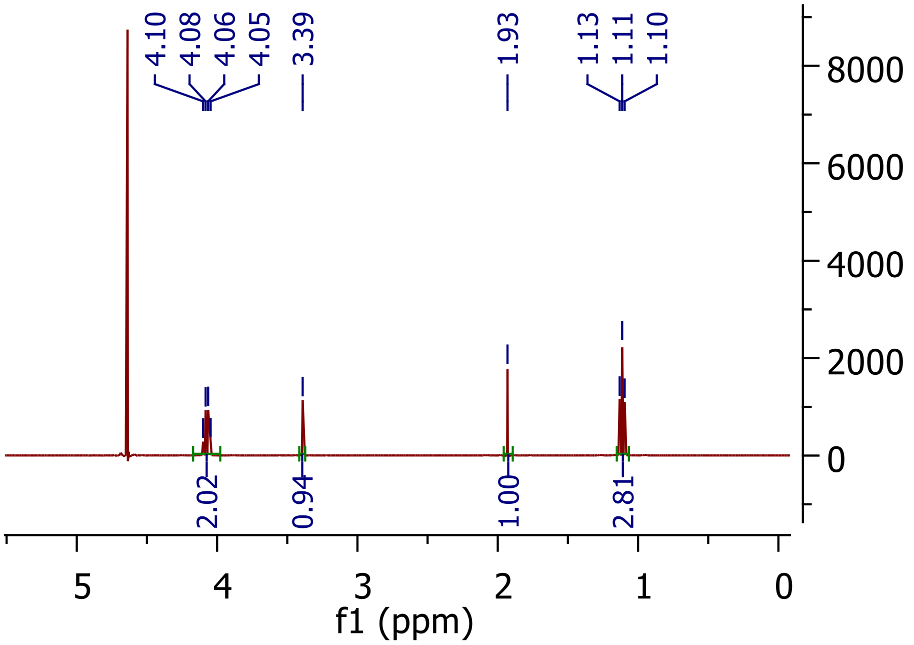

100mg Al

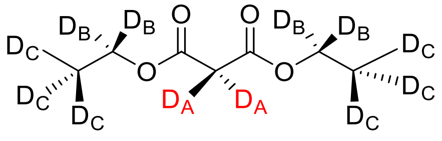

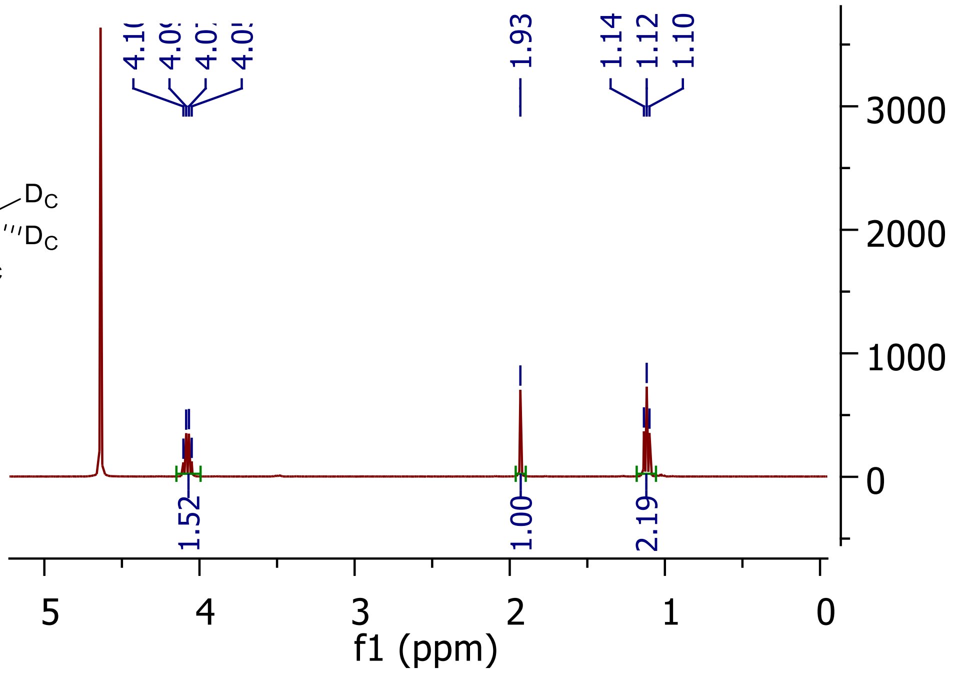

25mg Al (same yield)

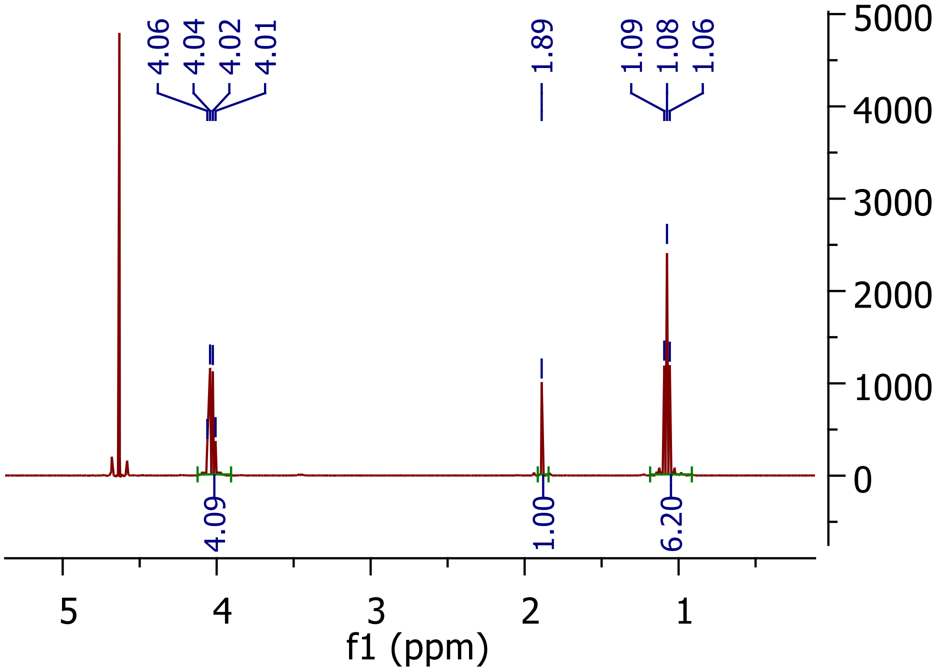

b. ^{2}H NMR

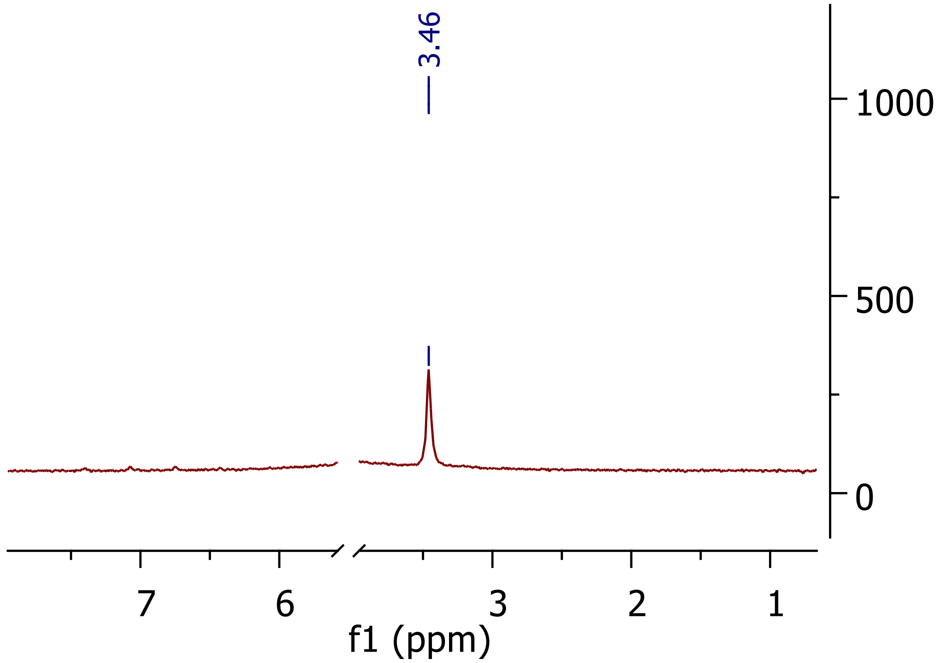

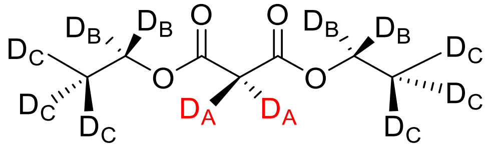

c. Mass spectrum

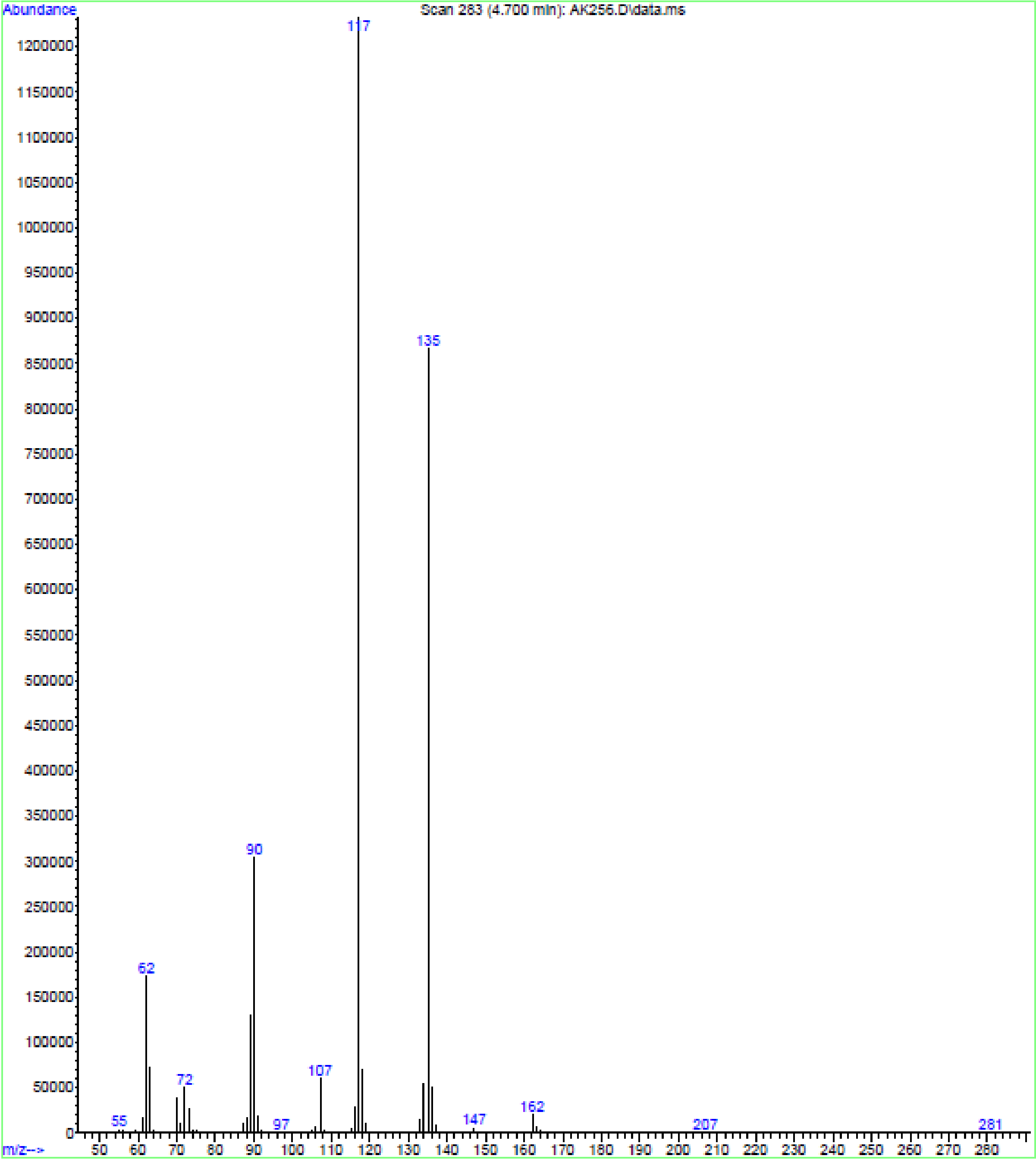

Figure S20. H-D exchange reaction of diethylmethyl malonate

(20mg of Pd/C, MW, 60min, 80°C*)*

a. ^{1}H NMR

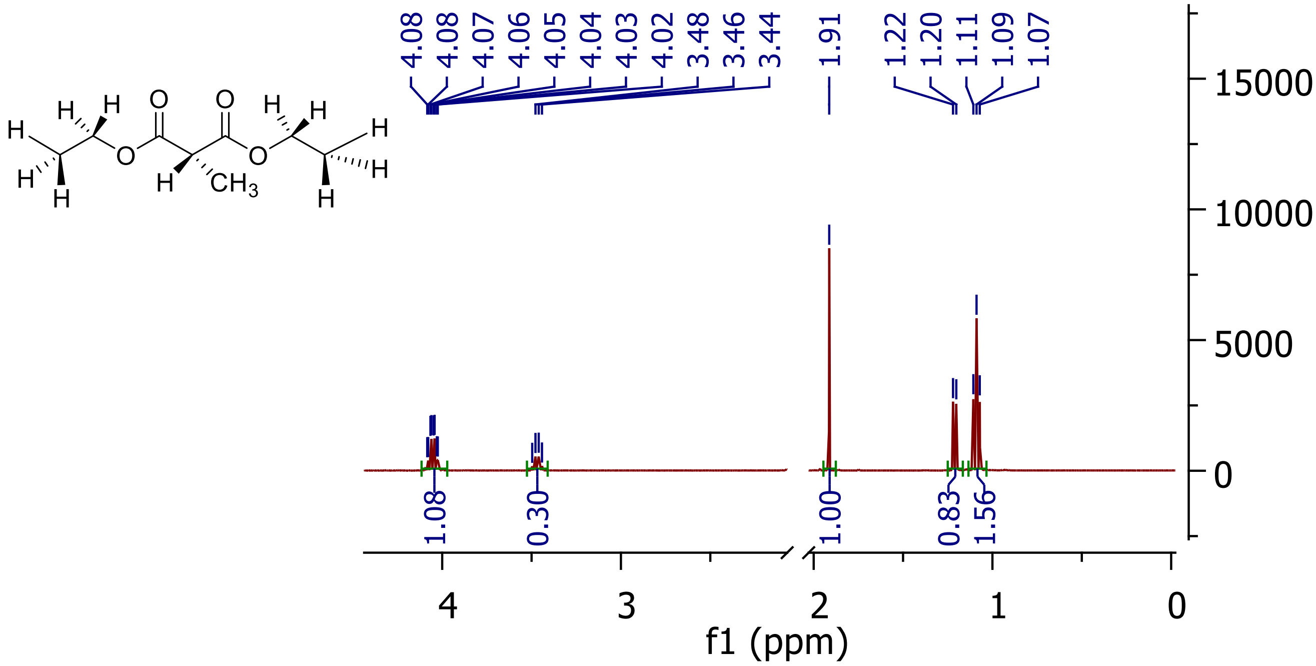

100mg Al

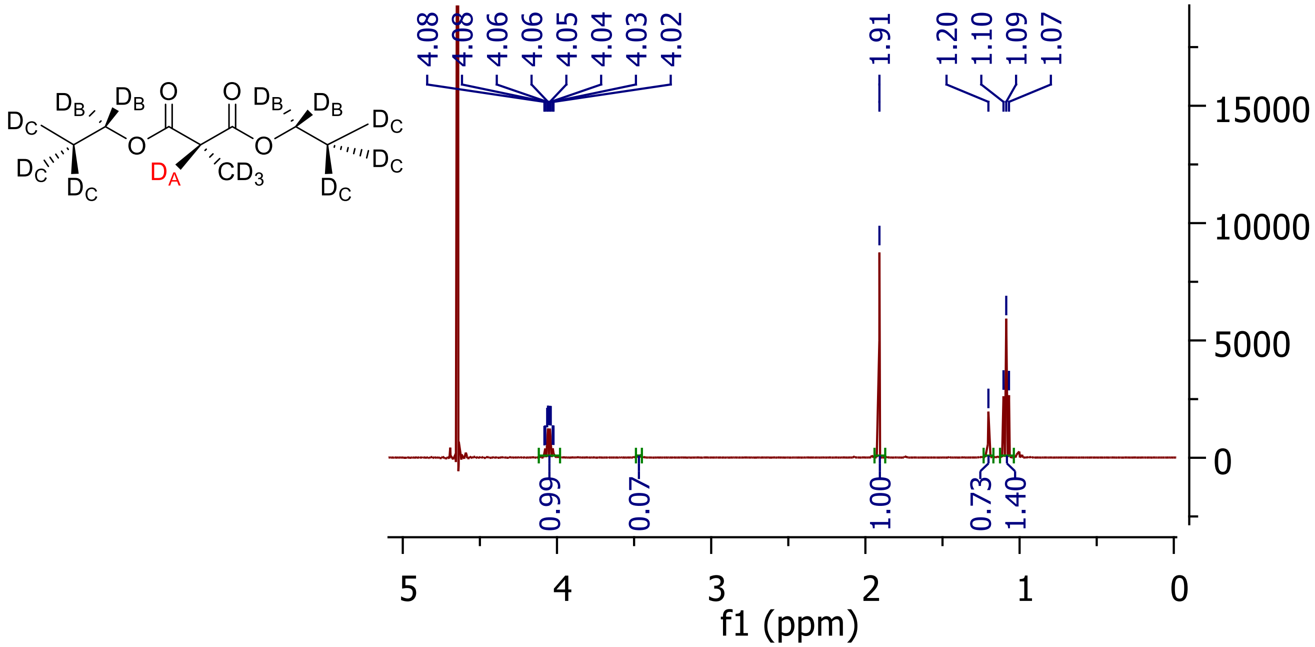

25mg Al (45% yield)

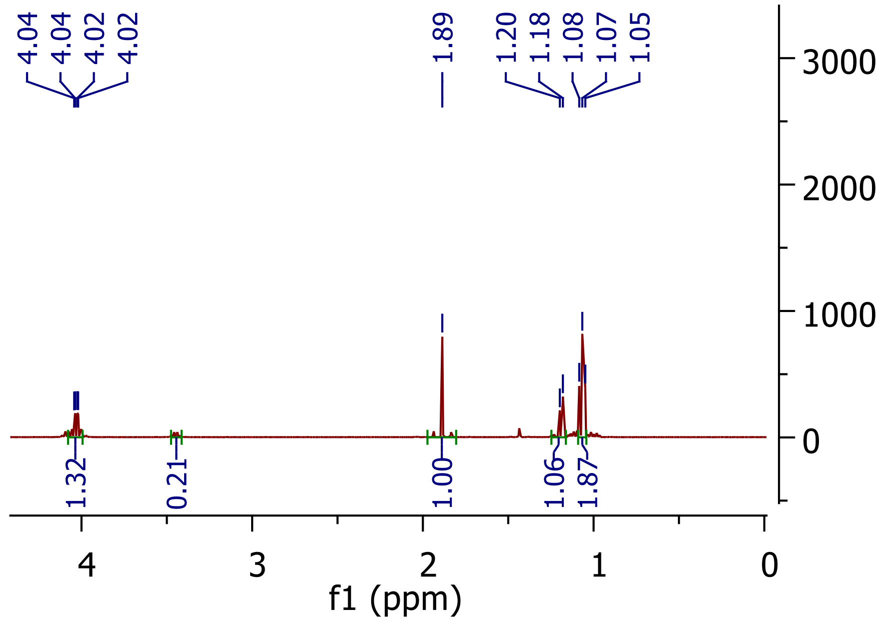

reference

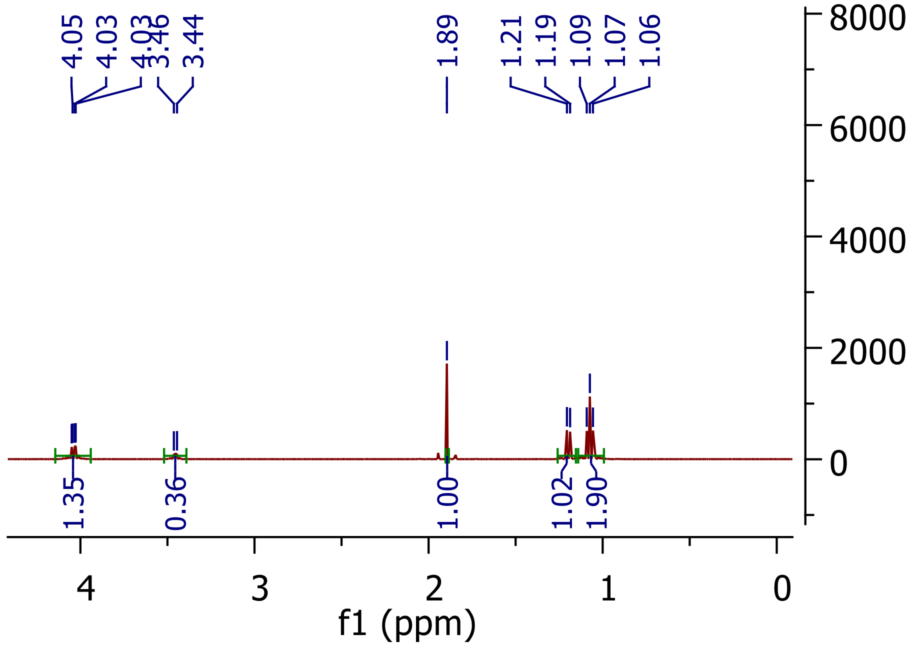

b. ²H NMR

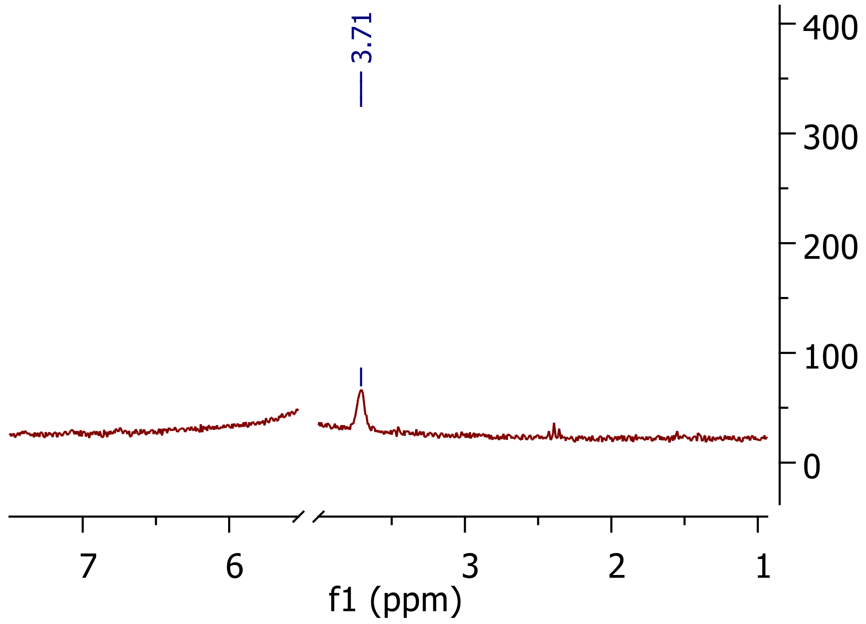
3.71
400
300
200
100
0
7
6
3
2
1
f1 (ppm)

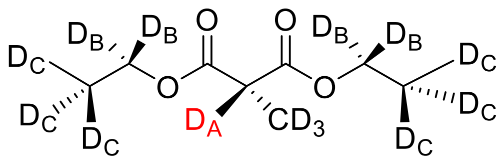
D_B
D_B
O
O
D_B
D_B
D_C
D_C
D_C
D_C
D_C
D_C
O
O
D_A
CD_3

c. Mass spectrum

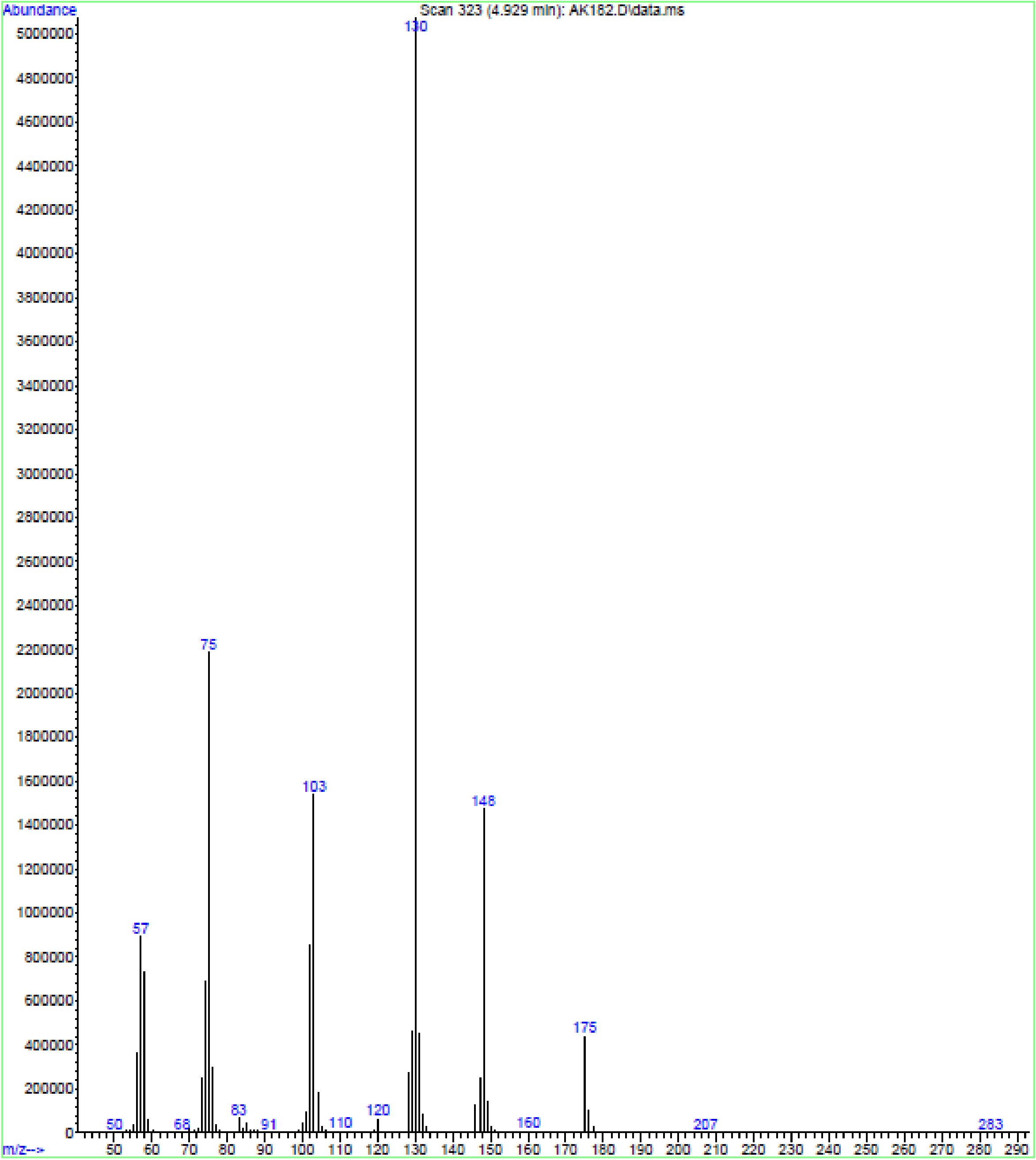

Figure S21. H-D exchange reaction of 3,5dihyroxybenzyl alcohol

(20mg of Pd/C, MW, 60min, 120°C*)*

a. ^{1}H NMR

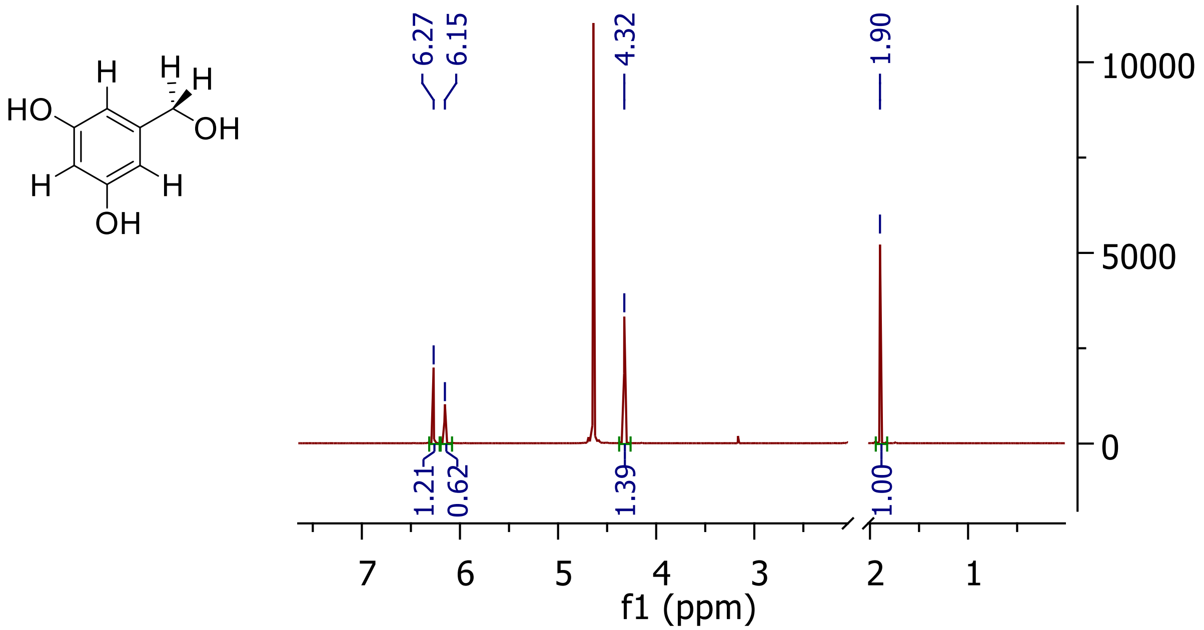

100mg Al

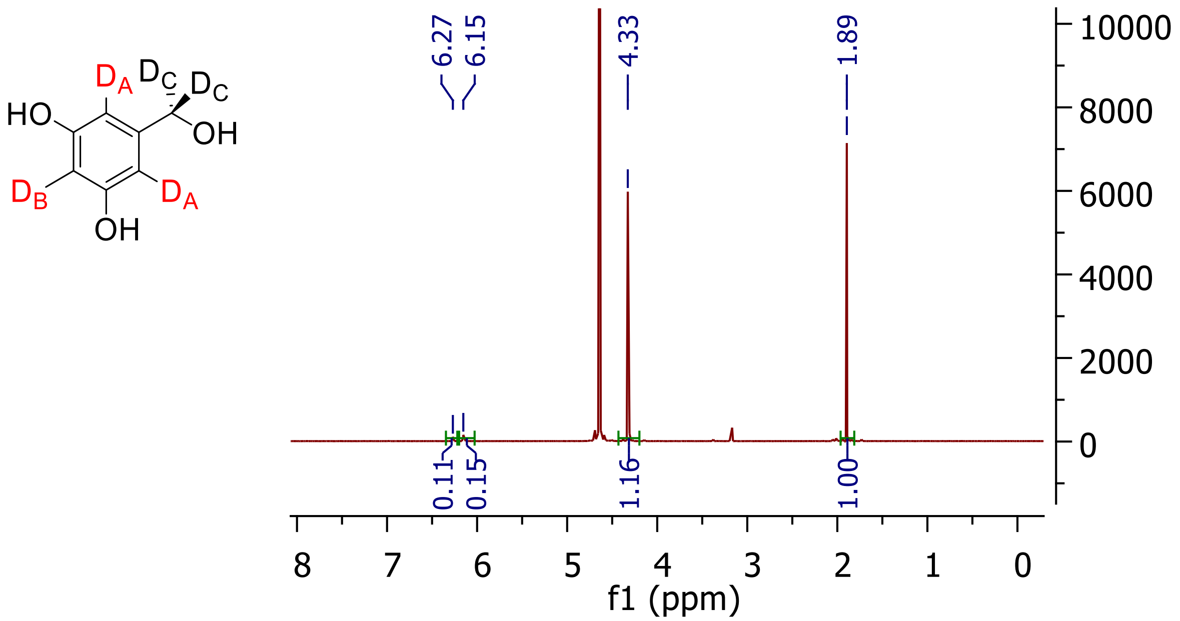

25mg Al (same yield)

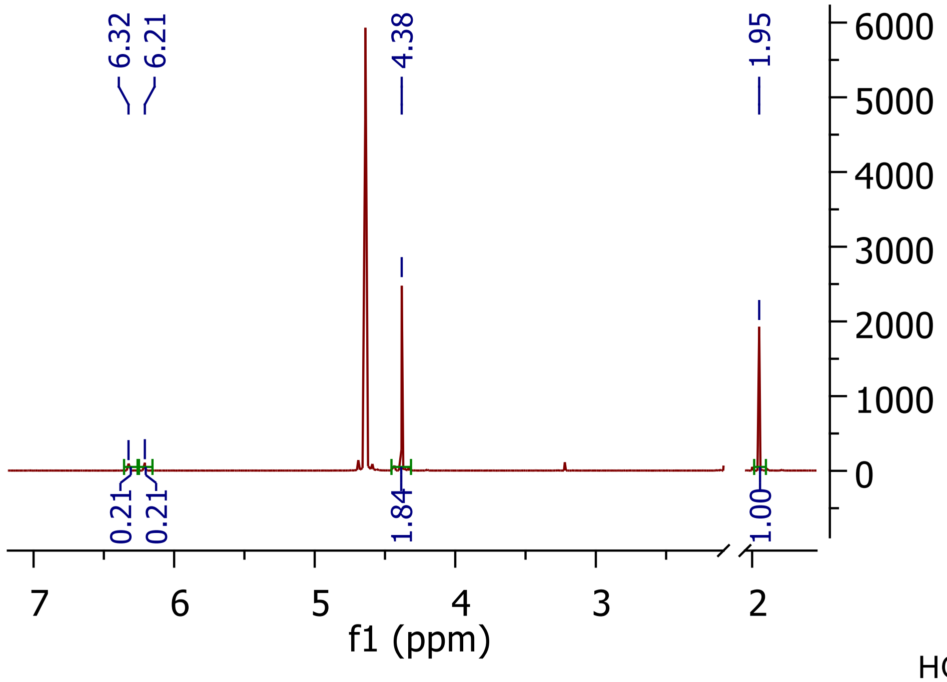

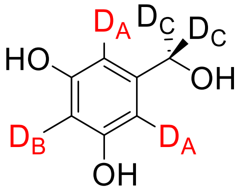

b. **^{2}H NMR**

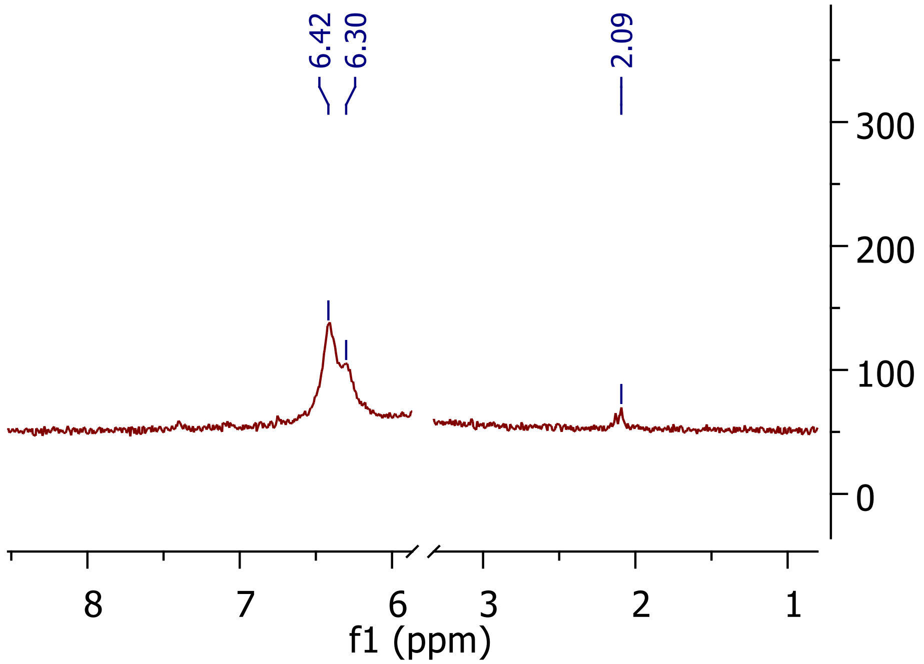

c. Mass spectrum

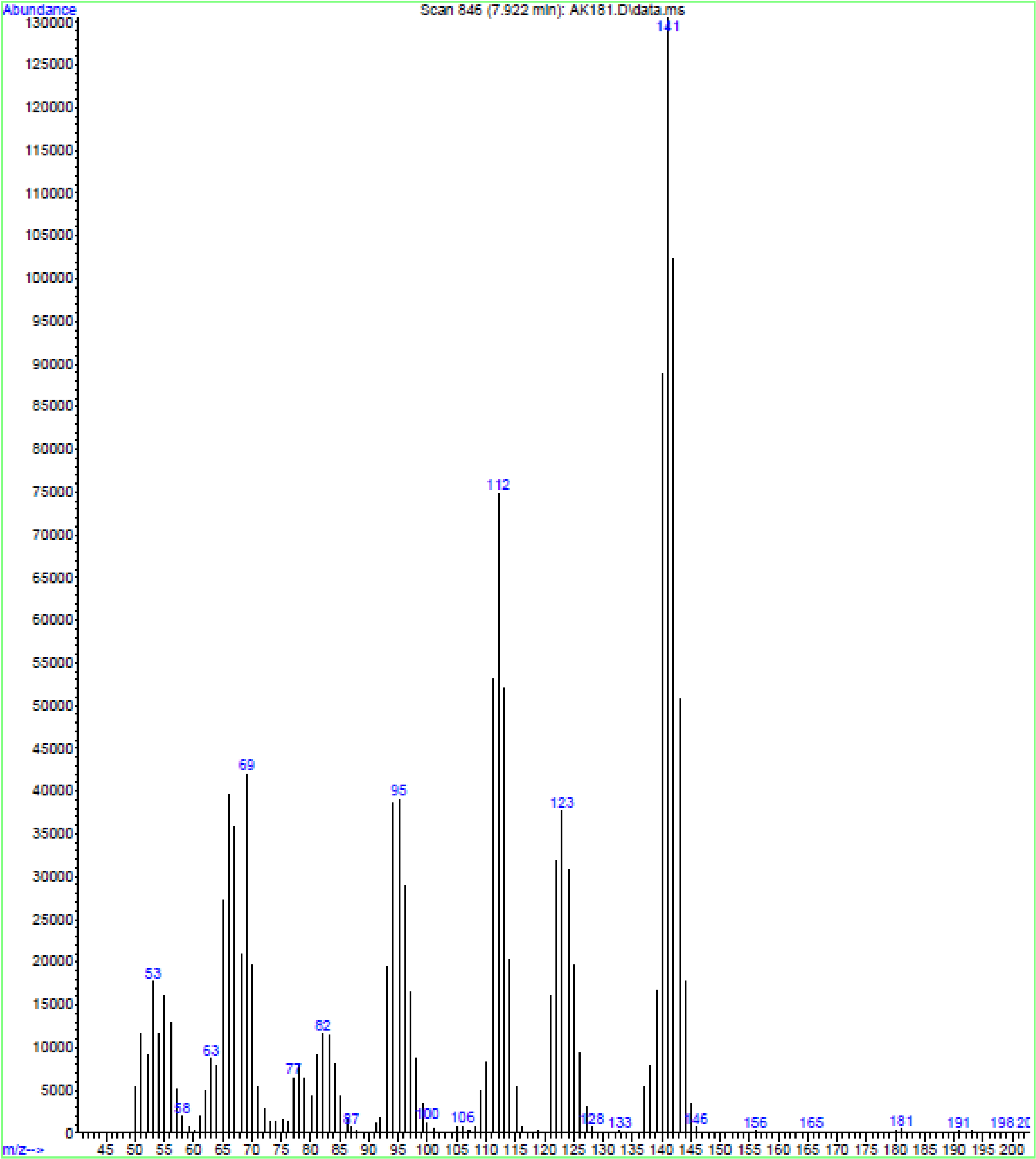

Figure S22. Recycling of the catalytic system

Reference

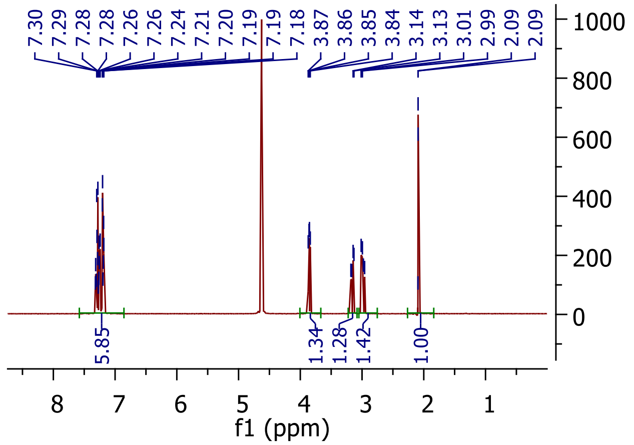

First cycle

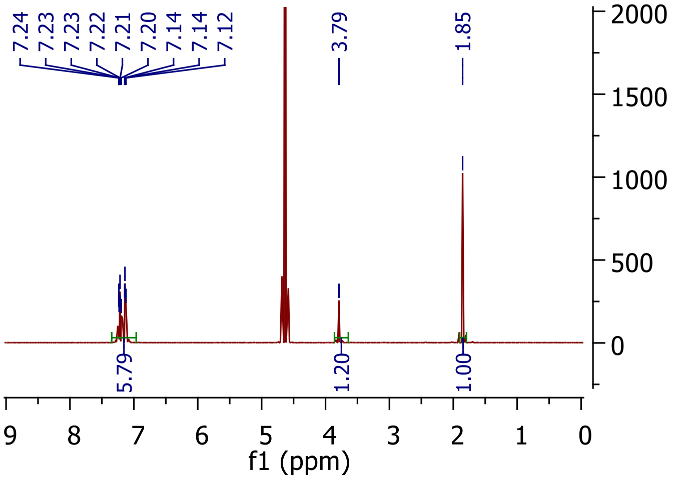

Second cycle

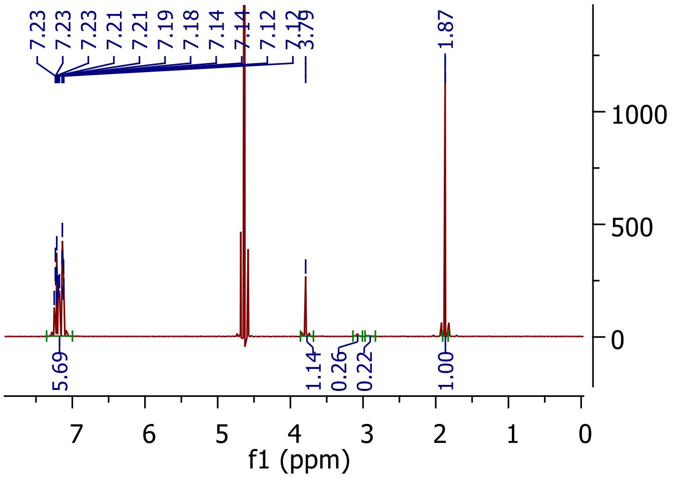

Third cycle

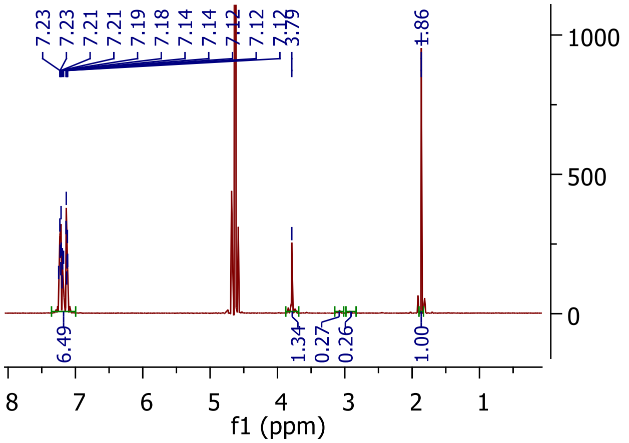

Fourth cycle

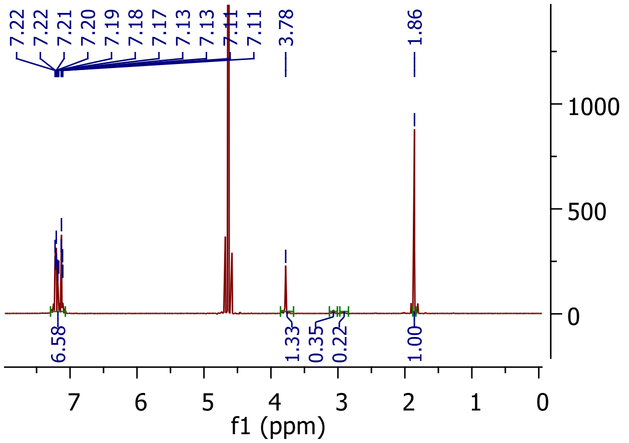

Fifth cycle

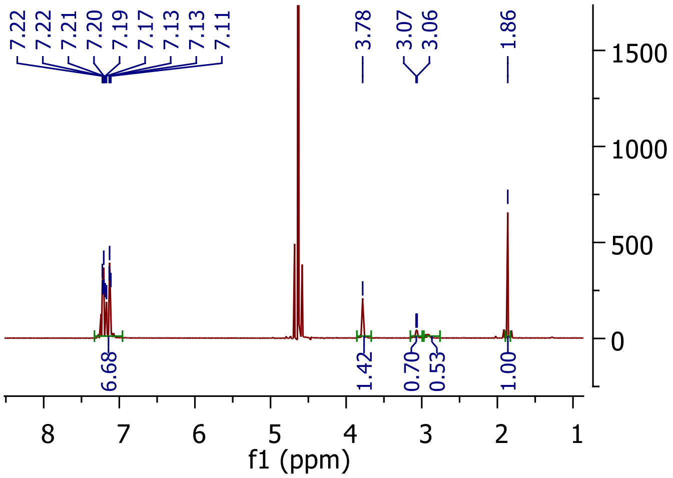

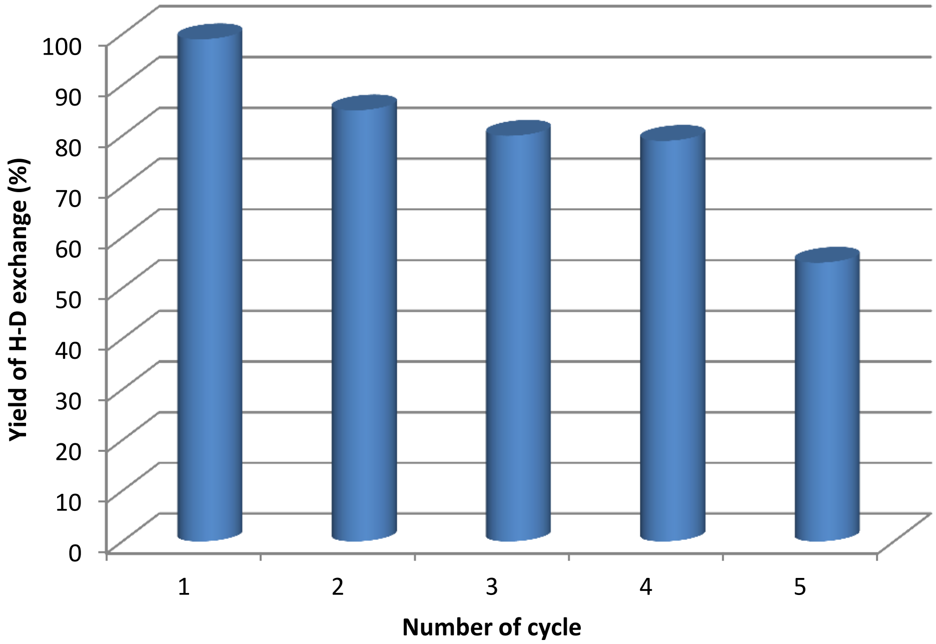

Number of cycle	Yield of H-D exchange (%)
1	100
2	85
3	80
4	79
5	55

Figure S23. %yield of H-D exchange for five consecutive reaction cycles
